# Azobisheteroarene photoswitches based on isoxazoles and pyrazoles: tunable photostationary states, thermal relaxation and sensitization under confinement

**DOI:** 10.1039/d5sc04412j

**Published:** 2025-09-18

**Authors:** Maximilian D. Seyfried, Julius Gemen, Leonard Wyszynski, Carl L. Giard, Constantin G. Daniliuc, Monika Schönhoff, Nikos L. Doltsinis, Frank Glorius, Bart Jan Ravoo

**Affiliations:** a Organic Chemistry Institute, University of Münster Corrensstr. 36 48149 Münster Germany; b Institute of Physical Chemistry, University of Münster Corrensstr. 28/30 48149 Münster Germany; c Institute of Solid State Theory and Center for Multiscale Theory and Computation, University of Münster Wilhelm-Klemm-Str. 10 48149 Münster Germany; d Center for Soft Nanoscience, University of Münster Busso-Peus-Str. 10 48149 Münster Germany b.j.ravoo@uni-muenster.de

## Abstract

Replacing one or both phenyl moieties of azobenzene with aromatic heterocycles is a versatile strategy to expand molecular diversity and to customize photophysical properties specifically for the intended application. Numerous mono-heteroaryl azo structures have been explored and characterized, with especially arylazopyrazoles (AAPs) and arylazoisoxazoles (AIZ) finding widespread application in various fields such as material science, photopharmacology and supramolecular chemistry. However, bis-heteroaryl motifs have been sparingly explored, with only a handful of examples known to date. Here, we introduce two previously unexplored classes of bisheteroaromatic structures: azobisisoxazole and isoxazoleazopyrazoles. Both classes readily undergo photoisomerization upon irradiation with UV (*E*–*Z*) and green light (*Z*–*E*), with half-life times of the *Z* isomer ranging from a few minutes to multiple months. Notably, some of the compounds combine very long half-life times of the *Z* isomer with near-quantitative photoisomerization in both directions. Furthermore, sensitized isomerization of these photoswitches under confinement enables rapid *E*–*Z* isomerization with selectable lower-energy photons, enabling high conversion even for derivatives performing poorly upon direct excitation. In general, these azobisheteroarenes represent new and easily accessible platforms for the design of light responsive molecules with favorable photophysical properties in photopharmacology and beyond.

## Abstract 2

Azo-type molecular photoswitches containing only pyrazoles and isoxazoles have numerous advantageous properties.

## Graphical abstract

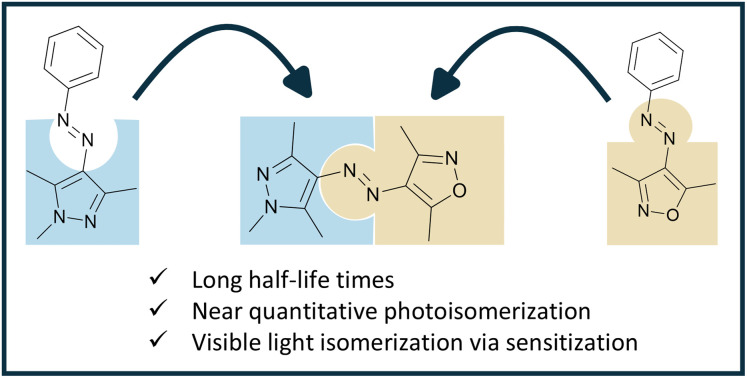

## Introduction

Molecular photoswitches, compounds that undergo reversible changes in their properties when irradiated with light of a specific wavelength, continue to draw interest from different scientific communities. Various types of photoswitches have been applied in a manifold of areas from photopharmacology and optoelectronics to material science and data storage.^1–6^ One of the oldest and most prominent classes of photochromic compounds are azobenzenes. Azobenzenes are readily available, can be switched with low light intensity, and exhibit a large change in end-to-end distance, dipole moment and geometry, making them versatile and easy to apply.^7^ Despite their widespread use, azobenzenes suffer some drawbacks, like incomplete conversion and limited stability of the *Z* isomer, limiting their practical application in some fields. To tackle these problems, different substitution patterns of the aromatic rings have been explored to improve and tune the photophysical properties. Tetra-*ortho*-fluorination, for example, drastically improves the half-life time of azobenzene to up to 700 days, while covalently bridging the aromatic rings in *ortho*-position *via* an ethylene linker results in near-quantitative isomerization in both directions.^8,9^ However, most substitution patterns increase the difficulty of the synthesis considerably. Another popular approach is to use arylazoheteroarenes instead of increasingly complicated azobenzene-derivatives.

In arylazoheteroarenes, one of the phenyl rings of azobenzene is replaced with an aromatic (often five-membered) heterocycle. Over the last two decades, numerous types of azoheteroarenes containing imidazoles,^10^ benzimidazoles,^11^ indoles,^12^ isoxazoles,^13^ pyrazoles,^14^ thiazoles,^15^ and others have been developed. These efforts have led to extensive libraries of heterocyclic photoswitches with a wide range of properties and diverse applications. However, combining accessibility, long half-life times and high photostationary states (PSS) in a single structure remains challenging. Two classes that have attracted significant attention are arylazopyrazoles (AAPs) and arylazoisoxazoles (AIZ). AAPs, introduced by Fuchter and coworkers,^14^ exhibit near-quantitative forward and reverse isomerization, with half-life times of the *Z* isomer of more than a week. Alternatively, derivatives exhibiting extremely long half-life times of multiple years but decreased photoconversion can be achieved through small modifications^16,17^ AAPs have been utilized in various applications such as facilitating light-induced gel–sol transitions in hydrogels,^18^ modifying the wettability of surfaces,^19^ storing energy in molecular solar thermal energy storage (MOST) devices,^20^ modulating drug activity in photopharmacology^21,22^ and supramolecular chemistry.^23^ AIZs on the other hand, often undergo a reversible solid-to-liquid phase transition when irradiated and have been successfully applied as light-responsive adhesives^24,25^ and for reversible surface patterning.^26^ Additionally, both isoxazoles and azoisoxazoles have found potential applications in medicinal chemistry. They have been shown to act as anti-inflammatory^27^ and anti-microbial^28,29^ agents as well as showing some potential in orally active anti-cancer agents.^30^ In contrast, bis-heteroaryl azophotoswitches have been considerably less explored. Recent reports include multiple azobisthiazole and azobisthiadiazole which demonstrate visible light photoswitching with decent to excellent conversion and half-life times ranging from minutes to days.^31^ Another notable contribution is the class of azobispyrazoles by Li and coworkers, which offers excellent photophysical properties including near-quantitative photoconversion and a very high *Z* isomer stability.^32^ To our knowledge, asymmetric azobisheteroarenes featuring two different types of heteroarenes are rarely documented and azobisheteroarenes containing isoxazoles have not been reported at all.

Here, we present a group of azobisisoxazoles and isoxazoleazopyrazoles as highly promising additions to the azobisheteroarene family and investigate their photophysical properties and thermal stability of the metastable *Z* isomer using NMR and UV/vis spectroscopy. Additionally, we demonstrate visible light induced *E*-to-*Z* isomerization based on the recently introduced concept of ‘disequilibration by sensitization under confinement’ (DESC).^33^ Sensitized visible light isomerization of these photoswitches not only broadens their applicability in materials and pharmacology but also boosts the performance of some derivatives with respect to photostationary states attainable.

## Results and discussion

### Synthesis

**Fig. 1:**
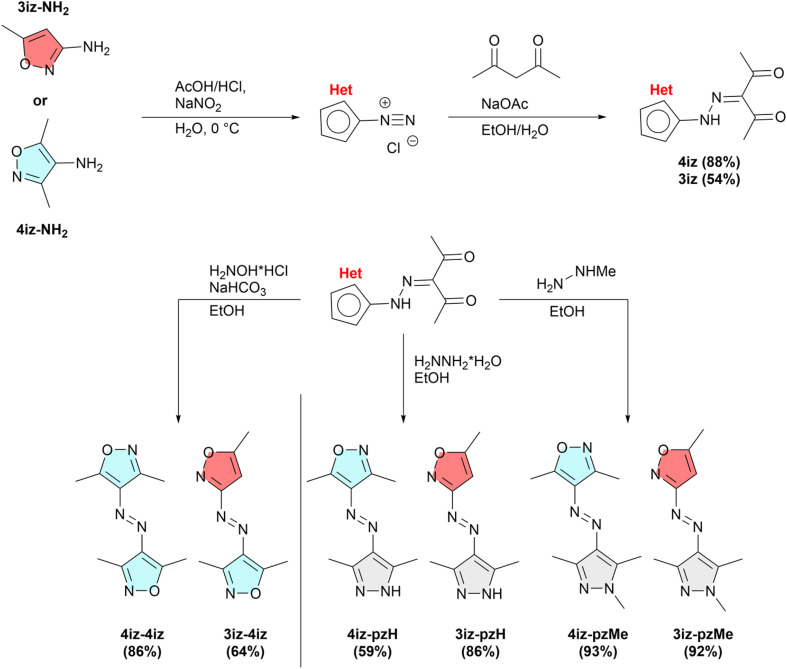
General synthesis scheme of azobisisxoazoles and isoxazoleazopyrazoles.

Six different azobisisoxazoles and azoisoxazolepyrazoles have been designed and synthesized through a straightforward two-step procedure, starting from the commercially available isoxazoleamines (see Fig. 1). The general synthesis route for all compounds is shown in Fig. 1. Similar to previous reports on arylazoheteroarenes,^13,14^ the amine starting material was first coupled with acetylacetone *via* diazo coupling to obtain the corresponding diketone in good to moderate yields (88% for 4iz-NH_2_ and 54% for 4iz-NH_2_), requiring only minimal purification.

However, it was observed that variations in the amount of acid and base used in this step would lead to significant differences in yield. Insufficient acidity results in the formation of a significant amount of the corresponding triazene side product in the diazotization, whereas an excess of acid considerably impedes the subsequent coupling with acetylacetone (see SI S2 and S3). The final compounds were then synthesized through a ring closure reaction using hydroxylamine hydrochloride, hydrazine or methylhydrazine to produce the azobisisoxazoles, isoxazoleazopyrazoles and methylated isoxazoleazopyrazoles in very good yields between 64% and 93%. Notably, the pyrazoles (4iz-pzH, 3iz-pzH) can easily be further modified on the pyrazole ring using existing methods.^34^ Details on the synthesis and analysis of all compounds can be found in the SI.

### Photophysical properties, photoisomerization and structure

**Fig. 2:**
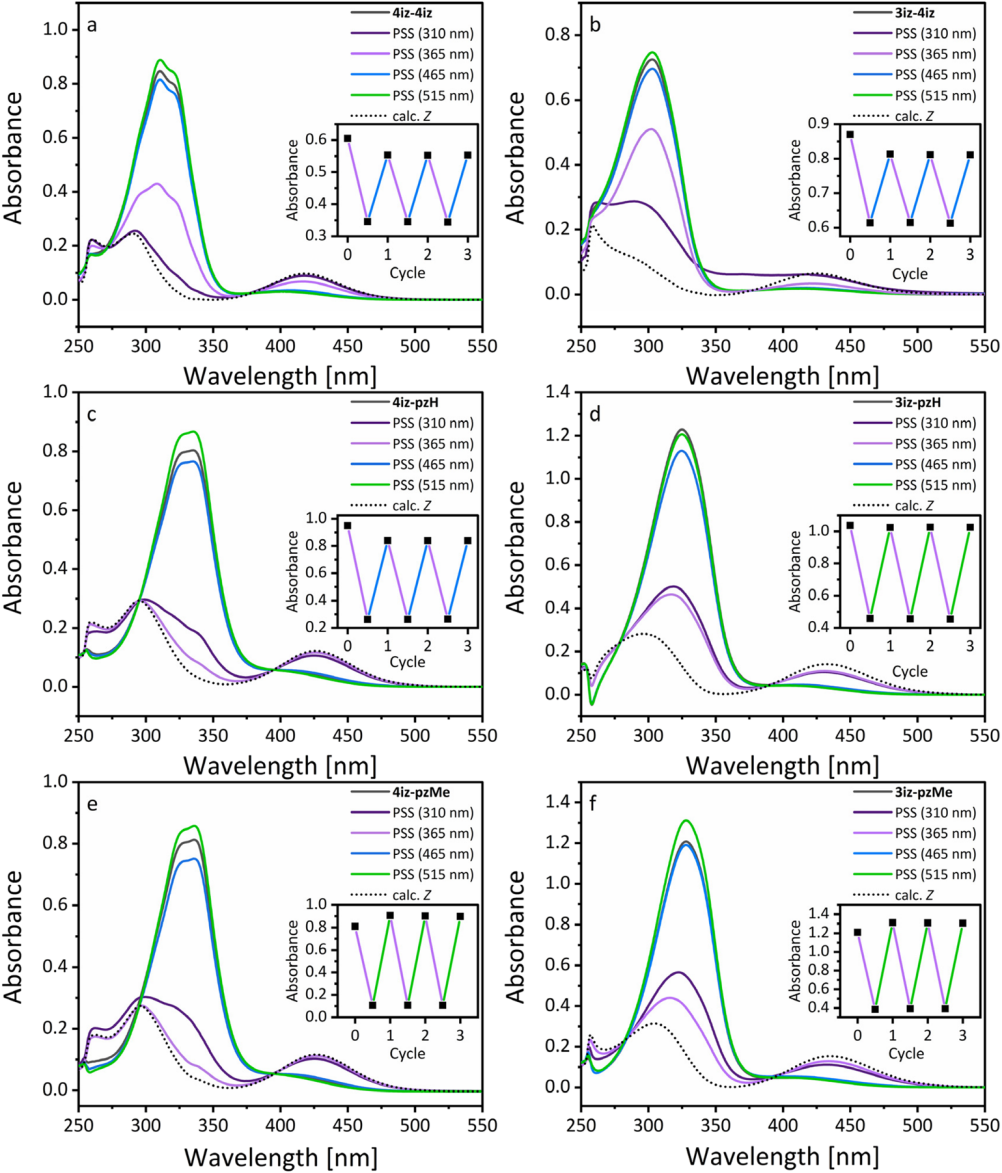
(a)–(f) UV/vis spectra of azobisisoxazoles and isoxazoleazopyrazoles in DMSO (50 μM) as synthesized (black) and after irradiation with 310 nm (20 s), 365 nm (1 min), 465 nm (1 min), and 515 nm (10 min) light, respectively. Inset: Absorbance at *λ*_max_ (π–π*) after photoswitching for multiple cycles with either UV and blue light or UV and green light as indicated by line color.

Initial studies of the photophysical properties were performed using UV/vis spectroscopy (see Fig. 2a–f). Under ambient light, all compounds exhibit a strong π–π* band (*ε*_max_ = 14.7 × 10^3^–22.5 × 10^3^) in the UV region (250–400 nm) and a weakly pronounced *n*–π* band in the visible region (400–500 nm). The azobisisoxazoles (4iz-4iz, 3iz-4iz) displayed a significant hypsochromic shift of the π–π* absorption band (311 nm, 303 nm) compared to the isoxazoleazopyrazoles due to the weaker electron-donating character of the isoxazole moiety (*σ*_ar^+^_ = 0.0 for 4-isoxazole *vs. σ*_ar^+^_ = −0.99 for *N*-methyl-4-pyrazole).^35^ The pyrazoles showed absorption maxima between 325 nm and 336 nm which is very similar to their arylazopyrazole counterparts.^16^*N*-Methylation of the pyrazole ring had a minimal effect on UV/vis absorption resulting in negligible shift of the absorption maxima. In all cases the 3-isoxazoles (3iz-4iz, 3iz-pzH, 3iz-pzMe) feature a significantly larger hypsochromic shift than 4-isoxazoles (4iz-4iz, 4iz-pzH, 4iz-pzMe).

When irradiated with UV light (365 nm), rapid *E*-to-*Z* isomerization was observed. This process can be monitored *via* UV/vis spectroscopy through a decrease in intensity and a hypsochromic shift of the π–π* band, accompanied by an increase in intensity and a bathochromic shift of the *n*–π* band. The PSS after irradiation was determined using ^1^H NMR spectroscopy (see SI, S18). Compounds 4iz-4iz, 3iz-4iz, 3iz-pzH and 3iz-pzMe achieved only partial *E*–*Z* conversion ranging from 38% to 62% due to a strong overlap of absorption for both isomers and blue-shifted absorption bands resulting in low absorption at the irradiation wavelength. For the azobisisoxazoles, irradiating closer to the absorption maxima with a wavelength of 310 nm achieved higher PSS for *E*–*Z*-isomerization, but resulted in degradation of the compound over time. Similar degradation but no improvement in PSS was observed for 4iz-pzMe. A possible cause of this degradation is the previously reported azirine formation from isoxazoles under short-wavelength UV irradiation.^36^ For the pyrazole derivatives irradiation with 310 nm led to no improvement in PSS compared to 365 nm. Pyrazoles 4iz-pzH and 4iz-PzMe, on the other hand, show nearly complete isomerization to the *Z* isomer using 365 nm (>86% and 92% respectively). Subsequent irradiation at the tail of the *n*–π* transition with green light (515 nm) resulted in near-quantitative back-isomerization (93–98%) to the *E* isomer for all compounds except 3iz-pzMe (84%) due to the near-zero absorption of *E* isomers at wavelengths above 500 nm. Isomerization with green light for *Z*-isoxazoleazopyrazoles proceeded quickly, while the lower absorption in the green region for *Z*-azobisisoxazoles led to comparatively slow isomerization. Irradiating closer to the peak of the *n*–π* band with blue light (465 nm) resulted in faster isomerization but only limited conversion due to the large overlap in absorption for *Z* und *E* isomers in this region. Over three photoswitching cycles, no degradation was observed. With its near-quantitative isomerization in both directions, 4iz-pzMe not only matched the performance of some of the best-performing azobenzene derivatives like 2,2′,6,6′-tetrafluoroazobenzene^8^ and the ethylene-bridged diazocine^9^ but also surpasses most representatives of mono- or bisheteroaryl azo photoswitches like arylazoisoxazoles,^13^ arylazothiazoles, azobisthiazoles and arylazothiadiazoles.^31^

Subsequently, the thermal *Z*–*E*-isomerization in DMSO was studied and half-life times of the *Z* isomer (*t*_1/2_) were determined by following the change in absorbance over time *via* UV/vis spectroscopy after initial irradiation with 365 nm (see Table 1). For compounds with *t*_1/2_ greater than one day, half-life times were measured at multiple elevated temperatures (70–90 °C) and extrapolated to 25 °C using Eyring plots (see Fig. S25). The non-methylated isoxazoleazopyrazoles exhibit thermal relaxation within minutes to hours (*t*_1/2_ = 127 min and 24 min for 4iz-pzH and 3iz-pzH, respectively), while the methylated pyrazoles show at least an 1800-fold increase in half-life time to over three months. This large difference between the methylated and non-methylated pyrazoles has been observed previously in arylazopyrazoles and can be attributed to hydrogen bonding and tautomerism to the hydrazone form. This tautomerism is only available to the non-*N*-substituted pyrazoles and drastically decreases the life time of the *Z* isomer.^10,37^ Azobisisoxazoles 4iz-4iz and 3iz-4iz exhibit half-life times in between *N*-methylated and non-methylated pyrazoles (91 days and 50 hours respectively). For azobisisoxazoles (4iz-4iz, 3iz-4iz) and NH-pyrazoles (4iz-pzH, 3iz-pzH), the half-life times of 3-isoxazoles are considerably shorter than those of the 4-isoxazoles. 4iz-pzMe and 3iz-pzMe on the other hand show only a small difference.

**Table 1:** Summary of photophysical characteristics and thermal half-life times in DMSO

Molecule	*λ*_max_π–π (*E*)/nm	*λ*_max_ *n*–π (*E*)/nm	*λ*_max_π–π (*Z*)/nm	*λ*_max_ *n*–π (*Z*)/nm	Photoconversion		*t*_1/2_ (25 °C)	*ε*_max_/(cm^−1^ M^−1^)
					*E*-to-*Z* (365 nm)	*Z*-to-*E* (515 nm)		
4iz-4iz	311	403	292	418	57%	95%	91 d^c^	17.3 × 10^3^
3iz-4iz	303	413	303	420	38%	94%	50 h^c^	14.7 × 10^3^
4iz-pzH	335	n.d^d^	296	426	86 %^a^	98%	127 min	20.7 × 10^3^
3iz-pzH	325	n.d^d^	320	431	62 %^a^^,^^b^	99%	24 min	22.5 × 10^3^
4iz-pzMe	336	n.d^d^	297	426	92%	97%	98 d^c^	17.7 × 10^3^
3iz-pzMe	328	n.d^d^	316	434	52%	84%	102 d^c^	26.2 × 10^3^

a Sample was measured under continuous irradiation.

b Sample measured in CDCl_3_ instead of DMSO-d_6_ due to limited solubility.

c Extrapolated to 25 °C from measurements at 70 °C, 75 °C, 80 °C, 85 °C and 90 °C.

d Spectrum shows no distinct *n*–π* band for the *E*-isomer due to overlap with π–π* band.

Different applications of molecular photoswitches often require different solvents. Especially in biology and pharmacology, water is the solvent of choice. Therefore, the performance of the bisazoheteroarenes in aqueous solution was investigated (see Fig. S26). Although poorly soluble in pure water, most compounds readily dissolve in a mixture of water and DMSO with as little as 10% (*v*/*v*) DMSO. In this solvent mixture, all compounds exhibit a blue-shift of the absorption bands of 2–10 nm. 3iz-4iz exhibits a very low water solubility and while photoisomerization still occurs, precipitation of the photoswitch was observed in concentrations as low as 25 μM, rendering it basically non-applicable in aqueous media without high concentrations of organic solvents present. For 3iz-pzH, no detectable amount of the *Z* isomer was observed after irradiation. This can be explained by a significantly increased rate of thermal relaxation *via* mechanisms such as tautomerism in aqueous media, leading to a near complete relaxation to the *E* isomer within seconds.^37^4iz-4iz underwent isomerization but reached lower PSS in the solvent mixture compared to pure DMSO due to a strong blue-shift of the *n*–π* band, decreasing the absorption of the *E* isomer at 365 nm even further. Meanwhile, 4iz-pzH, 4iz-pzMe and 3iz-pzMe were barely affected by the change in solvent. Even though small changes in UV/vis absorption were observed, photoisomerization similar to a solution in DMSO was demonstrated.

**Fig. 3:**
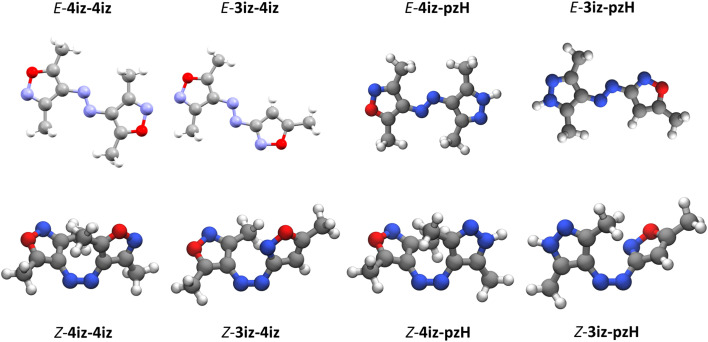
Crystal structures of *E*-4iz-4iz and *E*-3iz-4iz and calculated geometries of the lowest energy conformer for *E*-4iz-pzH, *E*-3iz-pzH and *Z* isomers of all compounds.

To provide insights into the molecular structure of the synthesized compounds, single crystal X-ray crystallography structures were obtained for *E*-4iz-4iz and *E*-3iz-4iz. For the isoxazoleazopyrazoles and all *Z* isomers, no single crystals could be obtained. Therefore, geometry optimization *via* density functional theory (DFT) was performed instead (see Fig. 3 and S36–S43). For compounds *E*-4iz-4iz and *E*-3iz-4iz the obtained crystal structures are in very good agreement with the calculated lowest energy conformer.

Interestingly, all *E* isomers display a perfectly planar structure, independent of the substitution pattern. This finding is in good agreement with the observed UV/vis spectra showing only a weak *n*–π* transition for the *E* isomer.^38^

In comparison, tetra-*ortho*-methylated azobenzenes and arylazopyrazoles rotate away from coplanarity with the azo group to reduce steric repulsion, resulting in a twisted geometry.^32^ However, the two smaller five-membered rings offer enough space to accommodate the four *ortho*-methyl groups without twisting. Similar to azobenzene, the *Z* isomers adopt a twisted geometry for all compounds. This twisted geometry breaks the symmetry of the molecule and is responsible for a strong increase in the otherwise symmetry-forbidden *n*–π* transition, which allows the near-complete *Z*–*E*-isomerization.^38^ Other possible geometries, such as the T-shape commonly found in Ph-N-N-Het-type photoswitches without *ortho*-substituents^15,16^ or the rare planar *Z* isomer found in some azobispyrazoles^32^ were not observed. *N*-methylation of the pyrazole rings leads to no striking difference in structure in all investigated molecular photoswitches (see Fig. S42/S43). Subsequent calculation of the dipole moments (see Table S4) shows that most compounds only exhibit a relatively small change in dipole moments when irradiated (0.3 D–1.3 D). Interestingly, unlike in azobenzene, the dipole moment slightly decreases for multiple compounds when isomerized, making the *Z* isomers less polar.

### Disequilibration by sensitization under confinement

**Fig. 4:**
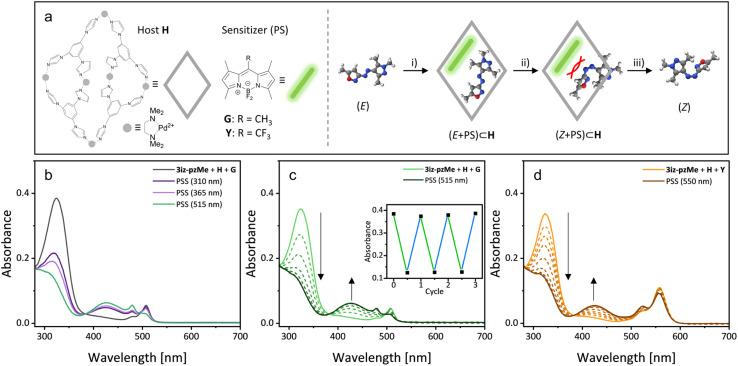
(a) Structures of host H, sensitizers Y and G and a simplified scheme of the DESC mechanism of 3iz-pzMe: (i) Formation of the ternary inclusion complex (*E* + PS) ⊂ H; (ii) Isomerization of the photoswitch in the confined space through an energy transfer mechanism enabled by visible light excitation of the PS; (iii) Disassembly of the unstable (*Z* + PS) ⊂ H. (b) Comparison of direct excitation (310 nm, 15 s; 365 nm, 5 s); DESC (515 nm, 1 min) in water using G (20 μM 3iz-pzMe, 0.5 eq. H, 0.05 eq. G). (c) Time course of *E*–*Z* isomerization over 150 s using green light (20 μM 3iz-pzMe, 0.5 eq. H, 0.05 eq. G). Inset: Alternating photoswitching using blue (445 nm) and green (515 nm) light. (d) Time course of *E*–*Z* isomerization over 15 min using yellow light (20 μM 3iz-pzMe, 0.5 eq. H, 0.2 eq. Y).

The lack of visible light *E*-to-*Z* isomerization for all synthesized derivatives, as well as the poor photostationary states observed in some cases, remained inherent limitations of the azobisheteroaryl photoswitches described above. To address these limitations, we turned our attention to the recently introduced concept of ‘disequilibration by sensitization under confinement’ (DESC). In this supramolecular approach, *E*-to-*Z* isomerization occurs not *via* direct π–π* excitation, but through triplet energy transfer from visible light absorbing photosensitizers (see Fig. 4a).^33^ To achieve excitation *via* energy transfer, sensitizer and photoswitch were both encapsulated as homodimers in a water-soluble supramolecular host (H, see Fig. 4a). Upon mixing of both species, a ternary heterodimer complex (*E* + PS) ⊂ H consisting of the sensitizer, the *E* photoswitch and H is formed. In this inclusion complex, the sensitizer can absorb visible light and undergo efficient intersystem crossing followed by triplet-energy transfer from the sensitizer to the photoswitch.^39^ The restricted cavity size of H ensures that only the sterically less demanding *E* isomer of the switch is effectively sensitized, leading to its rapid depletion and accumulation of the *Z* isomer. A more detailed description of the photocatalytic DESC mechanism tailored for the present study is shown in the SI (SI, S28).

Following a reported procedure,^33^ we initially screened the responsiveness of all six *E* derivatives to green light in the presence of host H and the green light absorbing dye G (see Fig. 4a).^40^ Among the tested derivatives, 4iz-pzMe, 4iz-pzH, and 3iz-pzMe exhibited rapid and near-quantitative isomerization, while 3iz-4iz showed only a limited response. However, the limited response of 3iz-4iz could be drastically improved under more optimized conditions. Using higher concentrations (8 mM, 5 mol% G), over 75% *E*-to-*Z* isomerization was achieved using green light (see Fig. S32–S34), roughly doubling conversion in comparison to the directly UV-excited mechanism. The concentration dependency is probably connected with a relatively low binding affinity to host H, slowing the DESC-related *E*-to-*Z* conversion and relatively increasing the rate of the competing π–π* excitation of the *Z* isomer. By increasing concentration or sensitizer loading, the rate of DESC-based isomerization can be increased while the rate of direct excitation remains largely unaffected, favoring accumulation of *Z* isomer. For 3iz-pzH, heterodimer complex formation with G was observed. However, due to its rapid thermal *Z*–*E*-isomerization in aq. solution, no significant amount of *Z* isomer was detected (see Fig. S29). Compound 4iz-4iz showed no interaction with the host H and is therefore unsuitable for DESC using the host H. Due to its relatively poor response to both 310 nm and 365 nm light (see Fig. 2 and Table 1) but good initial results under DESC conditions, we focused on 3iz-pzMe for a more detailed study.

A sample of 3iz-pzMe in water, solubilized by the addition of 0.5 eq. of host H, was initially irradiated with 310 nm and 365 nm to achieve the respective poor PSS (52% for 365 nm, *ca.* 45% for 310 nm) *via* direct excitation. Irradiation with green light (515 nm) in the absence of a photosensitizer resulted in near-quantitative formation of the *E* isomer. Subsequent addition of 1.0 equivalent of G encapsulated in H (see Fig. 4a) and continued green light irradiation led to rapid accumulation of *Z*-3iz-pzMe instead (see Fig. 4c). Importantly, while the *Z*-to-*E* isomerization of the photoswitch induced by green light remains active, it is significantly outpaced by DESC-based disequilibration mechanism, hence allowing for a high PSS.

To further investigate the supramolecular system, we examined the influence of the applied amount of G on the isomerization. We observed near-quantitative formation of *Z*-3iz-pzMe down to 0.05 eq. of photosensitizer (see Fig. 4d), with a strong response still evident with only 1 mol% of G (see Fig. S29), although isomerization proceeded slightly slower (5 min at 1 mol% *vs.* 2 min at 5 mol%). With only 5 mol% of G, a PSS of nearly 80% was achieved on NMR scale (8 mM 3iz-pzMe, see Fig. S35–S37). Reversible switching between the *E* and *Z* states using only visible light was possible, as the DESC-based green light isomerization could be reversed with blue light (445 nm; see Fig. 4d inlet). Finally, we explored the use of other photosensitizers absorbing light of lower energy for 3iz-pzMe isomerization. Application of sensitizer Y enabled *E*-to-*Z* transformation under yellow light (550 nm; see Fig. 4e). While the behavior was essentially analogous to that of G, higher loadings of sensitizer (0.1 eq. or higher) and longer irradiation times were required to achieve similar PSSs, likely due to Y's lower triplet energy.^31^

## Conclusion

In this work, we introduce six derivatives of two novel, readily synthesized classes of azobisheteroaryl photoswitches featuring isoxazole and pyrazole heterocycles: isoxazoleazopyrazoles and azobisisoxazoles. An examination of their switching behavior and photophysical properties revealed a wide range of half-life times and photostationary states. Compound 4iz-pzMe performed especially well by exhibiting near-quantitative back- and forward-isomerization, a very high thermal stability of the *Z*-isomer and nearly unaffected switching behavior in aqueous solution. The combination of these properties makes it a very promising addition to the photoswitch toolkit and a great candidate for further applications in material science and photopharmacology. While compounds 4iz-4iz and 3iz-pzMe exhibit very long half-life times as well, they only achieve partial photoconversion. For applications requiring faster relaxation, 4iz-pzH provides high photostationary states with a half-life time of just over one hour. Comparing 3- and 4-isoxazoles, we found that 3-isoxazoles typically display inferior photophysical properties, with significantly reduced photoconversion and often decreased thermal stability of the *Z* isomer. *E*-to-*Z* isomerization with visible light was achieved for selected photoswitch derivatives based on DESC (disequilibration by sensitization under confinement). Sensitized isomerization not only allowed replacing the high energy UV light sources with green or yellow light, potentially allowing for their integration into more complex environments, but also improved the photostationary states obtainable.

## Author contributions

M.D.S. synthesized and characterized the compounds and determined the photophysical properties. J.G. performed UV/vis and NMR experiments under DESC conditions. B.J.R. conceptualized and supervised the study. C.G.D measured and refined the single crystal structures. L.W. performed NMR measurements under *in situ* irradiation. C.L.G and N.L.D. carried out DFT calculations. The original draft was written by M.D.S. and J.G. with input from all authors. All authors discussed the results and reviewed and edited the manuscript.

## Conflicts of interest

There are no conflicts to declare.

## Supplementary Material

SC-OLF-D5SC04412J-s001

SC-OLF-D5SC04412J-s002

## Data Availability

Deposition numbers 2463597 (for *E*-4iz-4iz) and 2463598 (for *E*-3iz-4iz) contain the supplementary crystallographic data for this paper. CCDC 2463597 and 2463598 contain the supplementary crystallographic data for this paper.^41^ The data supporting this article have been included as part of the SI. Supplementary information: synthesis and spectra of photoswitches and intermediates, spectroscopic data of photoisomerization, computational details, crystallographic data. See DOI: https://doi.org/10.1039/d5sc04412j.

## References

[cit1] Yang X., Zhou X., Qin X., Liang D., Dong X., Ji H., Wen S., Du L., Li M. (2024). ACS Pharmacol. Transl. Sci..

[cit2] Corrado F., Bruno U., Prato M., Carella A., Criscuolo V., Massaro A., Pavone M., Muñoz-García A. B., Forti S., Coletti C., Bettucci O., Santoro F. (2023). Nat. Commun..

[cit3] Prasetya N., Ladewig B. P. (2018). ACS Appl. Mater. Interfaces.

[cit4] Gindre D., Boeglin A., Fort A., Mager L., Dorkenoo K. D. (2006). Opt. Express.

[cit5] Li X., Cho S., Wan J., Han G. G. D. (2023). Chem.

[cit6] Gonzalez A., Qiu Q., Usuba J., Wan J., Han G. G. D. (2024). ACS Mater. Au.

[cit7] Beharry A. A., Andrew Woolley G. (2011). Chem. Soc. Rev..

[cit8] Bléger D., Schwarz J., Brouwer A. M., Hecht S. (2012). J. Am. Chem. Soc..

[cit9] Siewertsen R., Neumann H., Buchheim-Stehn B., Herges R., Näther C., Renth F., Temps F. (2009). J. Am. Chem. Soc..

[cit10] Otsuki J., Suwa K., Sarker K. K., Sinha C. (2007). J. Phys. Chem. A.

[cit11] Steinmüller S. A. M., Odaybat M., Galli G., Prischich D., Fuchter M. J., Decker M. (2024). Chem. Sci..

[cit12] Simeth N. A., Crespi S., Fagnoni M., König B. (2018). J. Am. Chem. Soc..

[cit13] Kumar P., Srivastava A., Sah C., Devi S., Venkataramani S. (2019). Chem.–Eur. J..

[cit14] Weston C. E., Richardson R. D., Haycock P. R., White A. J. P., Fuchter M. J. (2014). J. Am. Chem. Soc..

[cit15] Lin R., Hashim P. K., Sahu S., Amrutha A. S., Cheruthu N. M., Thazhathethil S., Takahashi K., Nakamura T., Kikukawa T., Tamaoki N. (2023). J. Am. Chem. Soc..

[cit16] Calbo J., Weston C. E., White A. J. P., Rzepa H. S., Contreras-García J., Fuchter M. J. (2017). J. Am. Chem. Soc..

[cit17] Calbo J., Thawani A. R., Gibson R. S. L., White A. J. P., Fuchter M. J. (2019). Beilstein J. Org. Chem..

[cit18] Chu C.-W., Stricker L., Kirse T. M., Hayduk M., Ravoo B. J. (2019). Chem.–Eur. J..

[cit19] Golomb M., Arndt N. B., Honnigfort C., Shakhayeva B., Ravoo B. J., Braunschweig B. (2023). J. Phys. Chem. C.

[cit20] Gerkman M. A., Gibson R. S. L., Calbo J., Shi Y., Fuchter M. J., Han G. G. D. (2020). J. Am. Chem. Soc..

[cit21] Dwyer B. G., Wang C., Abegg D., Racioppo B., Qiu N., Zhao Z., Pechalrieu D., Shuster A., Hoch D. G., Adibekian A. (2021). Angew. Chem., Int. Ed..

[cit22] Sarkar H. S., Mashita T., Kowada T., Hamaguchi S., Sato T., Kasahara K., Matubayasi N., Matsui T., Mizukami S. (2023). ACS Chem. Biol..

[cit23] Stricker L., Böckmann M., Kirse T. M., Doltsinis N. L., Ravoo B. J. (2018). Chem.–Eur. J..

[cit24] Burg L., Kortekaas L., Gibalova A., Daniliuc C., Heßling J., Schönhoff M., Ravoo B. J. (2025). RSC Appl. Interfaces.

[cit25] Kortekaas L., Simke J., Kurka D. W., Ravoo B. J. (2020). ACS Appl. Mater. Interfaces.

[cit26] Meteling H. J., Bosse F., Schlichter L., Tyler B. J., Arlinghaus H. F., Ravoo B. J. (2022). Small.

[cit27] Daidone G., Raffa D., Maggio B., Plescia F., Cutuli V. M. C., Mangano N. G., Caruso A. (1999). Arch. Pharm..

[cit28] Bhatt A. H., Parekh Η. H., Pankh A. R. (1998). Heterocycl. Commun..

[cit29] Dolai A., Bhunia S., Jana S. K., Bera S., Mandal S., Samanta S. (2024). ChemBioChem.

[cit30] Li W.-T., Hwang D.-R., Chen C.-P., Shen C.-W., Huang C.-L., Chen T.-W., Lin C.-H., Chang Y.-L., Chang Y.-Y., Lo Y.-K., Tseng H.-Y., Lin C.-C., Song J.-S., Chen H.-C., Chen S.-J., Wu S., Chen C.-T. (2003). J. Med. Chem..

[cit31] Cheruthu N. M., Hashim P. K., Sahu S., Takahashi K., Nakamura T., Mitomo H., Ijiro K., Tamaoki N. (2025). Org. Biomol. Chem..

[cit32] He Y., Shangguan Z., Zhang Z.-Y., Xie M., Yu C., Li T. (2021). Angew. Chem., Int. Ed..

[cit33] Gemen J., Church J. R., Ruoko T.-P., Durandin N., Białek M. J., Weißenfels M., Feller M., Kazes M., Odaybat M., Borin V. A., Kalepu R., Diskin-Posner Y., Oron D., Fuchter M. J., Priimagi A., Schapiro I., Klajn R. (2023). Science.

[cit34] Stricker L., Fritz E.-C., Peterlechner M., Doltsinis N. L., Ravoo B. J. (2016). J. Am. Chem. Soc..

[cit35] Noyce D. S., Sandel B. B. (1976). J. Org. Chem..

[cit36] Cheng K., Qi J., Ren X., Zhang J., Li H., Xiao H., Wang R., Liu Z., Meng L., Ma N., Sun H. (2022). Angew. Chem., Int. Ed..

[cit37] Devi S., Saraswat M., Grewal S., Venkataramani S. (2018). J. Org. Chem..

[cit38] Bandara H. M. D., Burdette S. C. (2012). Chem. Soc. Rev..

[cit39] Gemen J., Białek M. J., Kazes M., Shimon L. J. W., Feller M., Semenov S. N., Diskin-Posner Y., Oron D., Klajn R. (2022). Chem.

[cit40] Gemen J., Ahrens J., Shimon L. J. W., Klajn R. (2020). J. Am. Chem. Soc..

[cit41] Seyfried M. D. Gemen J. , Wyszynski L., Giard C. L., Daniliuc C. G., Schönhoff M., Doltsinis N. L., Glorius F. and Ravoo B. J., CCDC 2463597: Experimental Crystal Structure Determination, 2025, 10.5517/ccdc.csd.cc2npkwt PMC12459379 41000131

