# Supplementary material for: Azobisheteroarene photoswitches based on isoxazoles and pyrazoles: tunable photostationary states, thermal relaxation and sensitization under confinement
Source: Chem Sci. 2025 Sep 18;16(41):19448–55. doi: 10.1039/d5sc04412j (PMC12459379; doi:10.1039/d5sc04412j)
Supplement: SC-016-D5SC04412J-s001 [file SC-016-D5SC04412J-s001.pdf]

Supplementary Information (SI) for Chemical Science.
This journal is © The Royal Society of Chemistry 2025

Azobisheteroarene photoswitches based on isoxazoles and pyrazoles: tunable photostationary states, thermal relaxation and sensitization under confinement

Maximilian Seyfried[a], Julius Gemen[a], Leonard Wyszynski[b], Carl L. Giard[c], Constantin G. Daniliuc[a], Monika Schönhoff[b, d], Nikos L. Doltsinis[c, d], Frank Glorius[a] and Bart Jan Ravoo*[a, d]

[a] Organic Chemistry Institute, University of Münster, Corrensstr. 36, 48149 Münster, Germany.

[b] Institute of Physical Chemistry, University of Münster, Corrensstr. 28/30, 48149 Münster, Germany.

[c] Institute of Solid State Theory, University of Münster, Wilhelm-Klemm-Str. 10, 48149 Münster, Germany.

[d] Center for Soft Nanoscience, University of Münster, Busso-Peus-Str. 10, 48149 Münster, Germany.

E-mail: b.j.ravoo@uni-muenster.de

Contents

Azobisheteroarene photoswitches based on isoxazoles and pyrazoles: tunable photostationary states, thermal relaxation and sensitization under confinement 1

Materials and methods 2

Synthesis and characterization 2

Determination of isomer distribution at PSS via NMR 19

Determination of thermal half-life times 25

UV/vis spectroscopy in alternative solvents 27

Sensitized isomerization of BIZs and IPZs via DESC 29

Computational details 37

Cartesian coordinates 41

Crystallographic Data 64

References 70

Materials and methods

Unless specified otherwise, all reactions were conducted without inert gas. Steps requiring inert conditions were conducted under argon atmosphere using standard SCHLENK techniques. All chemicals were purchased from Merck KGaA, BLD Pharmatech Ltd., and TCI Europe and were used without further purification. NMR spectra were recorded on a Bruker AV400 (400 MHz) an Agilent DD2 500 (500 MHz) or an Agilent DD2 600 (600 MHz). ^{1}H NMR chemical shifts are given in ppm (parts per million) relative to TMS and are referenced to the residual solvent signal. Spectra of ^{13}C are referenced according to the proton resonance of TMS as the primary reference for the unified chemical shift scale (IUPAC recommendation 2001). High-resolution mass spectrometry was performed using an Exploris 120 Electrospray Orbitrap. UV/vis absorption spectra were measured on a JASCO V-650 double-beam spectrophotometer or a JASCO V-730 spectrophotometer. All samples were irradiated from a distance of 5 cm at room temperature and without removal of oxygen. LED lamps were purchased at Roithner Lasertechnik GmbH.

Table S1: LEDs used in this work and their corresponding power.

LED	Power (minimum/typical, mW)
310 nm (DUV310-SD353EL)	33/47
365 nm (SMB1N-365V-02)	640/1300
445 nm (GD35V-445-DL)	-
515 nm (SMB1N-D520-02)	250 / 550
550 nm (SMB1N-550H-02)	-/1200

Synthesis and characterization

Effect of acidity on the azocoupling of **4iz-NH$_2$** and **3iz-NH$_2$**

General procedure: The starting material was dissolved in a mixture of acid 1, acid 2 and the solvent. The mixture was cooled in an ice bath and $NaNO_2$ dissolved a minimal amount of H_2O was added dropwise. The mixture was then stirred at 0°C for 30 min. In a separate flask, pentane-2,4-dione and NaOAc were suspended in a mixture of EtOH and H_2O (60/40 *v*/*v*). The diazonium salt was then added the the second flask and the mixture was stirred at room temperature until completion. Water was added and the aqueous layer was extracted using DCM. The crude product was then purified via column chromatography.

Table 2:tested conditions for the azocoupling of the isoxazole amines and petane-2,4-dione.

Entry	Starting material	Acid 1	Acid 2	Solvent	Base	Yield
1	**4iz-NH$_2$**	AcOH (0.8 ml/mmol)	HCl (0.12 ml/mmol)	-	NaOAc (5 eq.)	30 %
2	**4iz-NH$_2$**	AcOH (0.9 ml/mmol)	HCl (0.15 ml/mmol)	-	NaOAc (5 eq.)	65 %
3	**4iz-NH$_2$**	AcOH (0.9 ml/mmol)	HCl (0.18 ml/mmol)	-	NaOAc (5.1 eq.)	88 %
4	**3iz-NH$_2$**	AcOH (1.5 ml/mmol)	HCl (0.23 ml/mmol))	-	NaOAc (3 eq.)	< 5 %
5	**3iz-NH$_2$**	HCl (0.46 ml/mmol)	-	H_2O (2 ml/mmol)	NaOAc (5 eq.)	traces
6	**3iz-NH$_2$**	HCl (1.0 ml/mmol)	-	H_2O (2 ml/mmol)	NaOAc (5 eq.)	49 %
7	**3iz-NH$_2$**	HCl (1.0 ml/mmol)	-	H_2O (2 ml/mmol)	NaOAc (3 eq.)	traces
8	**3iz-NH$_2$**	HCl (1.0 ml/mmol)	-	H_2O (2 ml/mmol)	NaOAc (6 eq.)	54 %

For the azocoupling of **4iz-NH$_2$**, a clear trend is observed. Increasing the ratio of the stronger acid HCl leads to a significant increase in yield and a decrease in formation of undesired side products. For **3iz-NH$_2$** the mixture of AcOH and HCl (entry 4) as well as an insufficient amount of HCl in H_2O (entry 5) led to the formation of the undesired triazene as the main product. A decreased amount of base (entry 7) in the second step resulted in the formation of the diazonium salt under acidic conditions indicated by a color change to a dark orange but no formation of either the desired product or the triazene side product.

3,3'-(triaz-1-ene-1,3-diyl)bis(5-methylisoxazole) (triazene side product)

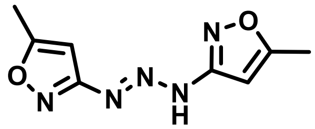

^{1}H NMR (400 MHz, CDCl$_3$): δ 6.23 (s, 2H), 2.44 (s, 3H), 2.44 (s, 3H).

MS (ESI, MeOH): *m/z* calculated for [M+Na]$^+$: 230.0648; found: 230.0645.

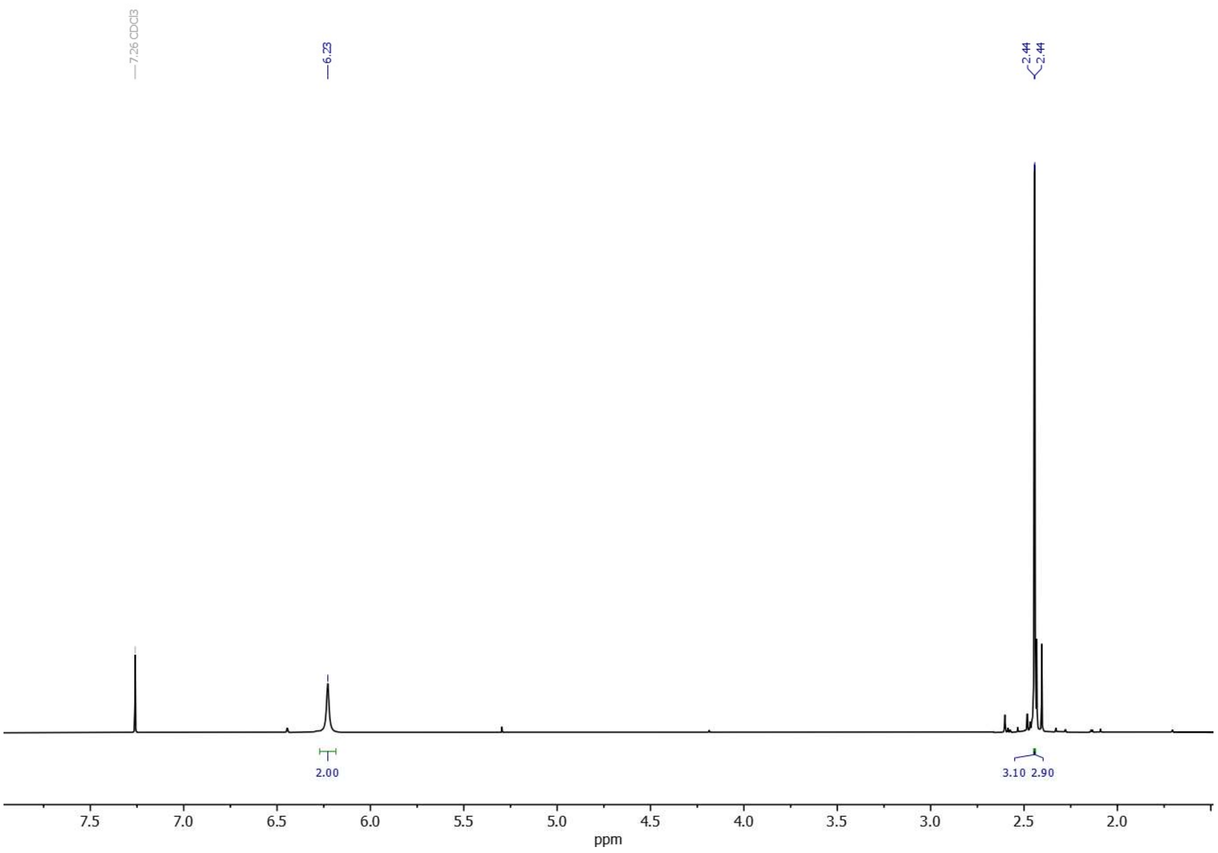

3-(2-(3,5-dimethylisoxazol-4-yl)hydrazineylidene)pentane-2,4-dione (4iz-diketon)

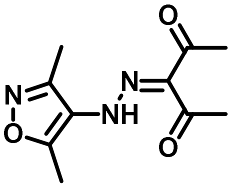

3,5-dimethylisoxazol-4-amine (200 mg, 1.78 mmol, 1.00 eq.) was dissolved in AcOH (1.5 ml) and conc. HCl (0.3 ml) and the solution was cooled in an ice bath. Then sodium nitrite (147 mg, 2.13 mmol, 1.19 eq.) dissolved in H_2O (0.5 mL) was added dropwise to the solution. The yellow solution was stirred at 0 °C for 20 min. In a separate flask, pentane-2,4-dione (235 mg, 240 µL, 2.35 mmol, 1.32 eq.) and NaOAc (748 mg, 9.12 mmol, 5.11 eq.) were suspended in EtOH (2.4 ml) and H_2O (1.6 ml).

The orange diazonium salt was transferred to the petane-2,4-dione solution and the mixture was stirred at room temperature for 2 h. Dest. water (30 mL) was added, and the aq. phase was extracted using DCM (3x25 ml). The combined organic layer was dried over $MgSO_4$, and the solvent was evaporated to obtain the crude product as a red solid. The product was used without further purification.

Yield: 350 mg, 1.57 mmol, 88%.

R_f: 0.35 (DCM/MeOH, 99:1, *v*/*v*).

^{1}H NMR (400 MHz, $CDCl_3$): δ 2.60 (s, 1H), 2.54 (s, 1H), 2.40 (s, 1H), 2.38 (s, 1H). (Additional acidic proton at 14.27 ppm might be observed).

^{13}C NMR (101 MHz, $CDCl_3$): δ 198.42, 196.54, 158.52, 153.53, 134.58, 120.36, 31.69, 26.94, 11.73, 10.55.

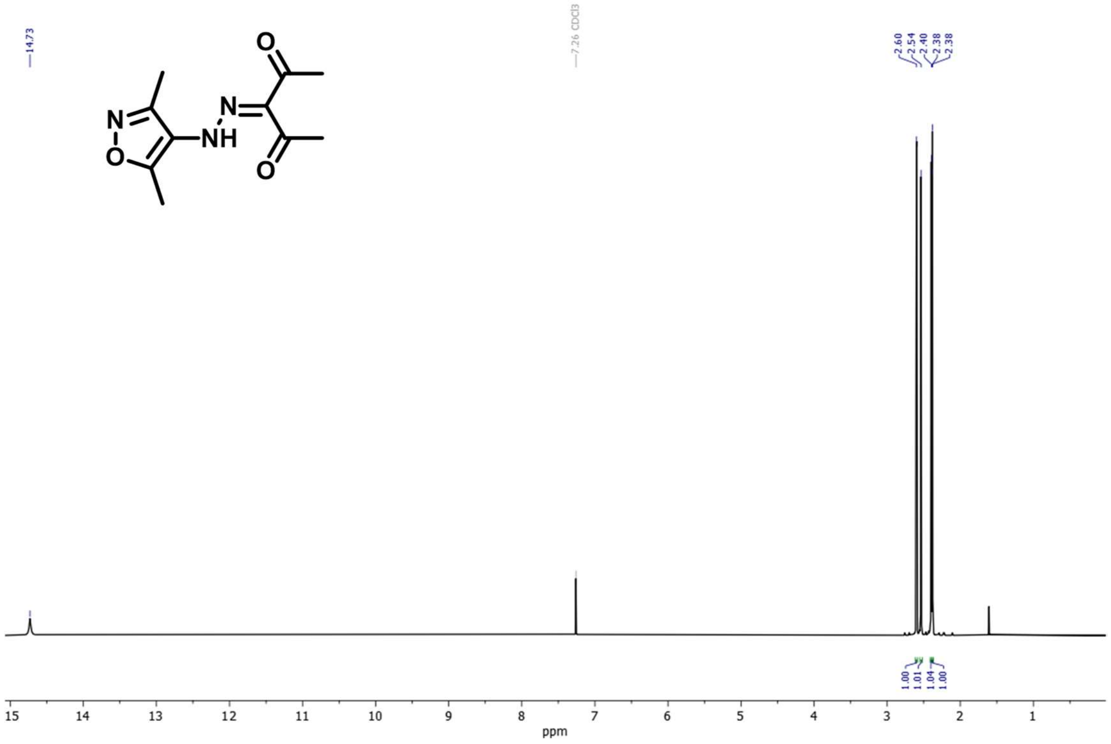

Figure S1: ^{1}H NMR of 4iz-diketon ($CDCl_3$, 400 MHz, 298 K).

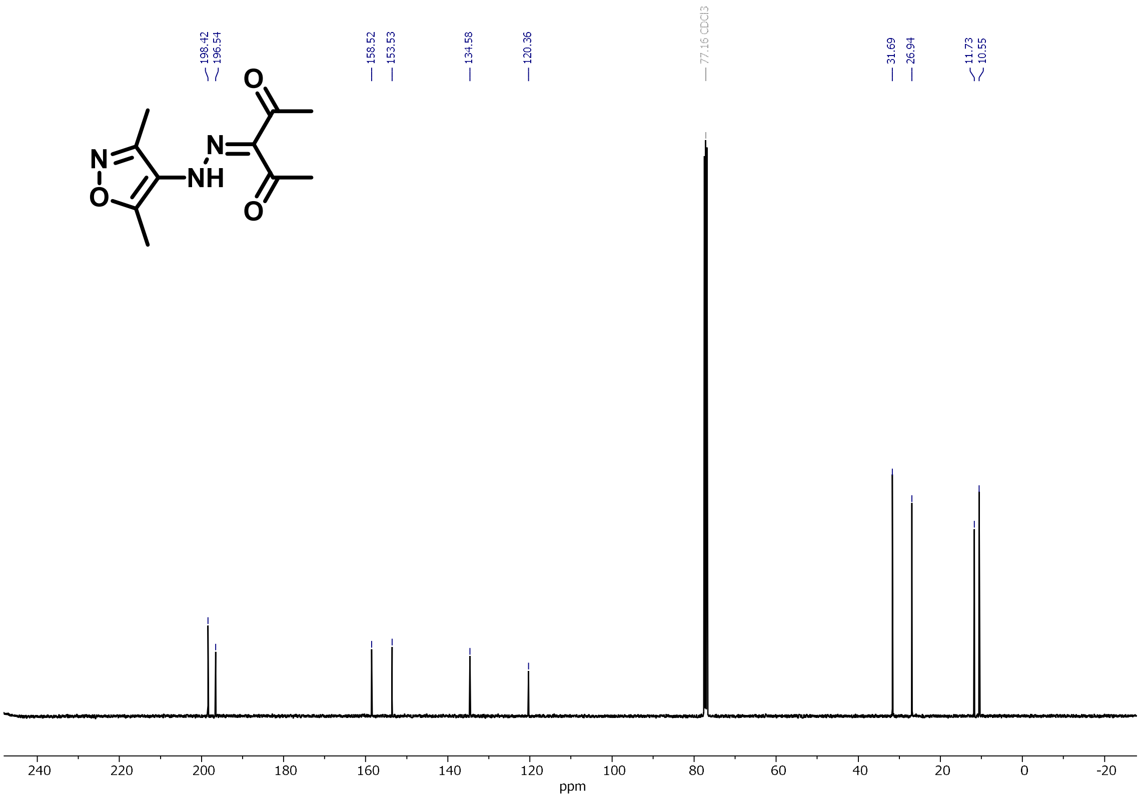

Figure S2: ^{13}C NMR of 4iz-diketon ($CDCl_3$, 400 MHz, 298 K).

3-(2-(5-methylisoxazol-3-yl)hydrazineylidene)pentane-2,4-dione (3iz-diketon)

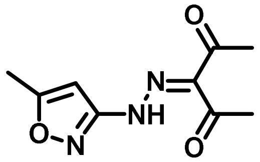

5-methylisoxazol-3-amine (196 mg, 1.99 mmol, 1.00 eq.) was dissolved in a mixture of dest. H_2O (4 ml) and conc. HCl (2 ml) and cooled in an ice bath. Then $NaNO_2$ (165 mg, 2.39 mmol, 1.20 eq.) dissolved dest. H_2O (2 ml) was added dropwise to the solution.

In a second flask, NaOAc (1.07 g, 13.0 mmol, 6.55 eq.) and pentane-2,4-dione (261 mg, 266 µl, 2.60 mmol, 1.31 eq.) were suspended in a mixture of EtOH (1.6 ml) and dest. H_2O (1.3 ml). After 30 min of stirring, the pale yellow diazonium salt suspension was added to the EtOH/H_2O solution and stirred for 30 min at 0 °C. The ice bath was removed, and the mixture was stirred overnight. Water was added and the aq. phase was extracted with DCM (2x20 ml). The combined organic phase was dried over $MgSO_4$ and filtered. Then silica was added, and the solvent was removed under reduced pressure.

The adsorbed product was then purified via column chromatography (DCM/MeOH, 99:1, *v*/*v*). The product was obtained as a pale yellow solid.

Yield: 225 mg, 1.08 mmol, 54%.

^{1}H NMR (400 MHz, $CDCl_3$): δ 14.19 (s, 1H), 6.25 (m, 1M), 2.58 (s, 3H), 2.42 (s, 6H).

^{13}C NMR (101 MHz, $CDCl_3$): δ 198.03, 196.70, 170.92, 163.40, 135.16, 93.16, 31.70, 26.37, 12.70.

MS (ESI, MeOH): *m*/*z* calculated for $[M+Na]^+$: 232.0693; found: 232.0692.

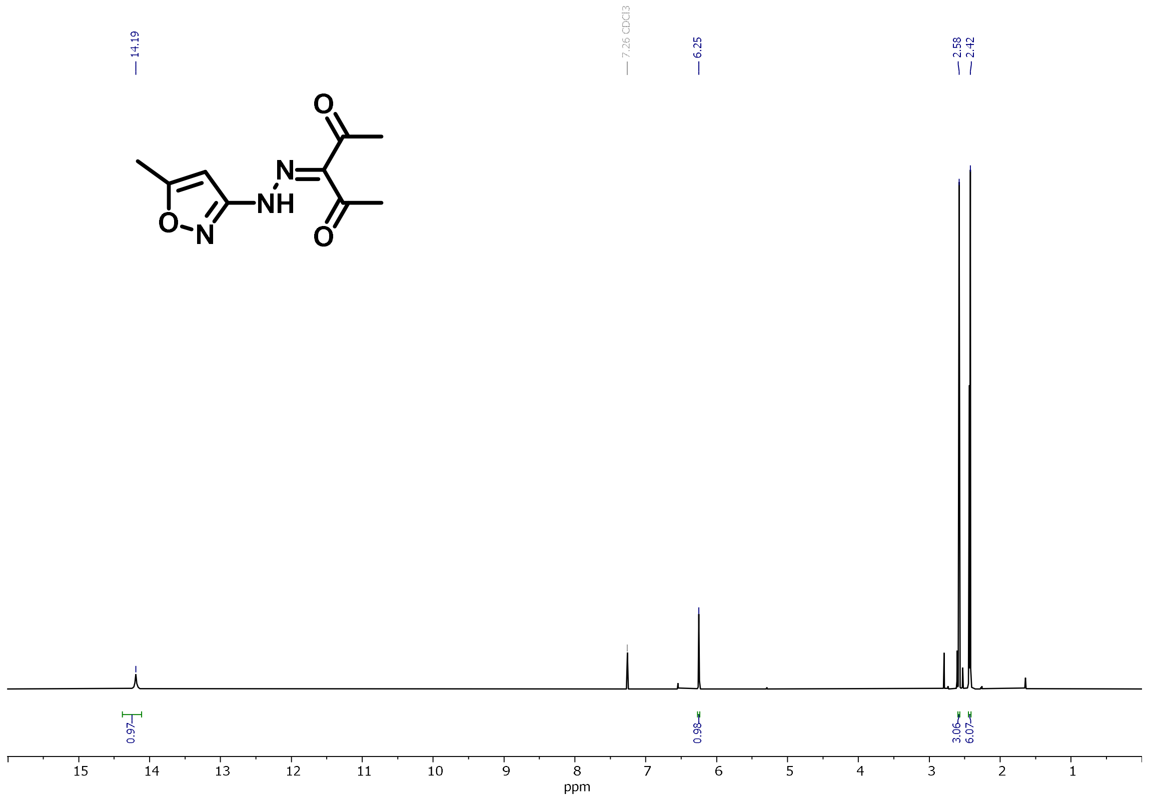

Figure S3: ^{1}H NMR of 3iz-diketon ($CDCl_3$, 400 MHz, 298 K).

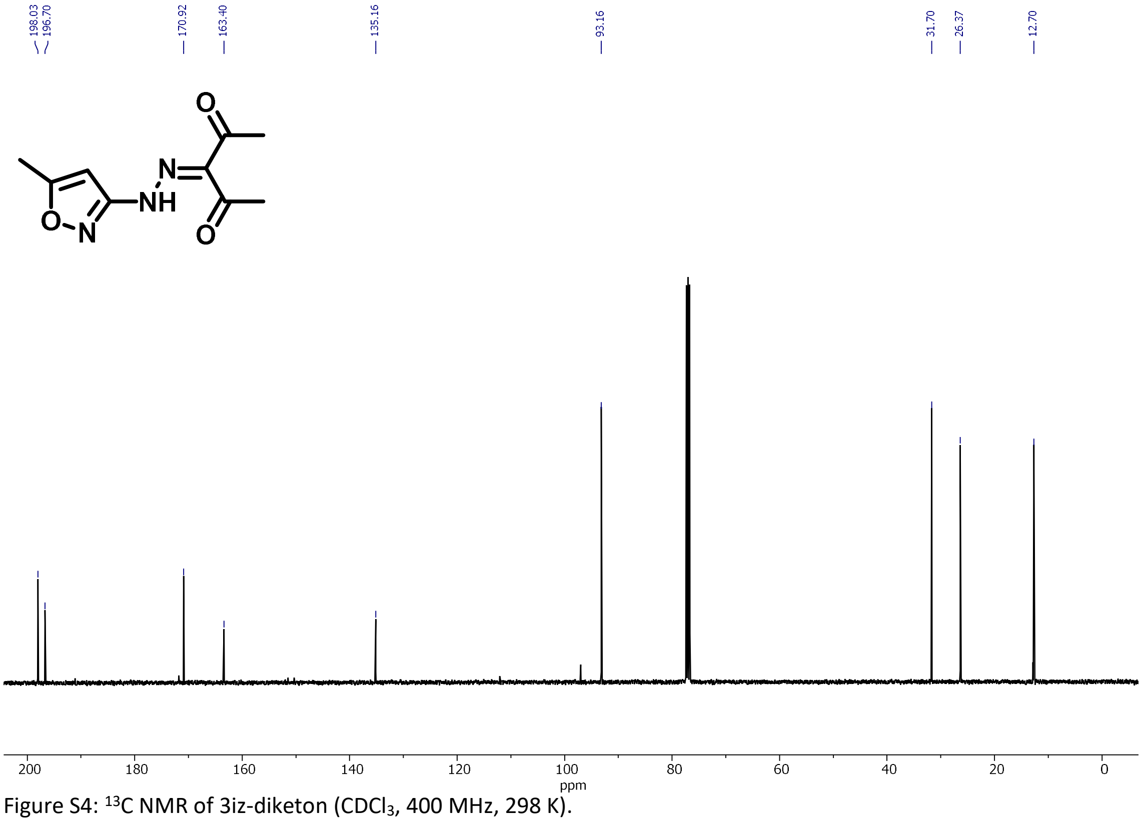

Figure S4: ^{13}C NMR of 3iz-diketon ($CDCl_3$, 400 MHz, 298 K).

(*E*)-1,2-bis(3,5-dimethylisoxazol-4-yl)diazene (**4iz-4iz**)

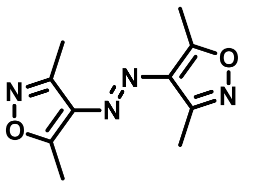

4iz-diketon (33 mg, 0.15 mmol, 1.0 eq.), hydroxylamine hydrochloride (16 mg, 0.23 mmol, 1.6 eq.) and Na_2CO_3 (26 mg, 0.25 mmol,1.7 eq.) were dissolved in EtOH (4 ml) and refluxed for 72 h. The resulting solution was directly adsorbed onto silica and the crude product was purified via column chromatography (silica, DCM/MeOH, 200:1, *v*/*v*). The pure product was obtained as a yellow solid.

Yield: 28 mg, 0.13 mmol, 86 %.

^{1}H NMR (400 MHz, $CDCl_3$): δ 2.68 (s, 1H), 2.47 (s, 1H). (Signals of residual *Z* isomer at 2.05 and 2.14 ppm).

^{13}C NMR (101 MHz, $CDCl_3$): δ 168.54, 153.47, 132.95, 12.25, 11.75.

MS (ESI+, MeOH): *m*/*z* calculated for $[M+H]^+$: 221.1033; found: 221.1033.

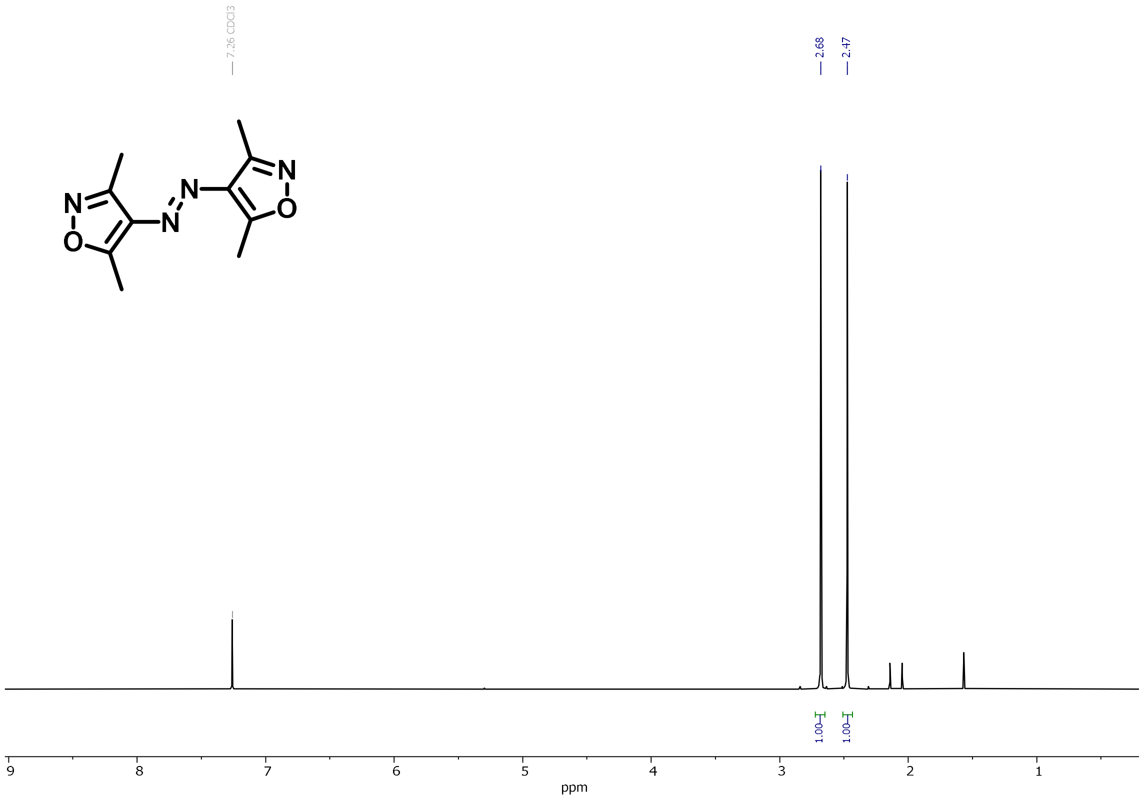

Figure S5: ^{1}H NMR of **4iz-4iz** ($CDCl_3$, 400 MHz, 298 K).

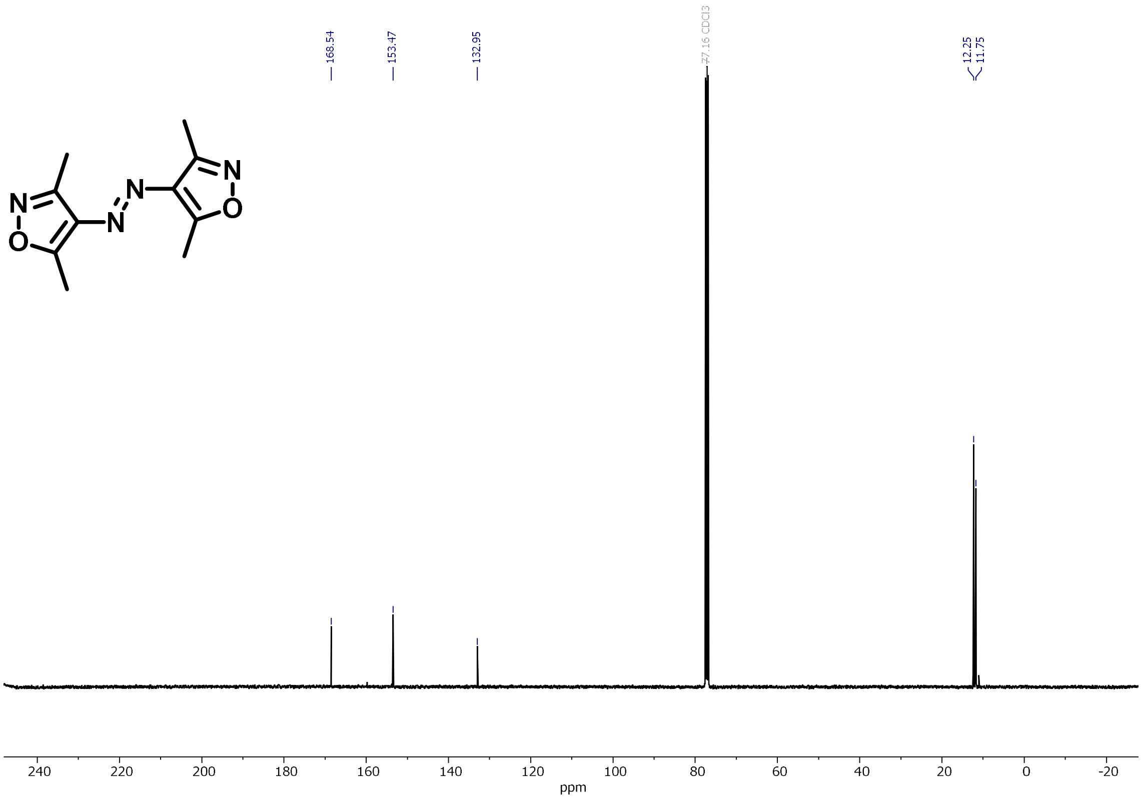

Figure S6: ^{13}C NMR of **4iz-4iz** ($CDCl_3$, 400 MHz, 298 K).

<u>1,2-bis(3,5-dimethylisoxazol-4-yl)diazene (**3iz-4iz**)</u>

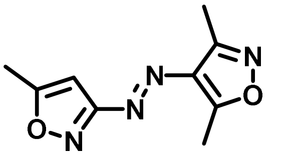

3iz-diketon (97.6 mg, 0.47 mmol, 1.0 eq.) was dissolved in EtOH (15 ml). Na_2CO_3 (69.1 mg, 0.652 mmol, 1.4 eq.) and hydroxylamine hydrochloride (49.9 mg, 0.728 mmol, 1.5 eq.) were added and the mixture was refluxed for 3 d. The yellow suspension was filtered, and the residue was discarded. The solvent was evaporated, and the crude product was purified via column chromatography (silica, DCM) to obtain the clean product as a yellow solid.

Yield: 62 mg, 0.30 mmol, 64 %.

^{1}H NMR (400 MHz, $CDCl_3$): δ 6.27 (d, *J* = 1.0 Hz, 1H), 2.74 (s, 3H), 2.52 (s, 3H), 2.48 (s, 3H).

^{13}C NMR (101 MHz, $CDCl_3$): δ 174.21, 172.02, 171.25, 153.57, 133.27, 91.89, 12.90, 12.17, 11.95.

MS (ESI+, MeOH): *m/z* calculated for $[M+Na]^+$: 229.0696; found: 229.0695.

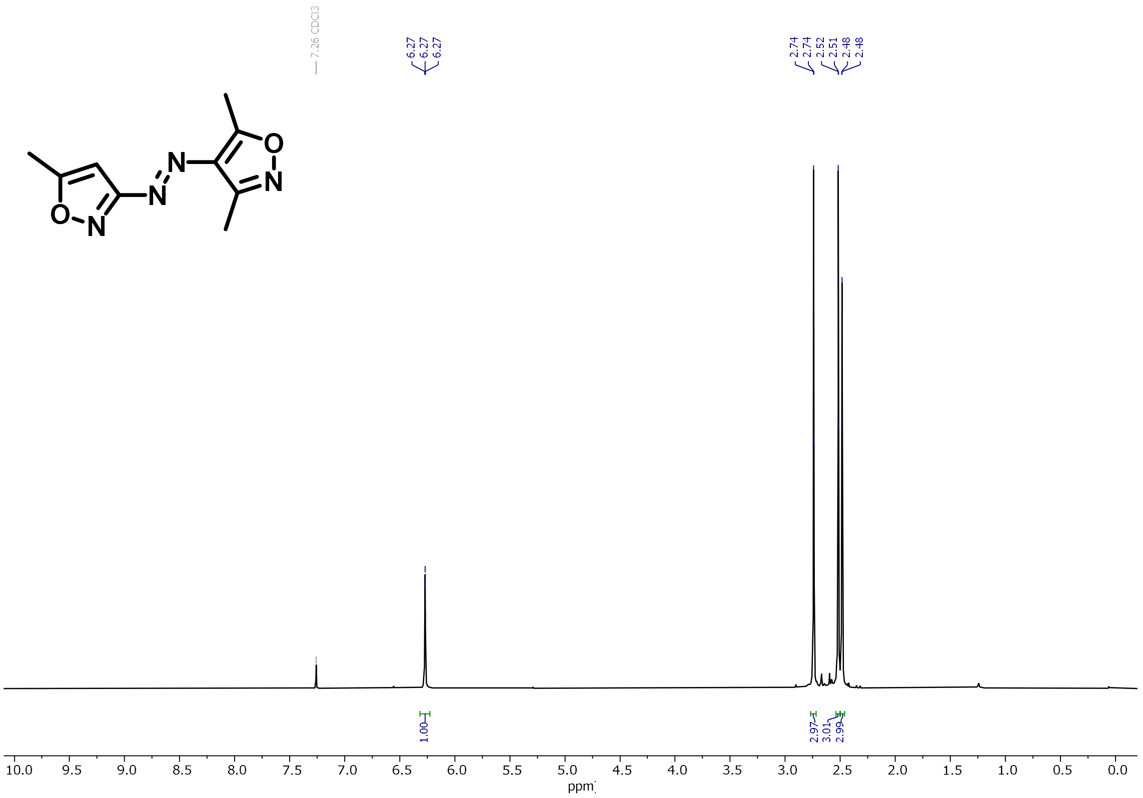

Figure S7: ^{1}H NMR of **3iz-4iz** ($CDCl_3$, 400 MHz, 298 K).

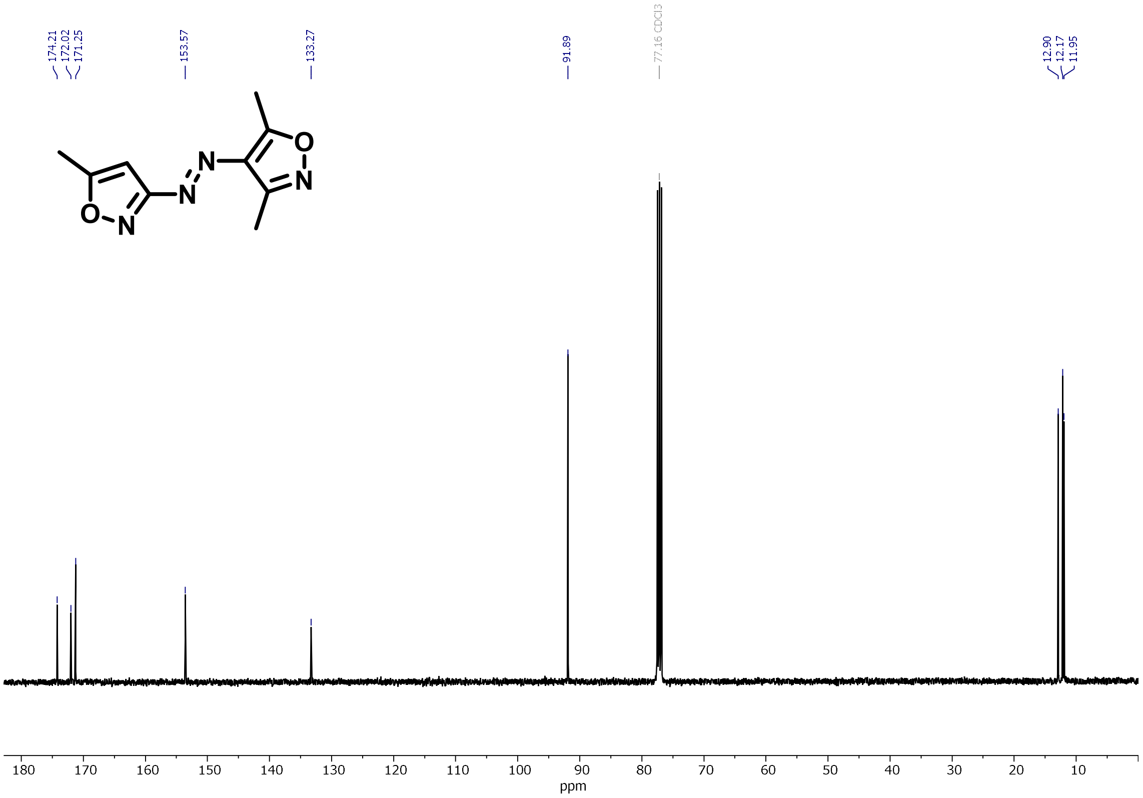

Figure S8: ^{13}C NMR of **3iz-4iz** ($CDCl_3$, 101 MHz, 298 K).

(*E*)-1,2-bis(3,5-dimethyl-1H-pyrazol-4-yl)diazene (**4iz-pzH**)

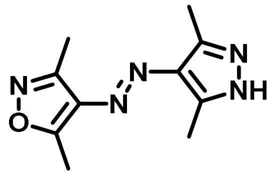

4iz-diketon (55.0 mg, 0.246 mmol, 1.0 eq.) was dissolved in EtOH (5 mL). Hydrazine hydrate (12.4 mg, 12 µL, 0.247 mmol, 1.0 eq.) was added and the mixture was refluxed overnight. The solvent was removed under reduced pressure and the crude product was purified via column chromatography (silica, DCM/MeOH (99:1 → 98:2, v/v).

Yield: 32.0 mg, 0.156 mmol, 59%.

^{1}H NMR (500 MHz, $CDCl_3$): δ 2.66 (s, 1H), 2.51 (s, 2H), 2.49 (s, 1H).

^{13}C NMR (126 MHz, $CDCl_3$): δ 166.36, 154.03, 141.08, 135.46, 133.05, 12.20, 12.01, 11.68.

MS (ESI+, MeOH): *m/z* calculated for $[M+Na]^+$: 242.1012; found: 242.1012.

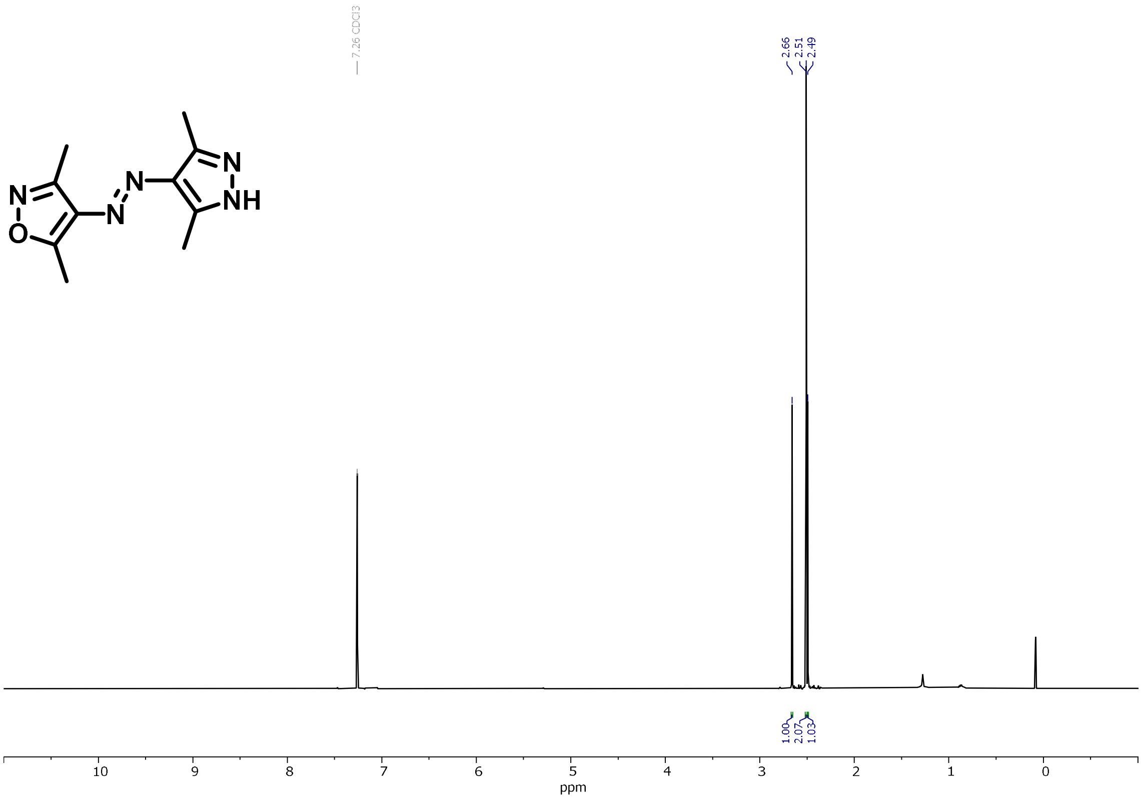

Figure S9: ^{1}H NMR of **4iz-pzH** ($CDCl_3$, 500 MHz, 328 K).

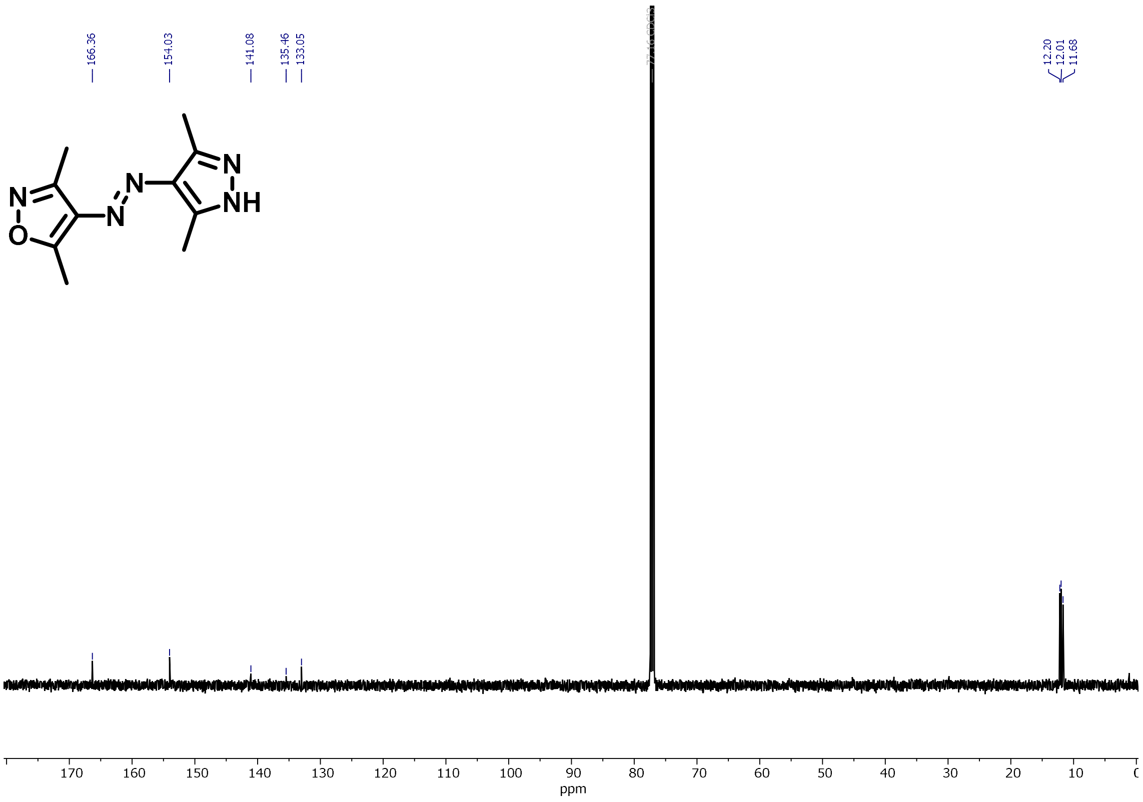

Figure S10: ^{13}C NMR of **4iz-pzH** ($CDCl_3$, 126 MHz, 328 K).

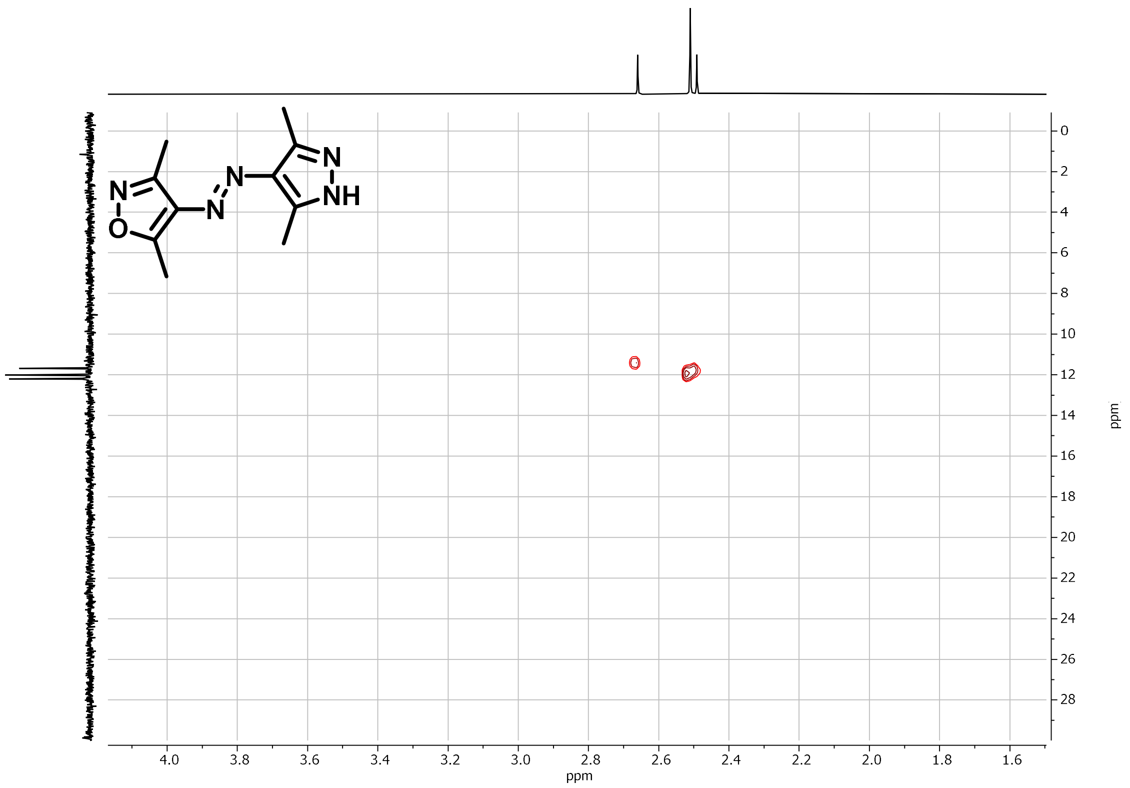

Figure S11: gHSQC of **4iz-pzH** focusing on the aliphatic region ($CDCl_3$, 500 MHz, 328 K).

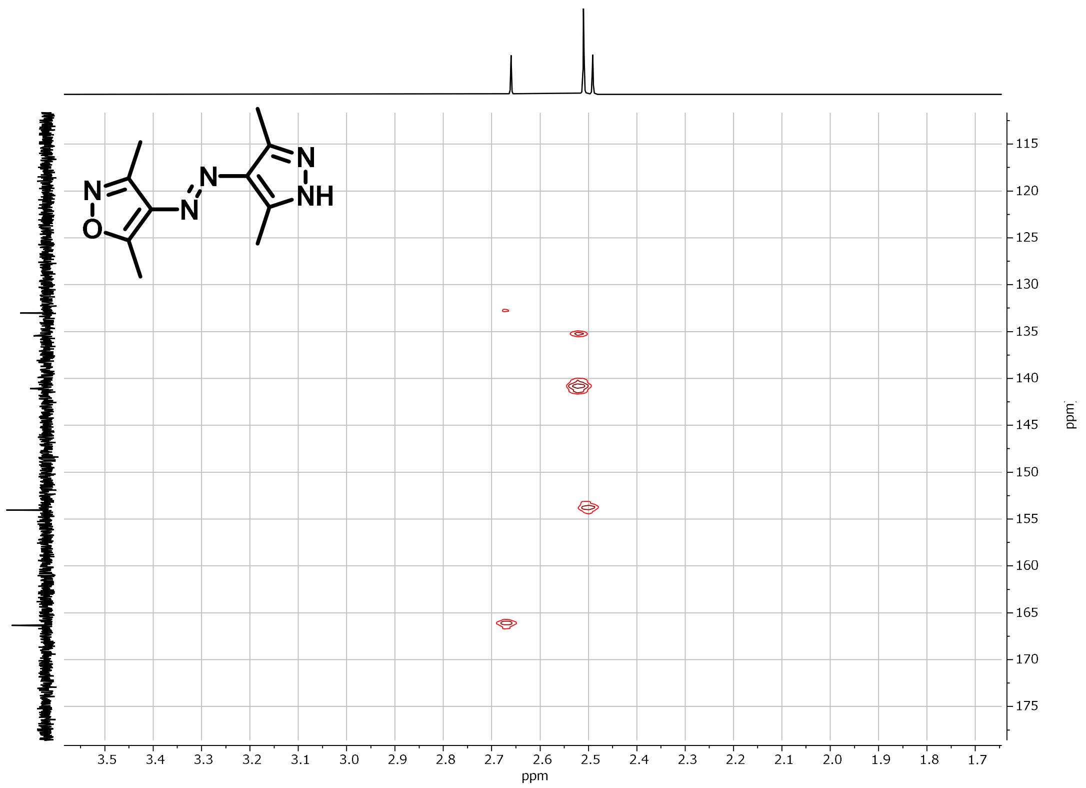

Figure S12: gHMBC of **4iz-pzH** ($CDCl_3$, 500 MHz, 328 K).

(*E*)-3-((3,5-dimethyl-1H-pyrazol-4-yl)diazenyl)-5-methylisoxazole (**3iz-pzH**)

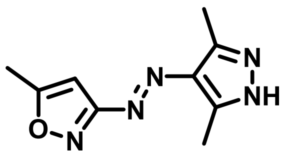

3iz-diketon (89.1 mg, 0.426 mmol, 1.0 eq.) was dissolved in EtOH (15 ml). Hydrazine hydrate (21.6 mg, 21 µL, 0.431 mmol, 1.0 eq.) was added and the mixture was refluxed for 3 d. The solvent was removed under reduced pressure, and the crude product was purified via column chromatography (silica, DCM/MeOH, 98:2, *v*/*v*).

Yield: 75 mg, 0.37 mmol, 86 %.

^{1}H NMR (400 MHz, $CDCl_3$): δ 6.29 (q, *J* = 0.9 Hz, 1H), 2.58 (s, 6H), 2.47 (d, *J* = 1.0 Hz, 3H).

^{13}C NMR (101 MHz, $CDCl_3$): δ 174.75, 170.65, 142.99, 135.45, 92.15, 12.91, 12.37.

MS (ESI+, MeOH): *m*/*z* calculated for $[M+Na]^+$: 228.0856; found: 228.0855.

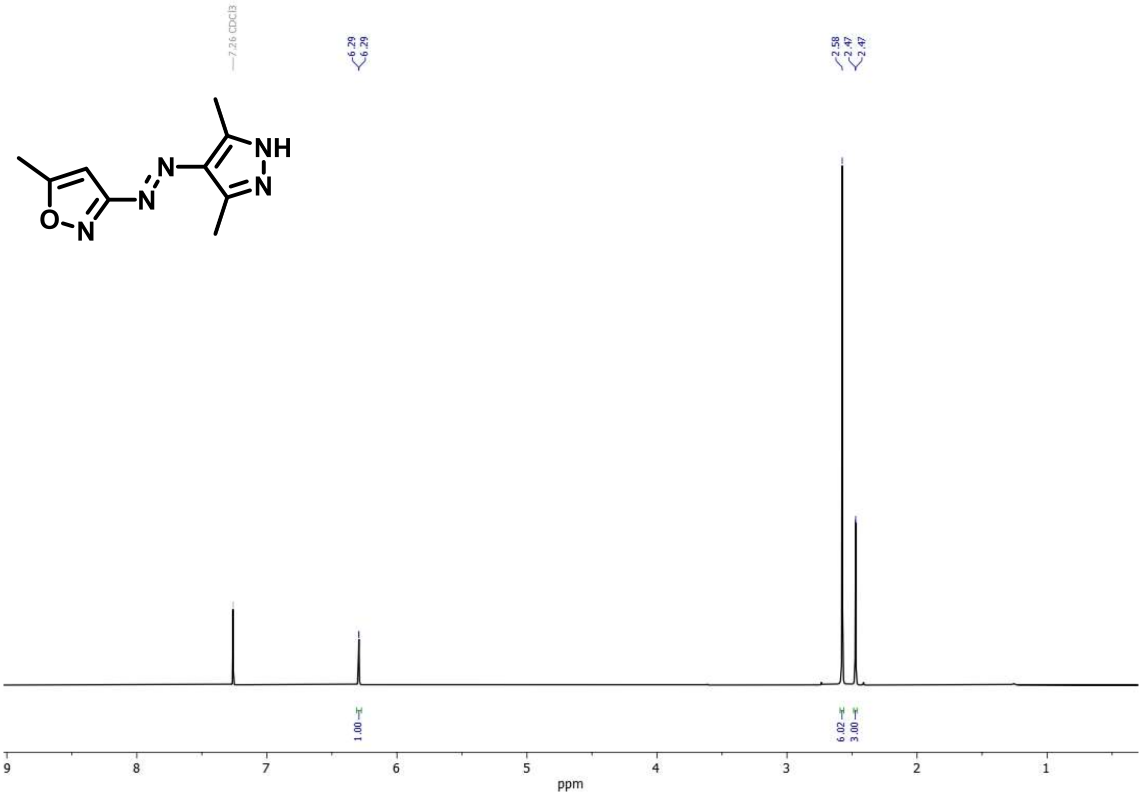

Figure S13: ^{1}H NMR of **3iz-pzH** ($CDCl_3$, 400 MHz, 298 K).

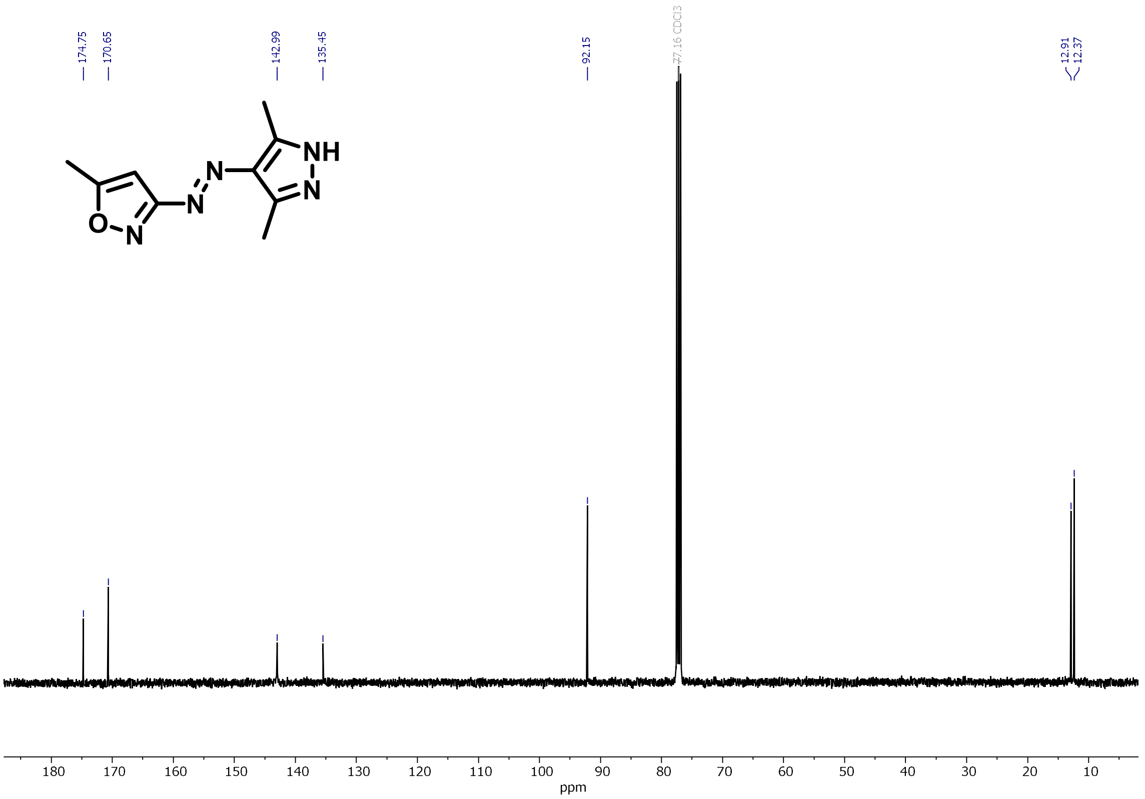

Figure S14: ^{13}C-NMR of **3iz-pzH** ($CDCl_3$, 101 MHz, 298 K).

(*E*)-4-((3,5-dimethyl-1H-pyrazol-4-yl)diazenyl)-1,3,5-trimethyl-1H-pyrazole (4iz-pzMe)

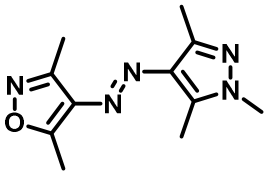

4iz-diketon (31.0 mg, 139 µmol, 1.0 eq.) was dissolved in EtOH (5 ml) and methylhydrazine (6.40 mg, 7.27 µL,139 µmol, 1.00 eq.) was added to the solution. The mixture was stirred for 24 h at room temperature. The crude product was the purified via column chromatography (silica, DCM/MeOH, 199:1, *v*/*v*).

Yield: 30.0 mg, 0.129 mmol, 93%.

R_f: 0.33 (DCM/MeOH, 99:1, *v*/*v*).

^{1}H NMR (400 MHz, $CDCl_3$): δ 3.77 (s, 3H), 2.64 (s, 3H), 2.49 (s, 3H), 2.47 (s, 3H), 2.42 (s, 3H).

^{13}C NMR (101 MHz, $CDCl_3$): δ 166.26, 153.88, 141.70, 138.31, 135.63, 132.82, 36.12, 14.06, 12.26, 11.66, 9.96.

MS (ESI+, MeOH): *m*/*z* calculated for $[M+Na]^+$: 256.1169; found: 256.1169.

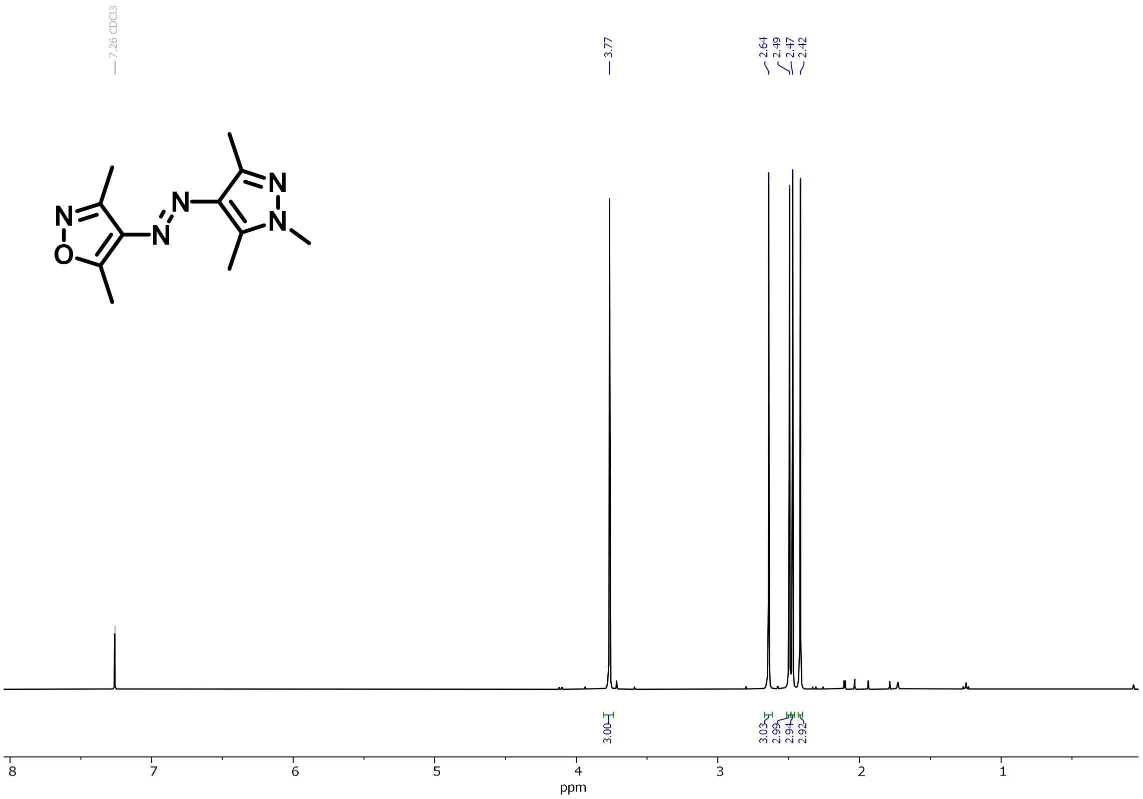

Figure S15: ^{1}H NMR of **4iz-pzMe** ($CDCl_3$, 400 MHz, 298 K).

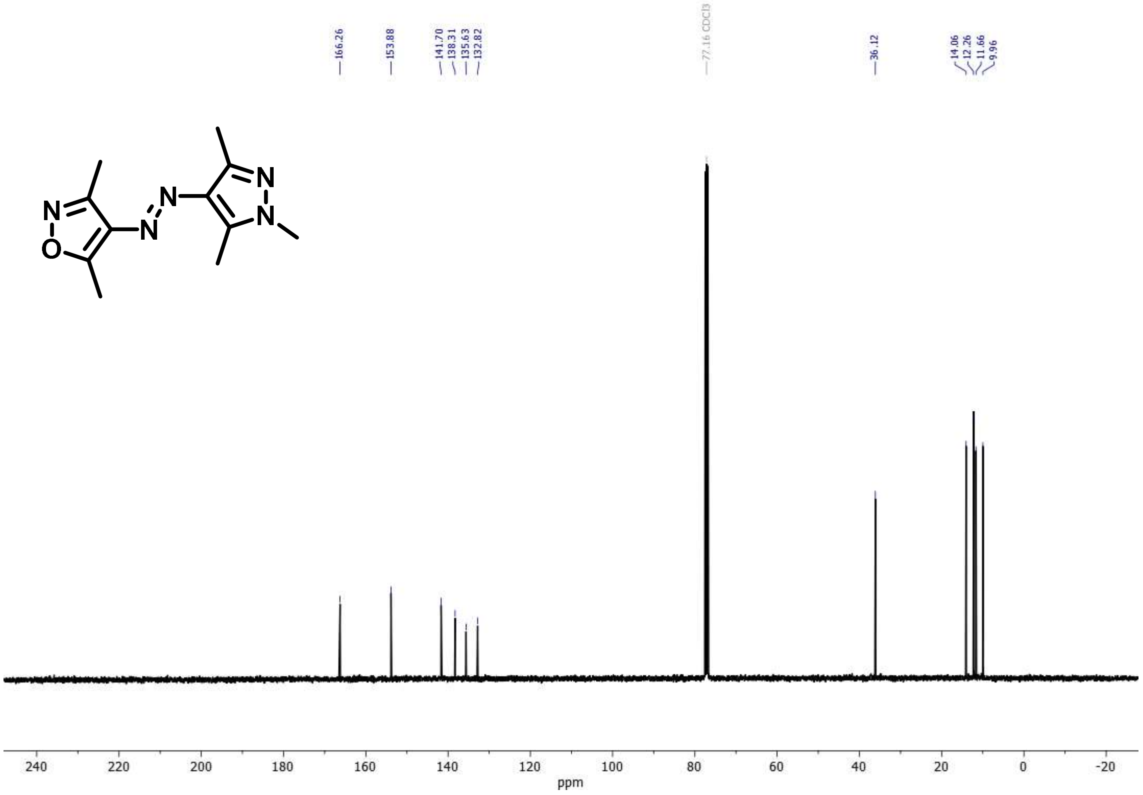

Figure S16: ^{13}C NMR of **4iz-pzMe** ($CDCl_3$, 400 MHz, 298 K).

(*E*)-5-methyl-3-((1,3,5-trimethyl-1H-pyrazol-4-yl)diazenyl)isoxazole (**3iz-pzMe**)

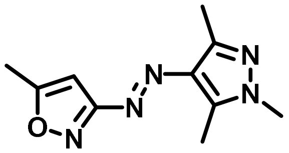

3iz-diketon (33.3 mg, 159 µmol, 1.00 eq.) was dissolved in EtOH (5 ml). Methylhydrazine (7.33 mg, 8.33 µL, 159 µmol, 1.00eq.) was added and the mixture was stirred overnight at room temperature. The solvent was removed under reduced pressure and the crude product was purified via column chromatography (DCM/MeOH, 99:1, *v*/*v*).

Yield: 32.0 mg, 1.46 mmol, 92%.

R_f = 0.36 (DCM/MeOH, 99:1, *v*/*v*).

^{1}H NMR (400 MHz, $CDCl_3$): δ 6.26 (q, *J* = 0.9 Hz, 1H), 3.76 (s, 3H), 2.54 (s, 3H), 2.46 (s, 3H), 2.44 (d, *J* = 1.0 Hz, 3H).

^{13}C NMR (101 MHz, $CDCl_3$): δ 174.83, 170.43, 143.84, 140.21, 135.78, 92.11, 36.20, 13.83, 12.85, 10.26.

MS (ESI+, MeOH): *m*/*z* calculated for $[M+Na]^+$: 242.1012; found: 242.1011.

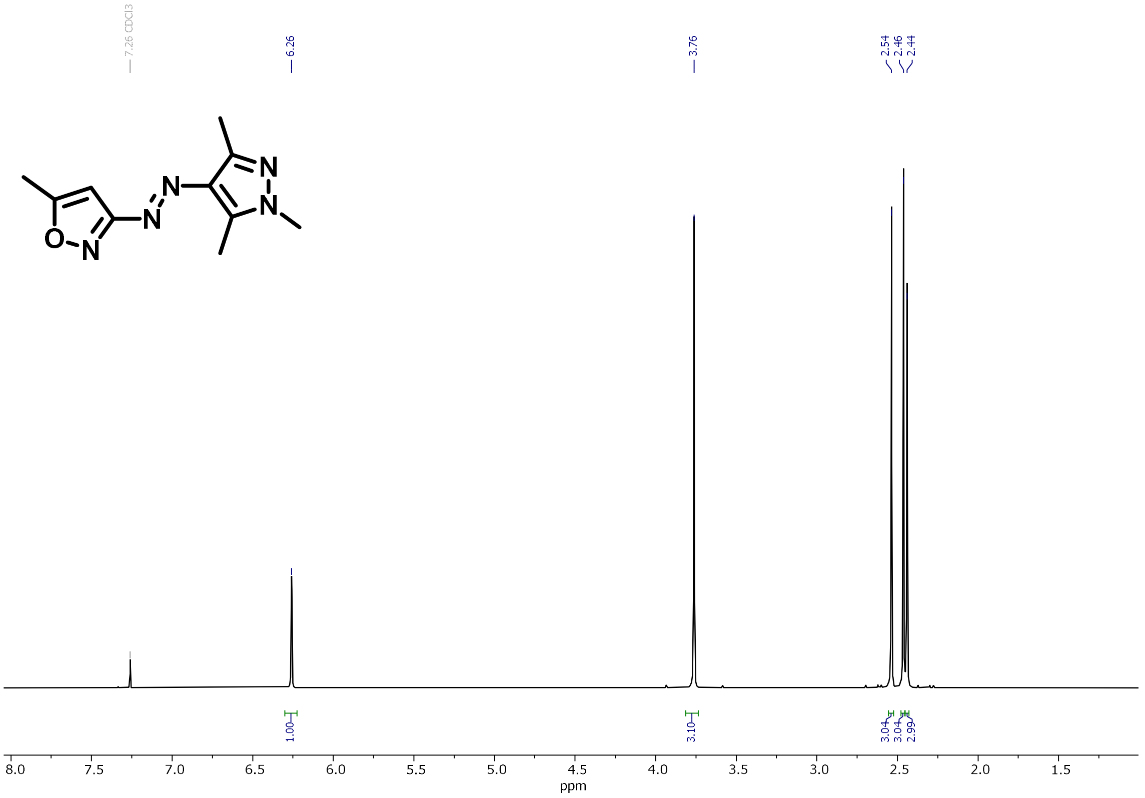

Figure S17: ^{1}H NMR of **3iz-pzMe** ($CDCl_3$, 400 MHz, 298 K).

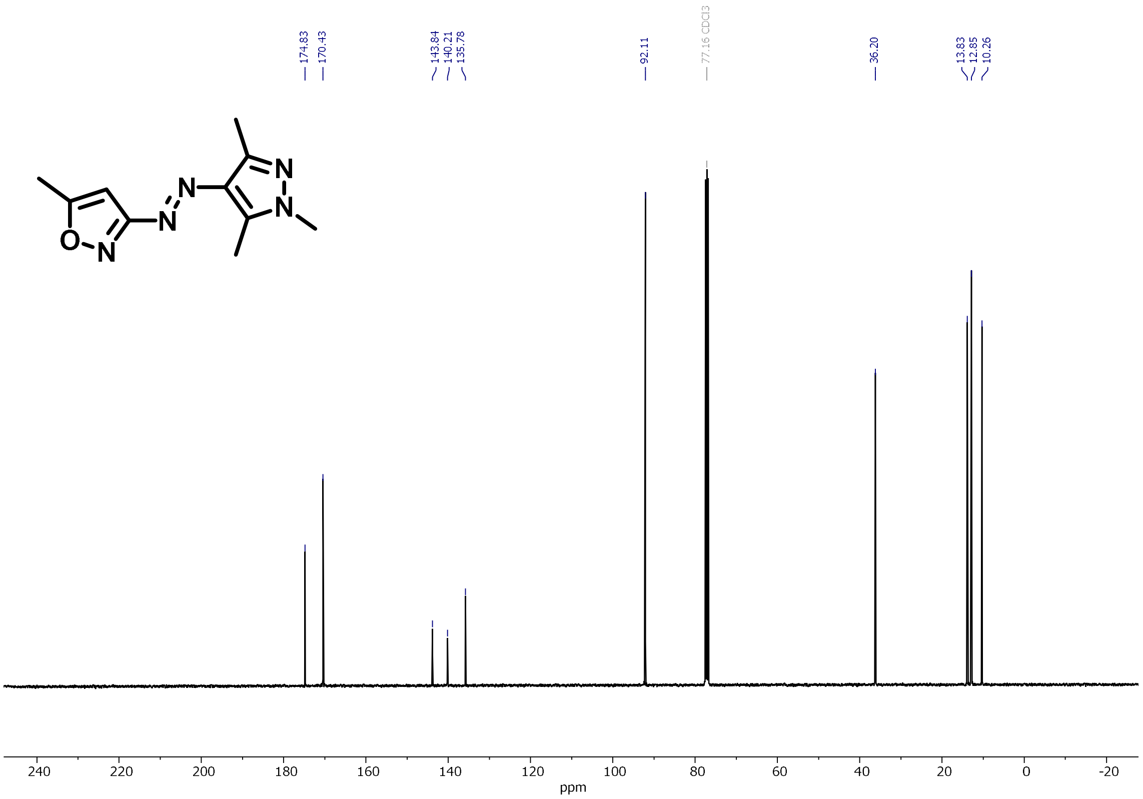

Figure S18: ^{13}C NMR of **3iz-pzMe** ($CDCl_3$, 101 MHz, 298 K).

Host **H**

Host **H** was synthesized according to a previously reported literature procedure[1].

^{1}H NMR (400 MHz, D_2O): δ = 9.12 (s, 8H), 8.82 (s, 4H), 7.75 (s, 4H), 7.72 (s, 4H), 7.70 (s, 8H), 7.65 (s, 8H), 7.55 (s, 12H), 3.16–3.07 (s, 24H), 2.83–2.68 (m, 72H).

Photosensitizer **G** (1,3,5,7,8-pentamethyl-4,4-difluoro-4-bora-3a,4a-diaza-s-indacene)

Photosensitizer **G** was synthesized according to a previously reported literature procedure[2].

^{1}H NMR (400 MHz, $CDCl_3$): δ 6.05 (s, 2H), 2.58 (s, 3H), 2.52 (s, 6H), 2.41 (s, 6H).

Determination of isomer distribution at PSS via NMR

For samples with $t_{1/2}$ over 2 h, samples (5 mg/mL) were irradiated for 20 min in an NMR tube and measured immediately. ^{1}H-NMR spectra were recorded on an Agilent DD2 600 spectrometer at 600 MHz. The photostationary state (PSS) distribution was determined by integrating peak areas of *E* and *Z* isomers. For Samples with short $t_{1/2}$ measurements of the initial state were recorded on an Agilent DD2 600 spectrometer at 600 MHz, NMR measurements of the irradiated samples were conducted on a Bruker Avance III HD 400 MHz NMR spectrometer equipped with a gradient probe head with a selective ^{1}H insert ("Diff50", Bruker). The samples were measured at 25 °C after calibration in a reference tube containing a PT100 thermocouple in oil. For in situ NMR experiments with light

irradiation by LEDs, 5 mm precision NMR tubes were filled with 300 µL of the sample solution (5 mM), followed by insertion of the end of an optical fiber waveguide into the NMR tubes. Samples were continuously irradiated with UV or green (365 nm, 20.8 mW/cm^2; 515 nm) light during the measurement. To minimize the effect of residual HCl in $CDCl_3$ which can drastically reduce thermal stability of the *Z* isomer $CDCl_3$ was deacidified by the addition of a small amount of K_2CO_3 before use.[3]

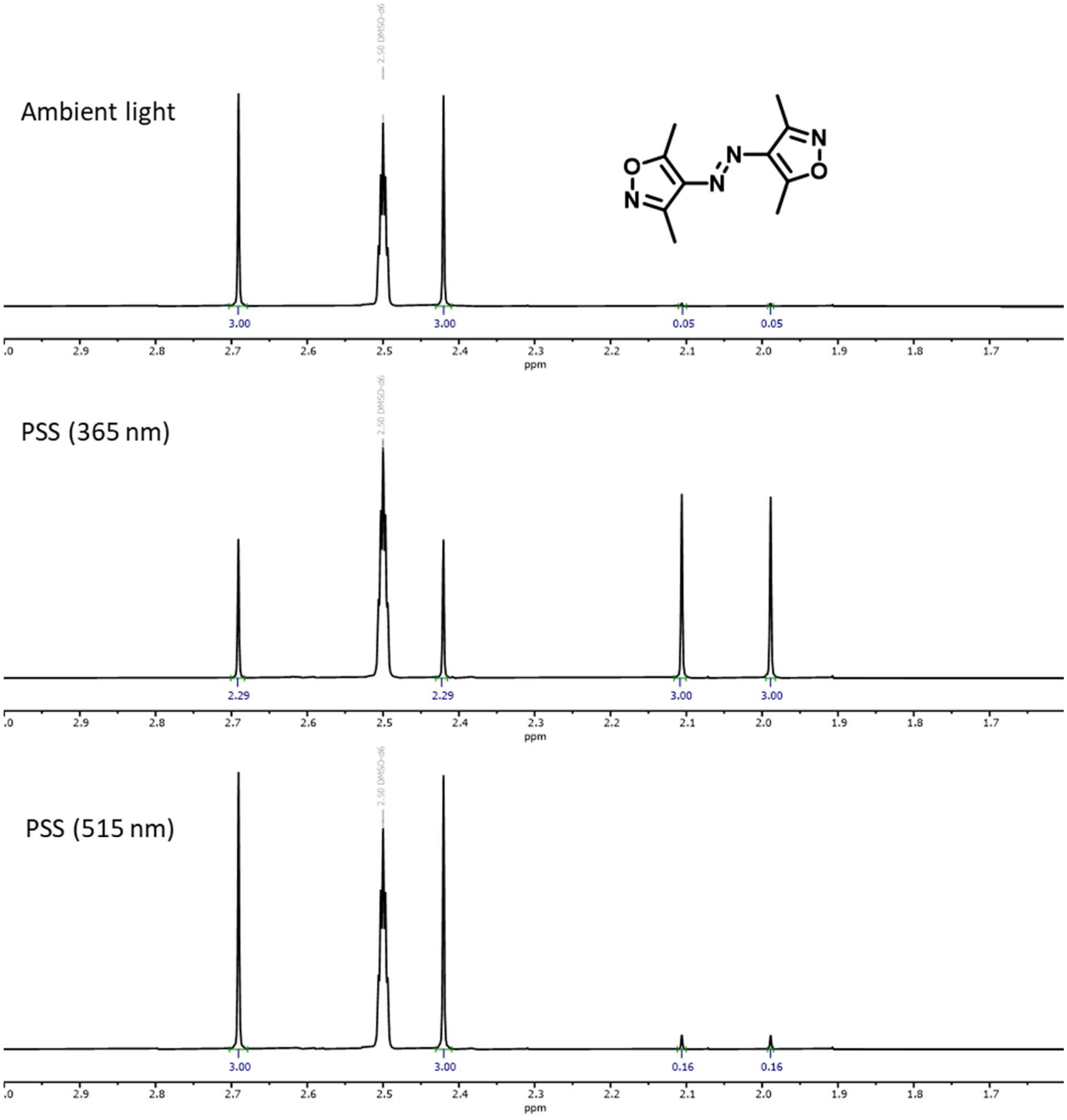

Figure S19: ^{1}H NMR of **4iz-4iz** (5 mg/mL, DMSO-d$_6$, 600 MHz, 298 K), before irradiation and after irradiation with 365 nm or 515 nm. Irradiation time: 20 min.

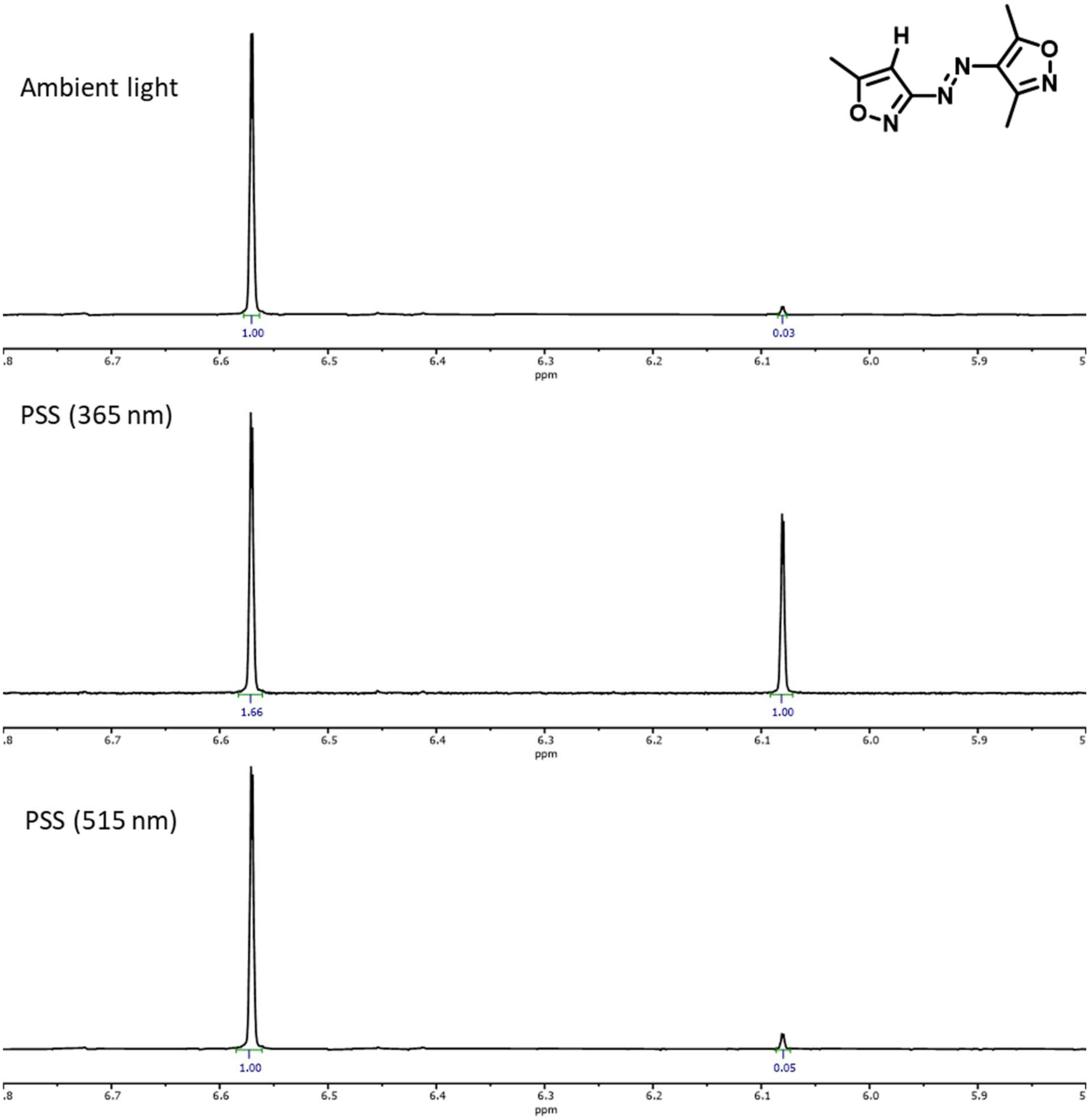

Figure S20: ^{1}H NMR of **3iz-4iz** (5 mg/mL, DMSO-d_6, 600 MHz, 298 K), before irradiation and after irradiation with 365 nm or 515 nm. Irradiation time: 20 min.

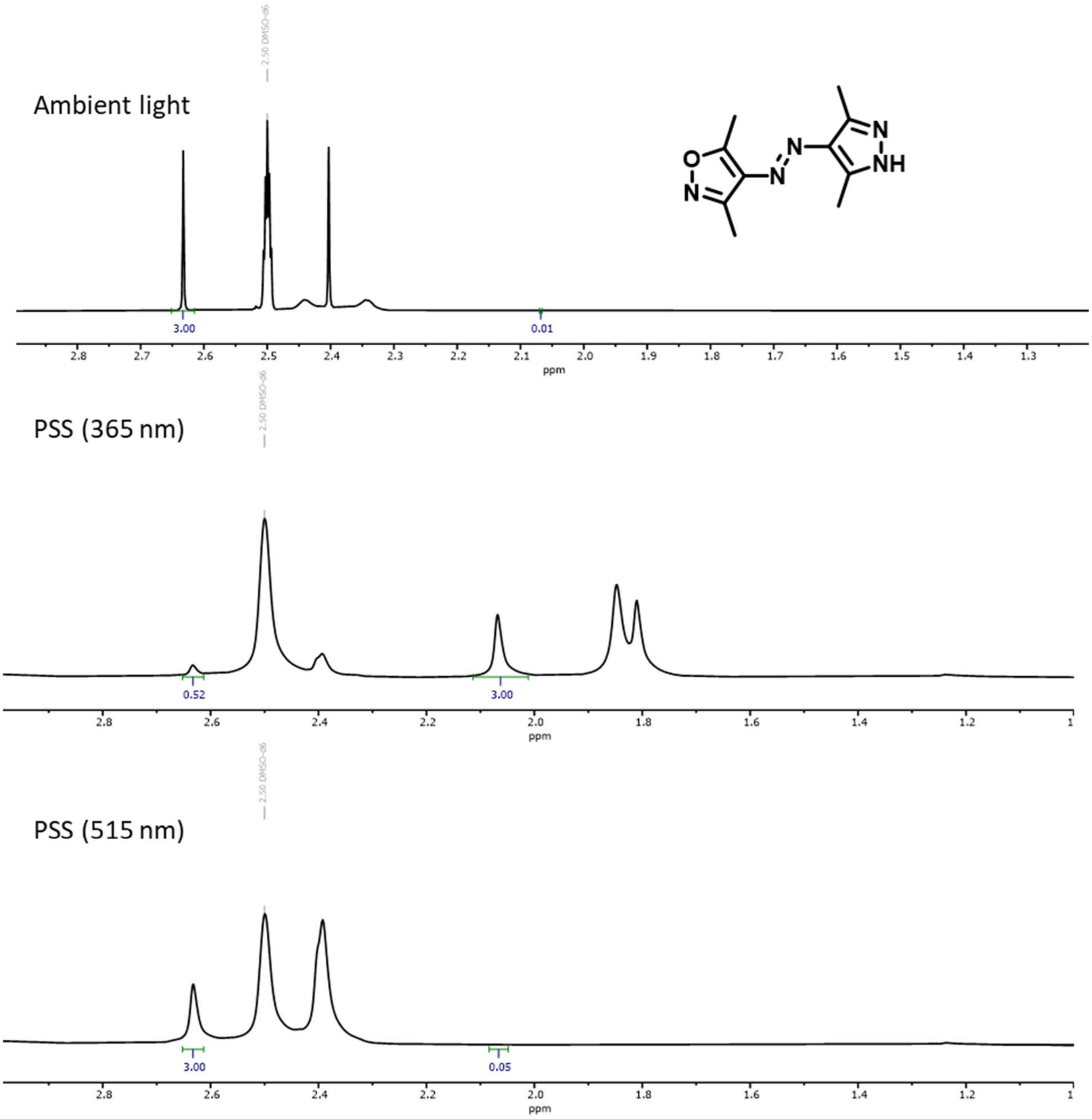

Figure S21: ^{1}H NMR of **4iz-pzH** (5 mg/mL, DMSO-d_6, 400 MHz, 298 K), before irradiation and after irradiation with 365 nm or 515 nm. Irradiation time: 20 min + in situ irradiation.

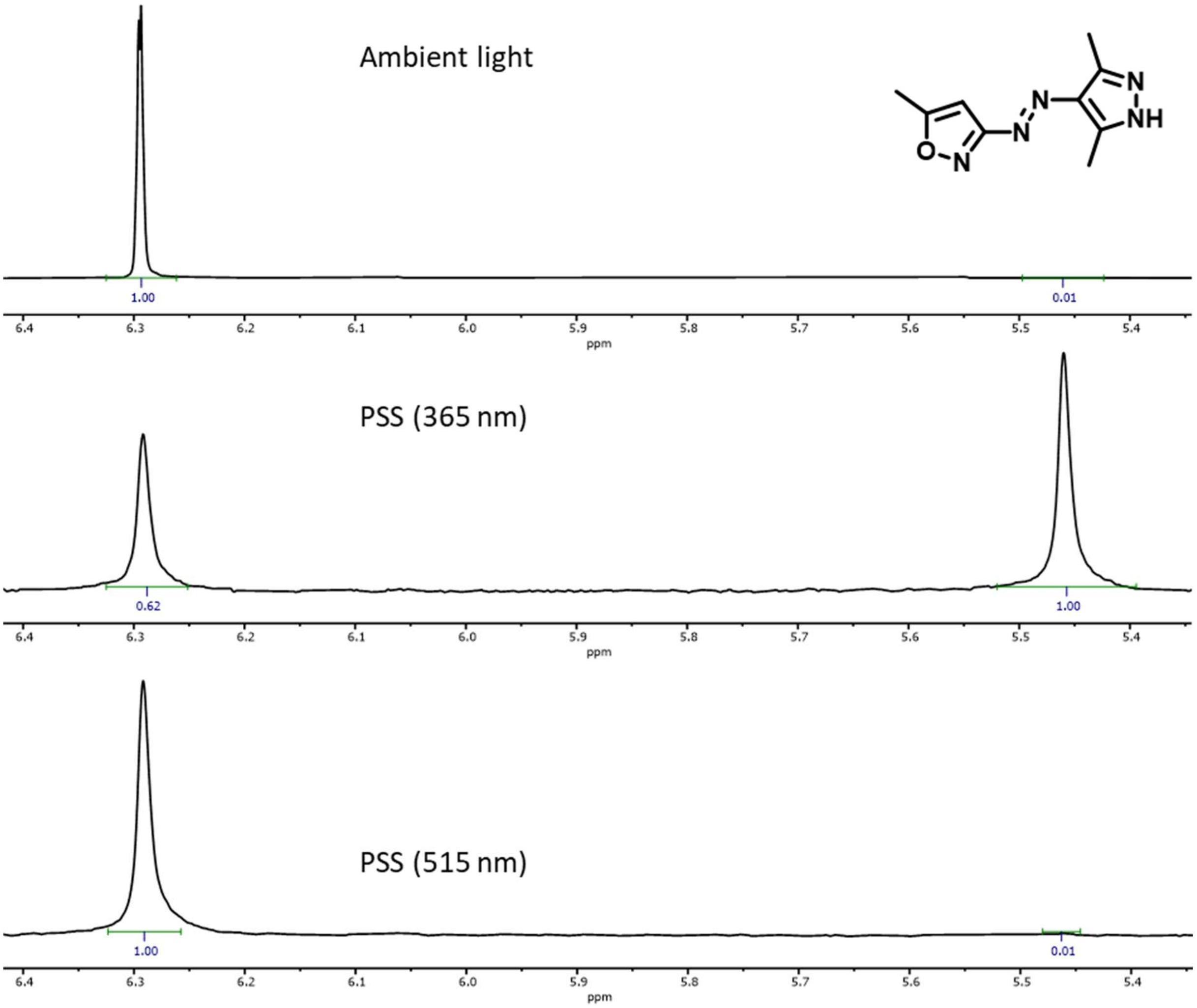

Figure S22: ^{1}H NMR of **3iz-pzH** (5 mg/mL, $CDCl_3$, 400 MHz, 298 K), before irradiation and after irradiation with 365 nm or 515 nm. Irradiation time: 20 min + in situ irradiation.

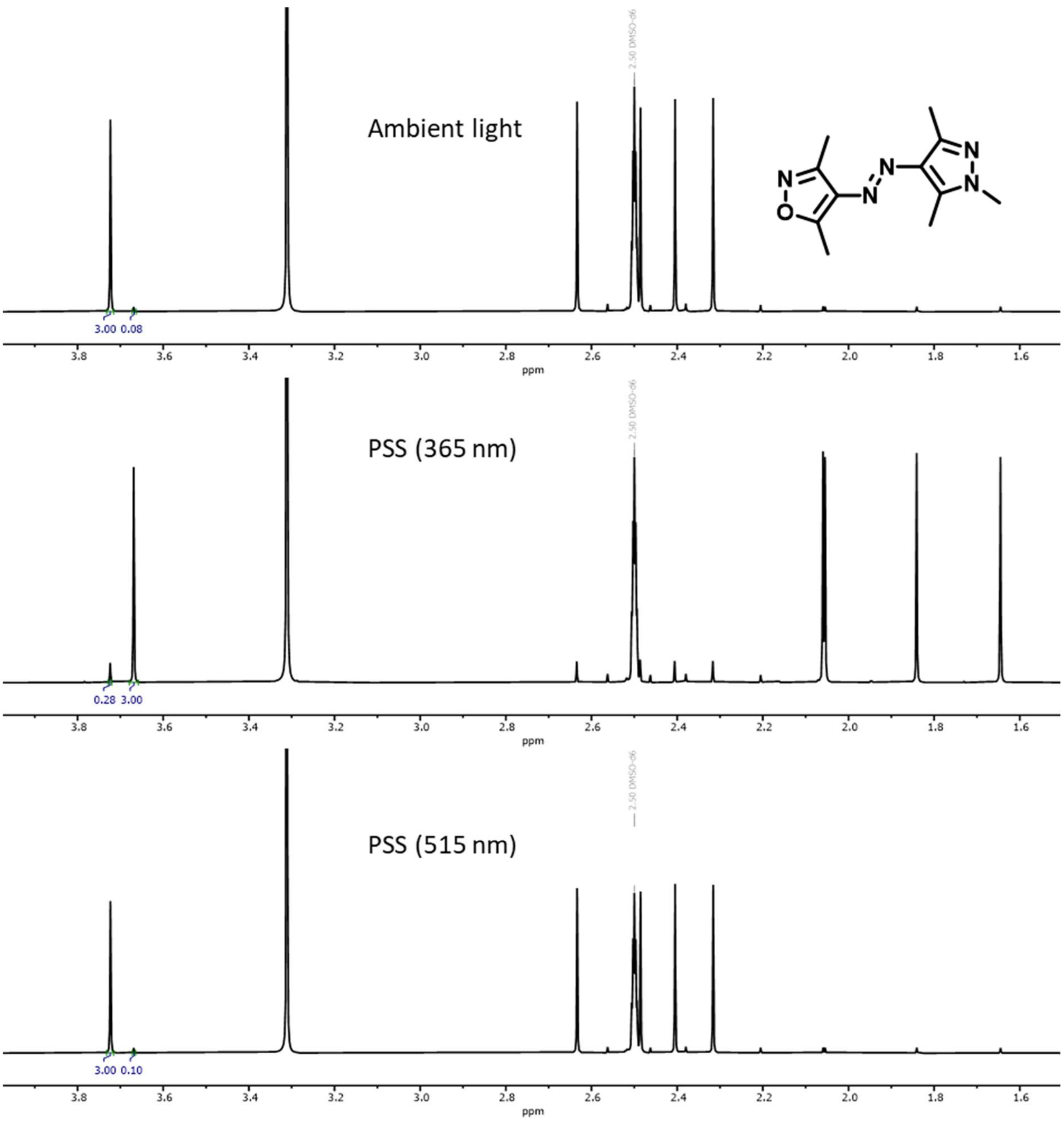

Figure S23: ^{1}H NMR of **4iz-pzMe** (5 mg/mL, DMSO-d_6, 600 MHz, 298 K), before irradiation and after irradiation with 365 nm or 515 nm. Irradiation time: 20 min.

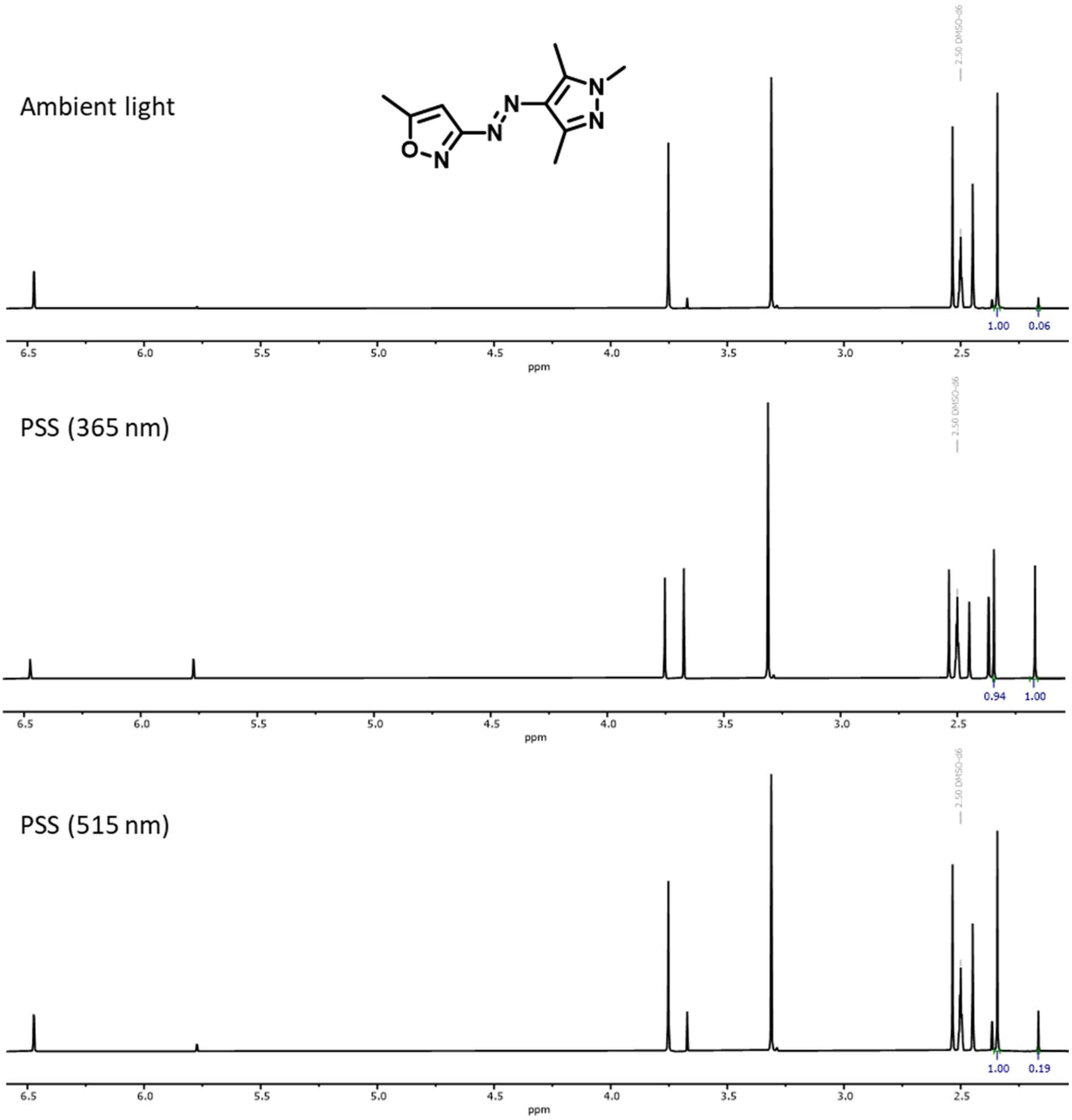

Figure S24: ^{1}H NMR of **3iz-pzMe** (5 mg/mL, DMSO-d_6, 600 MHz, 298 K), before irradiation and after irradiation with 365 nm or 515 nm. Irradiation time: 10 min.

Determination of thermal half-life times

For the determination of thermal half-life times, a solution of the photoswitch in DMSO (50 µM, 25 µM for **4iz-pzH**) was irradiated with UV (365 nm) for 5 min. For the non-methylated pyrazole derivatives **4iz-pzH** and **3iz-pzH**, anhydrous DMSO was used to reduce the influence of residual water. Subsequently, UV/vis spectra were recorded in constant intervals. The absorbance at the maximum of the π–π* transition A_λ was plotted against the time. Assuming first order kinetics, the rate constant *k* was directly calculated by a monoexponential fit function and the final half-life time was obtained via the following equation.

$$t_{1/2} = \ln(2)/k$$

For compounds with high half-life times (> 1 d) this measurement was repeated at elevated temperature (70 °C, 75 °C, 80 °C, 85 °C, 90 °C) the half-life time was extrapolated to room temperature using the linearized Eyring equation as previously described in detail.[4]

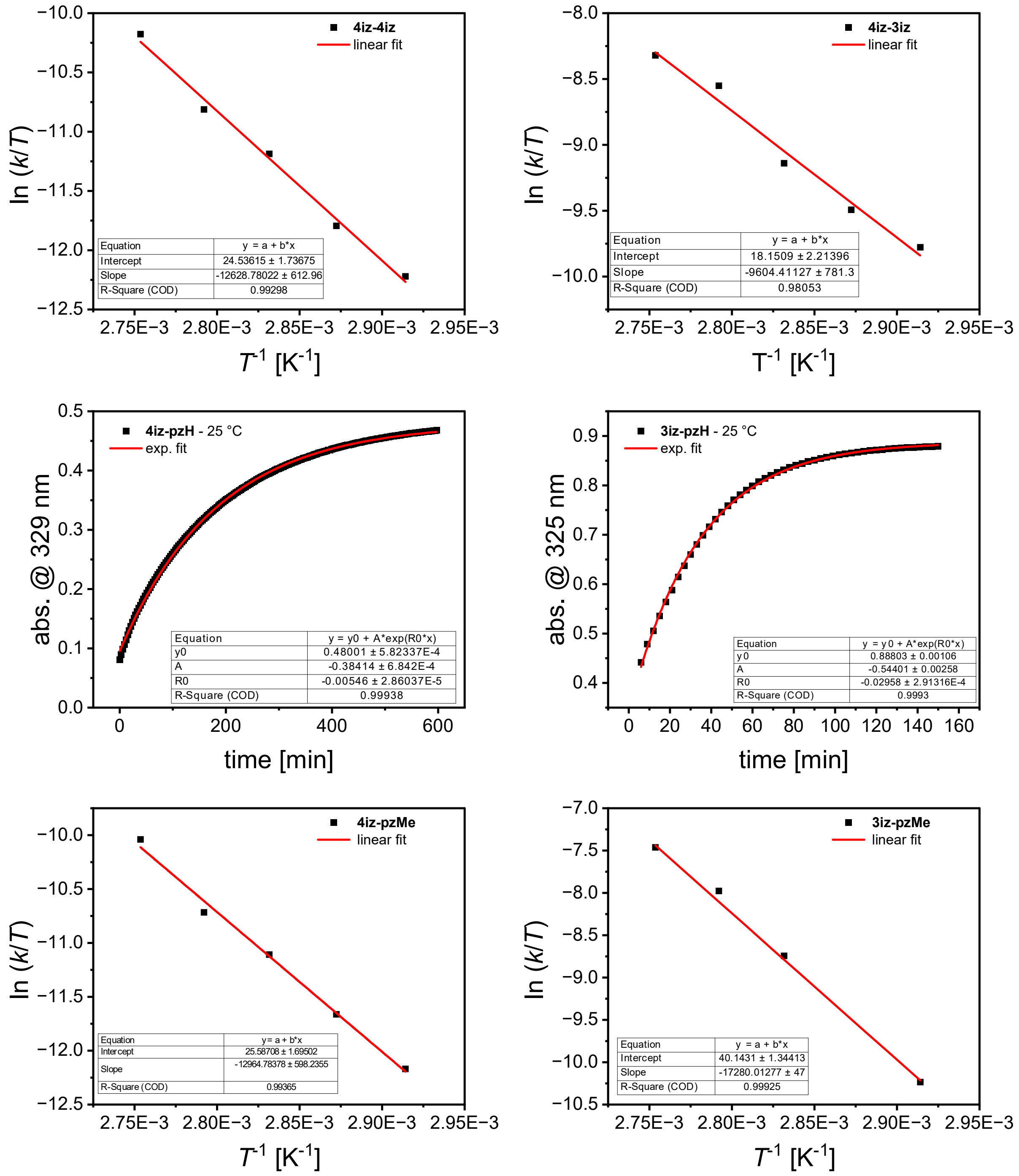

Figure S25: Determination of thermal half-life times via UV/vis spectroscopy.

UV/vis spectroscopy in alternative solvents

Aqueous solution:

For UV/vis spectroscopy in aq. solution, stock solutions in DMSO (1 mM) were prepared for all compounds. For compounds **4iz-pzH**, **3iz-pzH**, **4iz-pzMe** and **3iz-pzMe**, 50 µL of the stock solution was diluted with additional DMSO (50 µL) and ddH_2O (900 µL) and mixed until homogeneous. For compounds **4iz-4iz** and **3iz-4iz**, 25 µL stock solution and 75 µl DMSO were used instead.

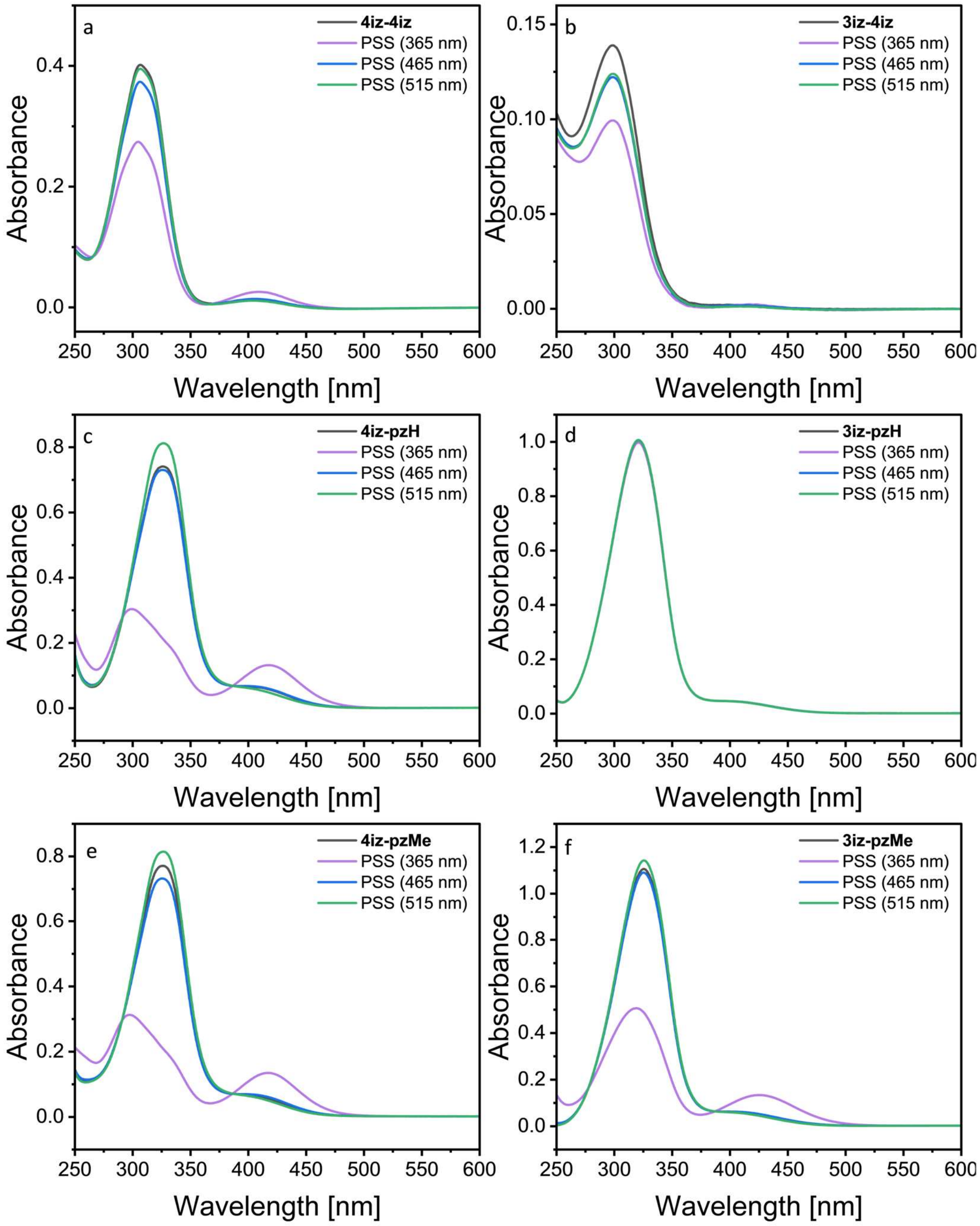

Figure S26: UV/vis spectra before and after irradiation with 365 nm, 465 nm and 515 nm in a mixture of ddH_2O and DMSO (9/1. v/v,50 µM). Irradiation time: 10 min.

Acetonitrile

In acetonitrile, small differences in the position of absorption bands were observed. This led to small differences in PSS for the different wavelengths and resulted in a slightly higher PSS using 310 nm compared to 365 nm in compounds **4iz-pzH** and **3iz-pzH**. However, general photoswitching and degradation behavior showed no difference compared to DMSO.

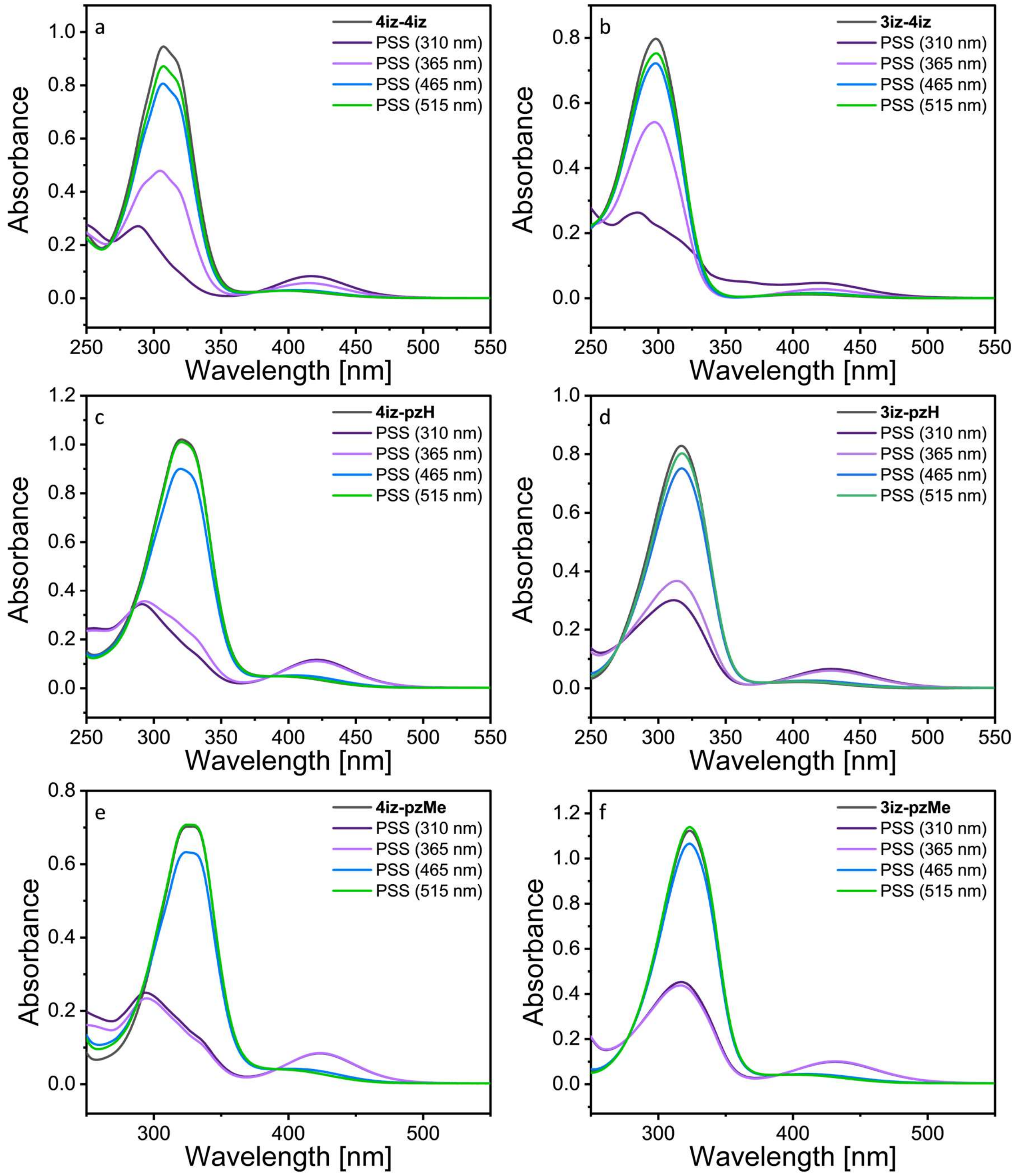

Figure S27: UV/vis spectra before and after irradiation with 365 nm, 465 nm and 515 nm in acetonitrile (50 µM). Irradiation time: 10 min.

Sensitized isomerization of BIZs and IPZs via DESC

Mechanism of disequilibration by sensitization under confinement

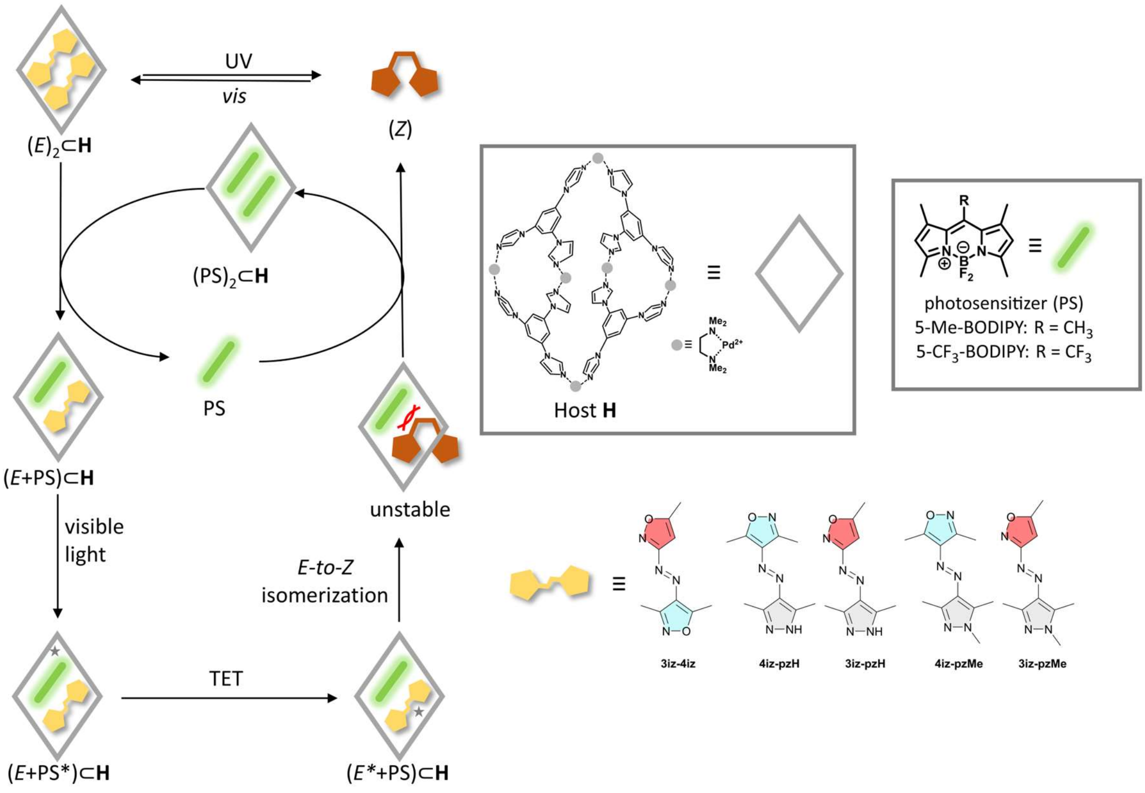

Figure S28: Mechanism of DESC.

1) Water-insoluble photoswitches and sensitizers are encapsulated to form the homodimer complexes $(E)_2$⊂**H** and $(PS)_2$⊂**H** (this step is not shown in Fig. S28).
2) By mixing both homodimer complexes, the ternary complex (*E*+PS)⊂**H** is formed.
3) The PS is excited by irradiation with visible light, followed by intersystem crossing.
4) Facilitated by the close proximity in the complex, triplet photoswitch is formed via triplet-energy transfer.
5) The excited triplet azo relaxes into a) the *E* isomer or b) the *Z* isomer. In case of a) the complex (*E*+PS)⊂**H** is recovered and available for the next cycle. In case of b) the unstable (*Z*+PS)⊂**H** inclusion complex is formed, and the complex disassembles. Because of the increased steric demand of the *Z* isomer, no *Z*-to-*E* isomerization occurs via this mechanism, leading to a selective isomerization and depletion of the *E* isomer.

A detailed investigation of this mechanism can be found in previous studies by Klajn and coworkers[5].

Encapsulation of Photosensitizers **G** and **Y**:

All DESC experiments were performed according to reported literature procedures.[5]

An excess (5-10 eq.) solid sensitizer was added to a solution of **H** (5 mg, 1.6 µmol) in water (0.5 ml) and stirred overnight at r.t. in the dark.

Encapsulation of IPZs and BIZs:

2.0 eq. of photoswitch was added to a solution of **H** (5 mg, 1.6 µmol) in water (0.5 ml) and stirred overnight at r.t. in the dark.

Initial Screening:

For the initial screening, 1 eq. of encapsulated photosensitizer was added to a solution of encapsulated photoswitch. The resulting mixture was analysed via UV/vis spectroscopy for changes in absorbance and response to visible light (515 nm for **G** and 550 nm for **Y**).

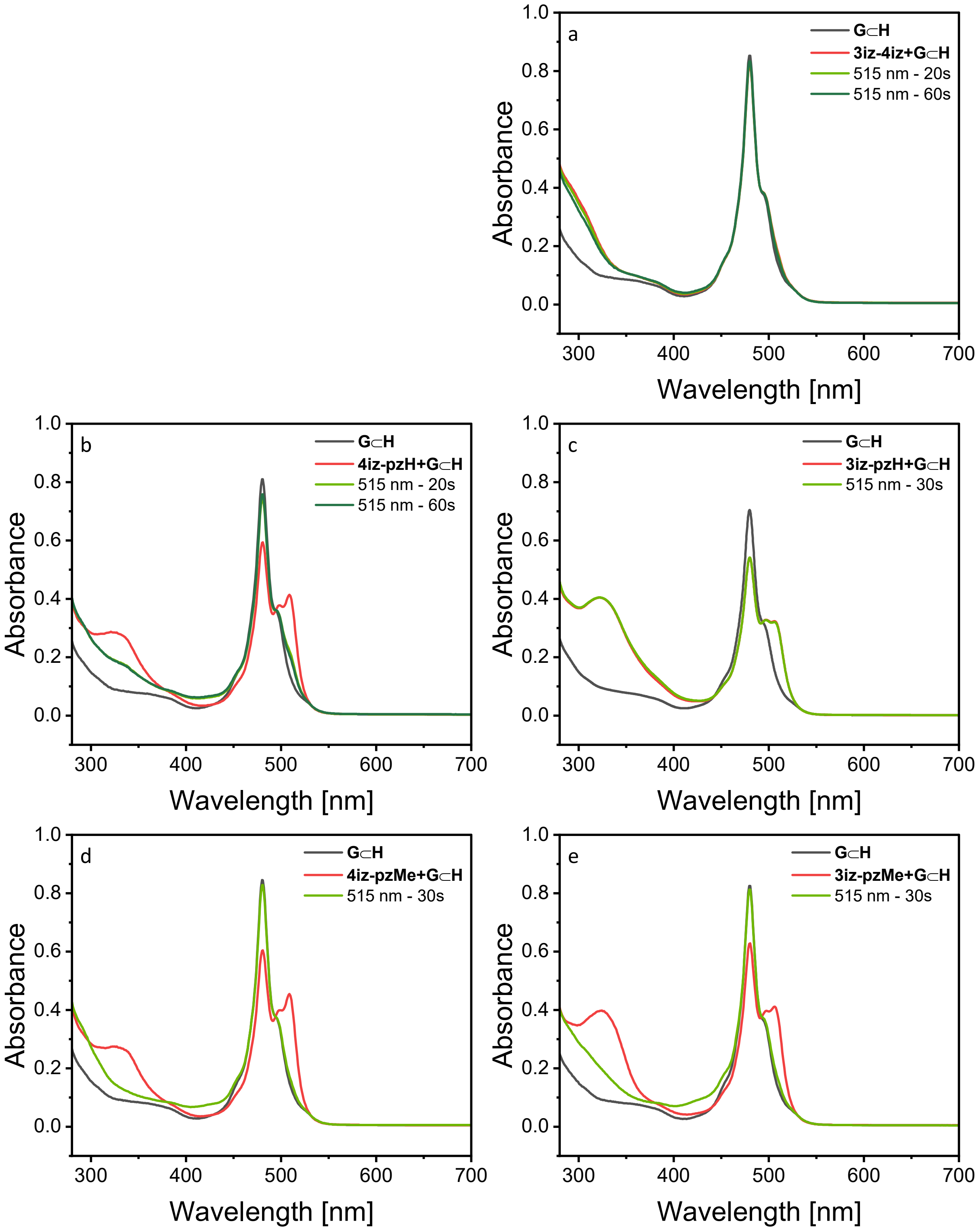

Figure S29: Initial screening of DESC using sensitizer **G**. (H_2O, 20 µM encapsulated photoswitch, 20 µM encapsulated photosensitizer).

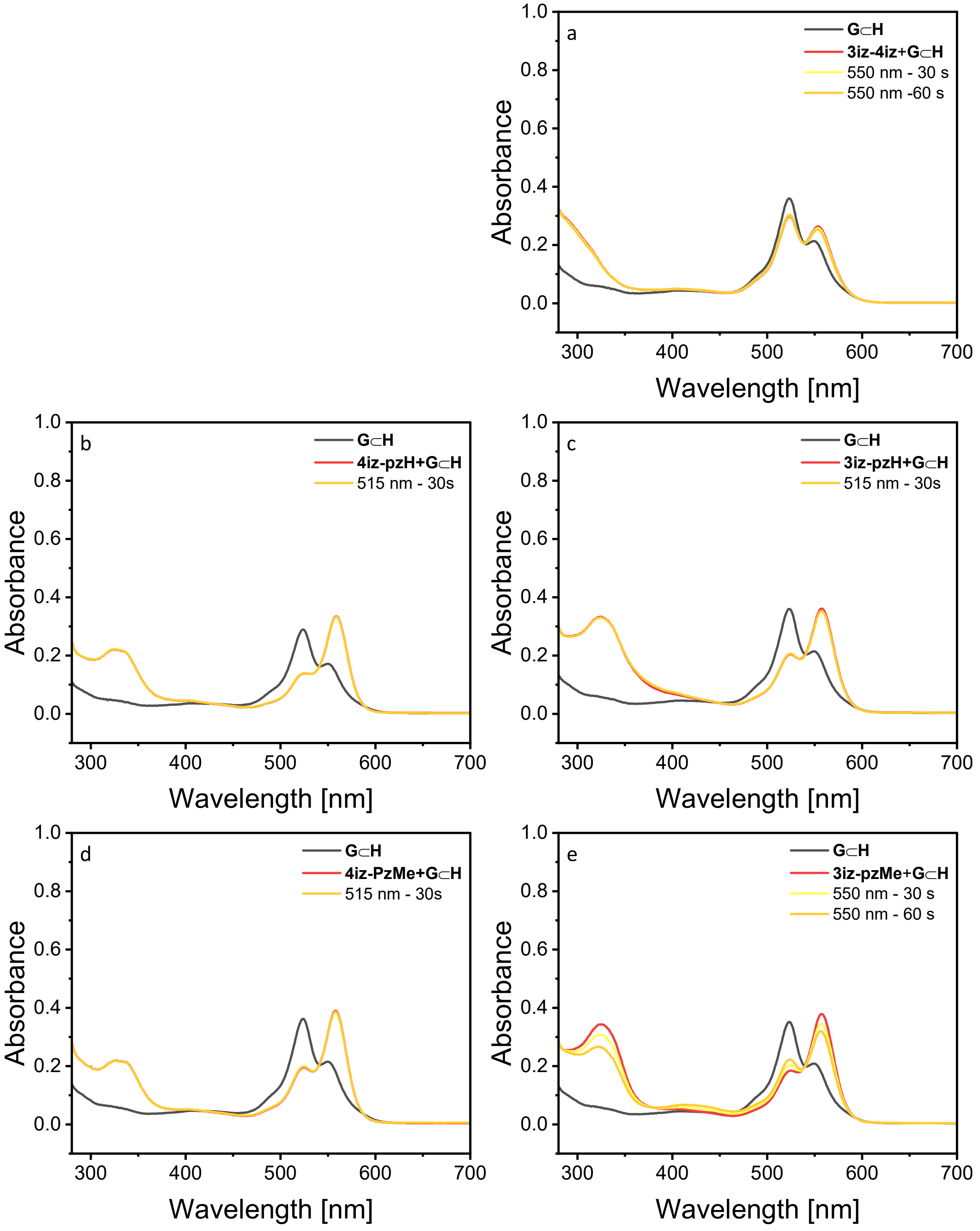

Figure S30: Initial screening of DESC using sensitizer **Y**. (H_2O, 20 µM encapsulated photoswitch, 20 µM encapsulated photosensitizer).

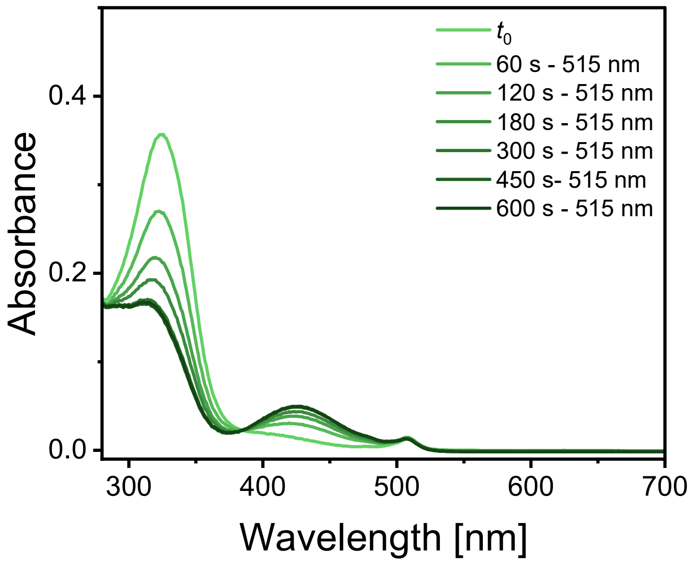

Figure S31: Isomerization via DESC using low loading of **G**. (H_2O, 20 µM encapsulated photoswitch, 0.2 µM Photosensitizer, 0.01 eq.).

<u>NMR Studies of **3iz-4iz** under DESC conditions:</u>

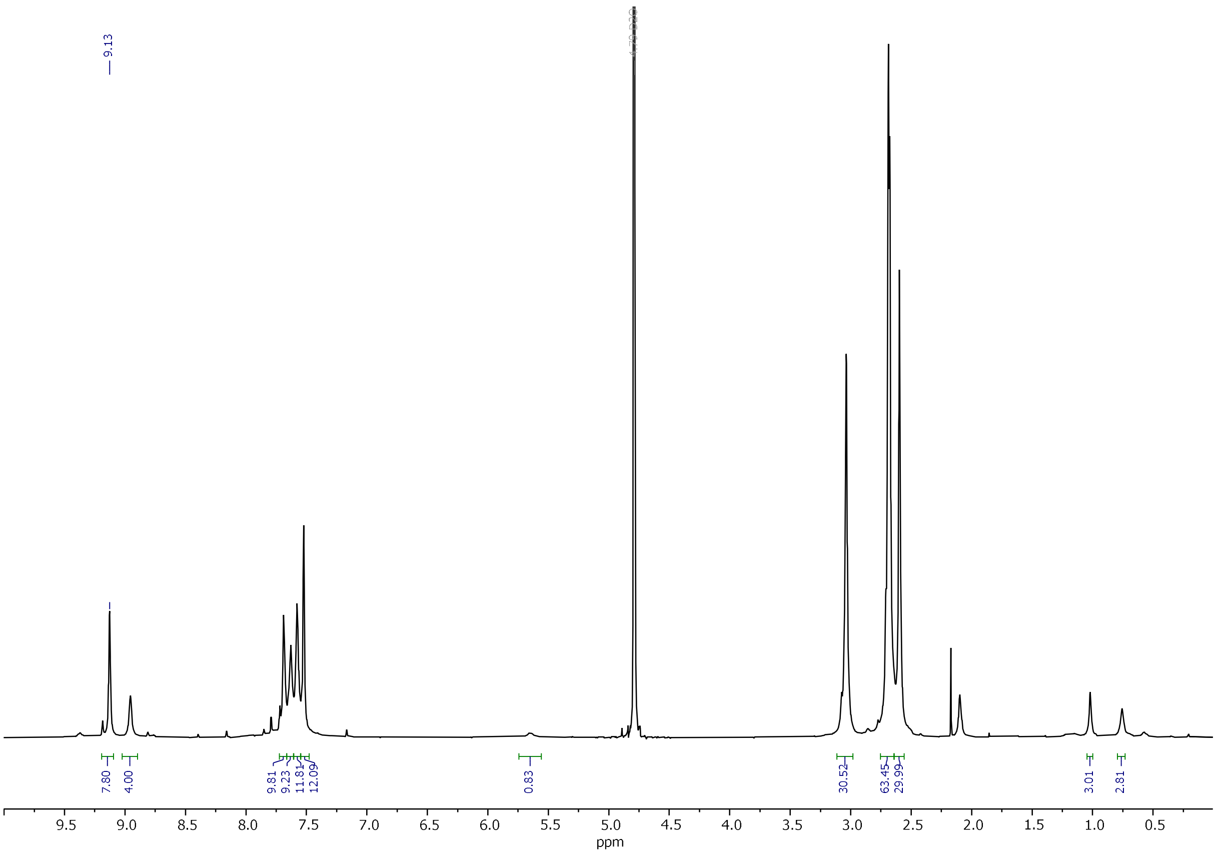

Figure S32: ¹H NMR of **3iz-4iz** encapsulated in **H** (8 mM, 0.5 eq. **H**) and 0.05 eq. of **G** (D_2O, 400 MHz, 298K).

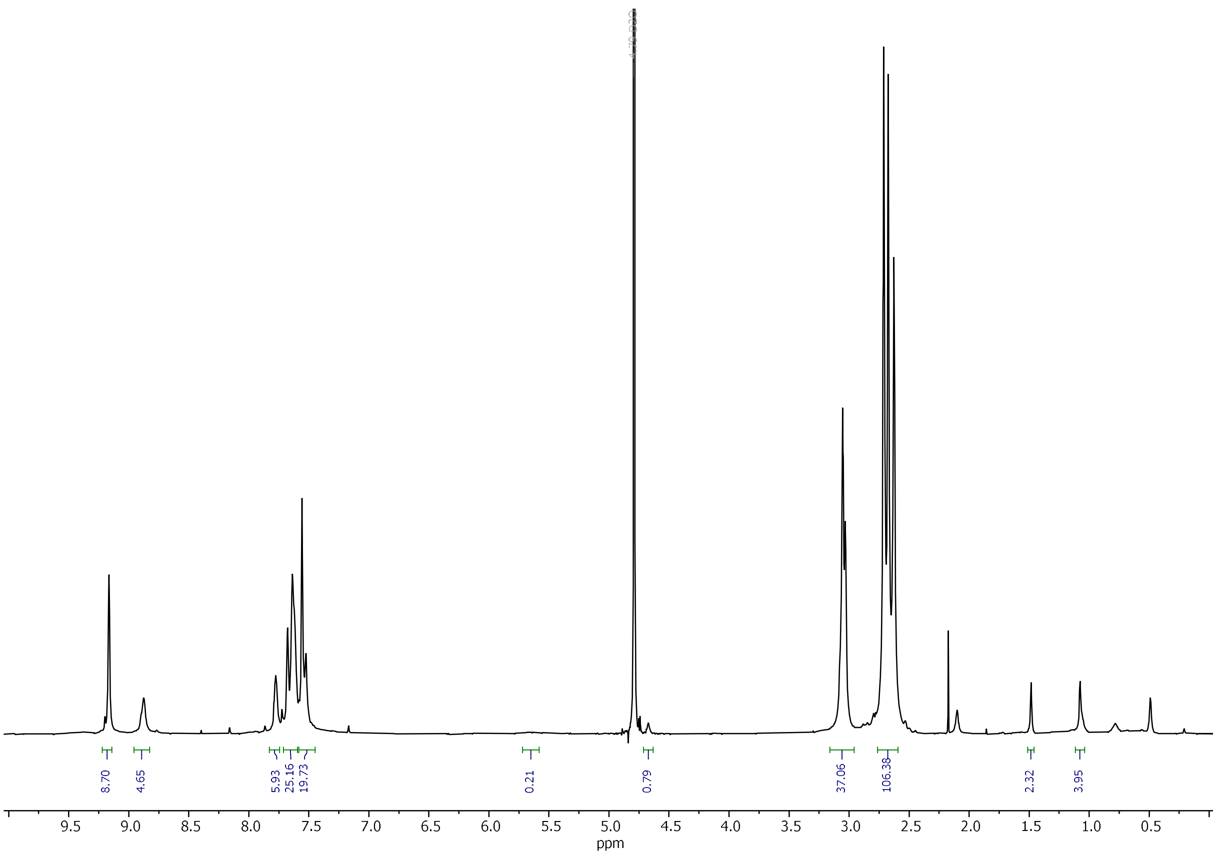

Figure S33: ^{1}H NMR of **3iz-4iz** encapsulated in **H** (8 mM, 0.5 eq. **H**) and 0.05 eq. of **G** after irradiation with green light (5 min) (D_2O, 400 MHz, 298K).

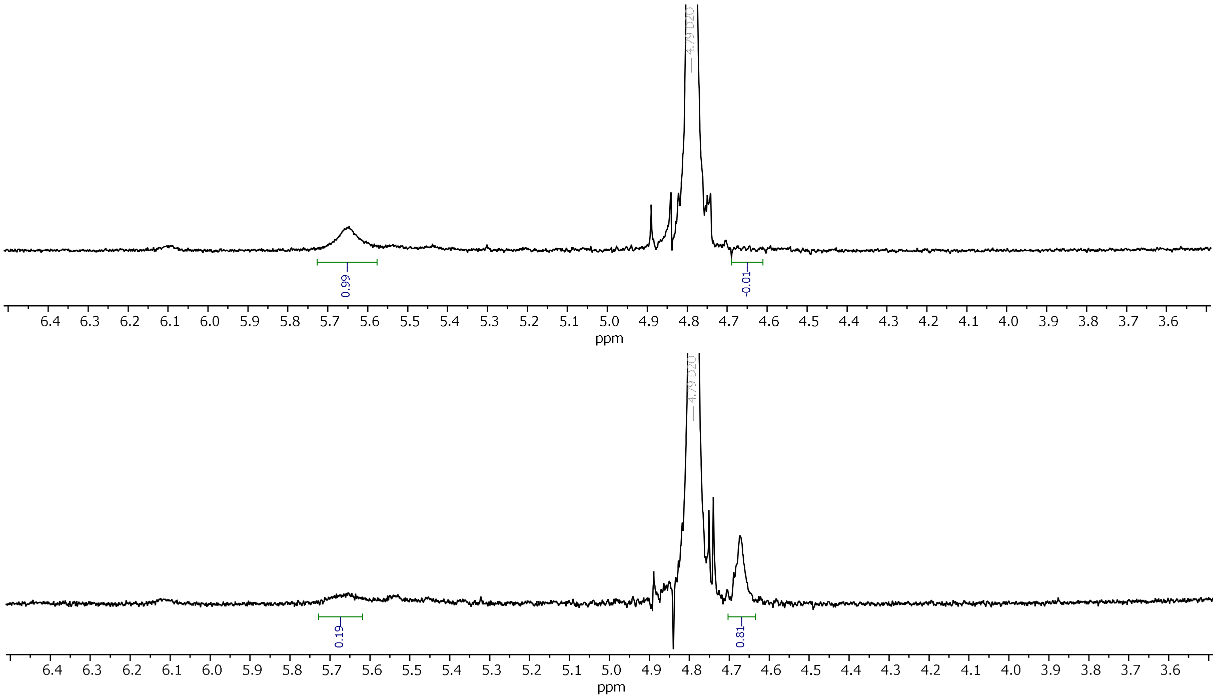

Figure S34: Zoom-in of ^{1}H NMR on the aromatic proton of **3iz-4iz** encapsulated in **H** (8 mM, 0.5 eq. **H**) and 0.05 eq. of **G** before (top) and after (bottom) irradiation with green light (5 min) (D_2O, 400 MHz, 298K). Integration shows a PSS of approximately 81 %.

NMR studies of **3iz-pzMe** under DESC conditions:

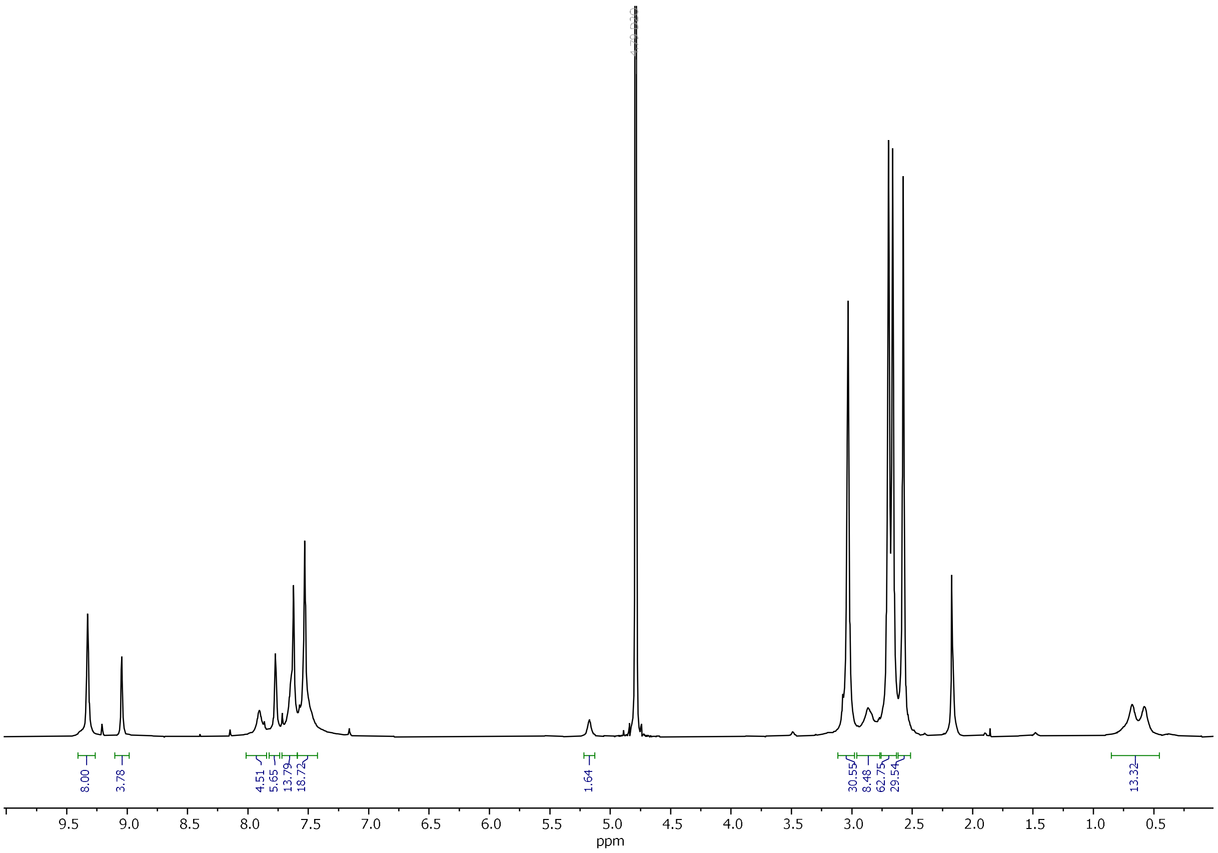

Figure S35: ^{1}H NMR of **3z-pzMe** encapsulated in **H** (8 mM, 0.5 eq. **H**) and 0.05 eq. of **G** (D_2O, 400 MHz, 298K).

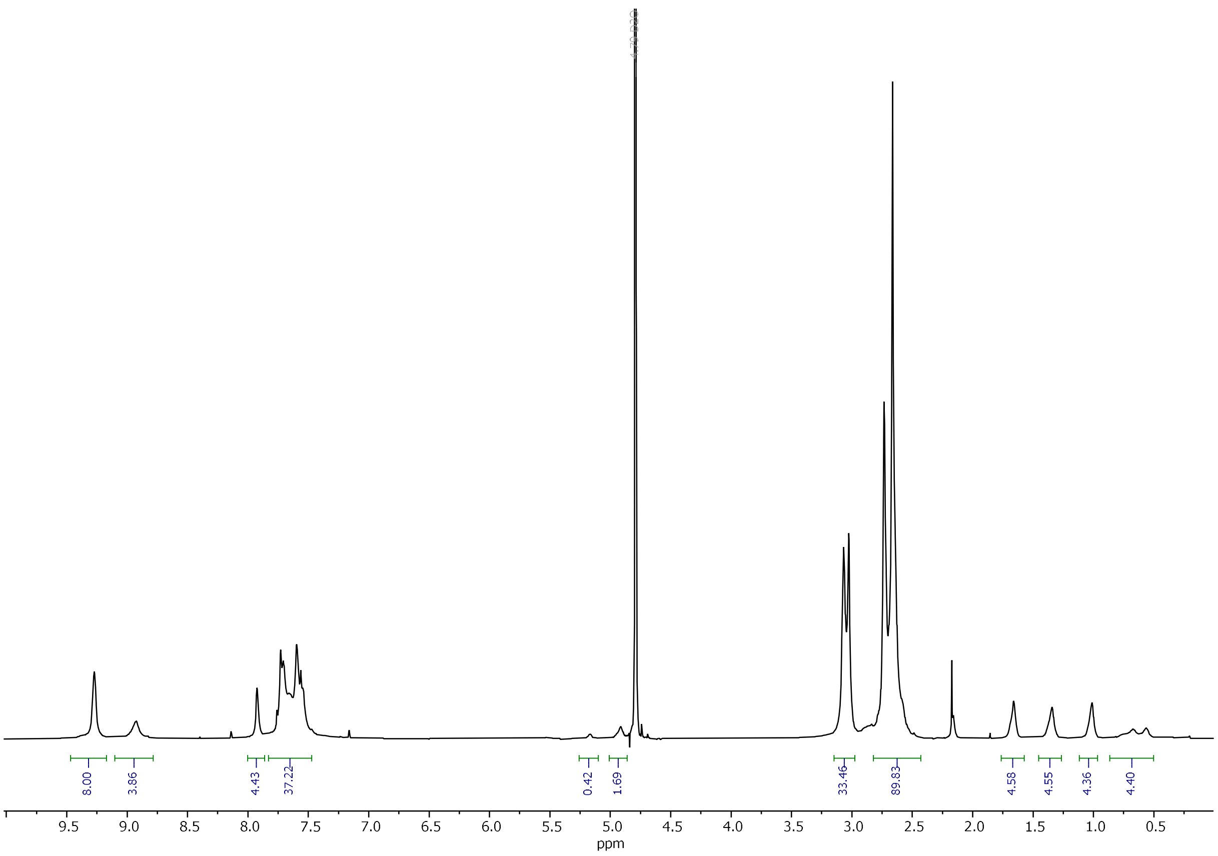

Figure S36: ^{1}H NMR of **3iz-pzMe** encapsulated in **H** (8 mM, 0.5 eq. **H**) and 0.05 eq. of **G** after irradiation with green light (5 min) (D_2O, 400 MHz, 298K).

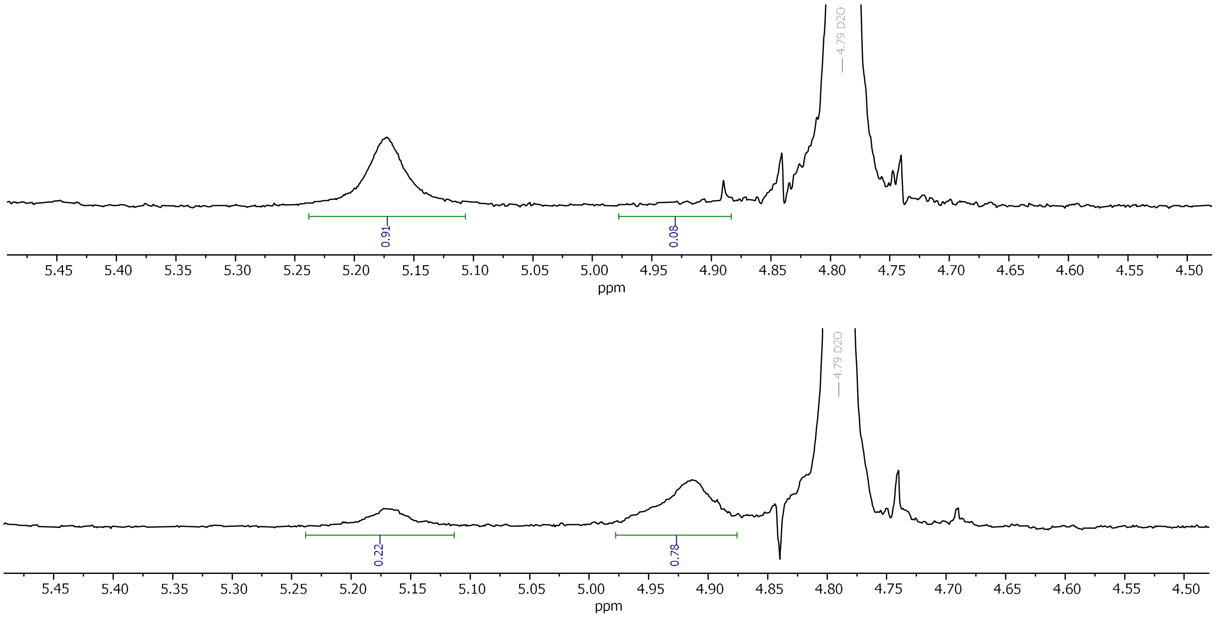

Figure S37: Zoom-in of ^{1}H NMR on the aromatic proton of **3iz-pzMe** encapsulated in **H** (8 mM, 0.5 eq. **H**) and 0.05 eq. of **G** before (top) and after (bottom) irradiation with green light (5 min) (D_2O, 400 MHz, 298K). Integration shows a PSS of approximately 78 %.

Computational details

Theoretical calculations were performed using the Gaussian 16 (Revision B.01)[6] suite of programs. All possible conformers for *E* and *Z* isomers were fully optimized in the gas phase at the B3LYP-D3(BJ)/6-31G** level of theory.[7-15] Subsequently, vibrational frequency analysis was performed to ensure the reliability of the acquired minima on the potential energy surface and check that no imaginary frequences are present. Boltzmann-weighted dipole moments were calculated by weighing the individual dipole moments for each conformer according to the following formula[16] (T = 298 K), where G_i is the Gibbs free energy and μ_i the dipole moment of the *i*-th conformer and G_0 is the Gibbs free energy of the lowest energy conformer. All figures of the optimized geometries were rendered using VMD 1.9.3.[17,18]

$$\langle \mu \rangle = \frac{\sum_{i=1}^{N} \mu_i \cdot \mathrm{e}^{-\frac{G_i - G_0}{k_\mathrm{B} T}}}{\sum_{i=1}^{N} \mathrm{e}^{-\frac{G_i - G_0}{k_\mathrm{B} T}}}$$

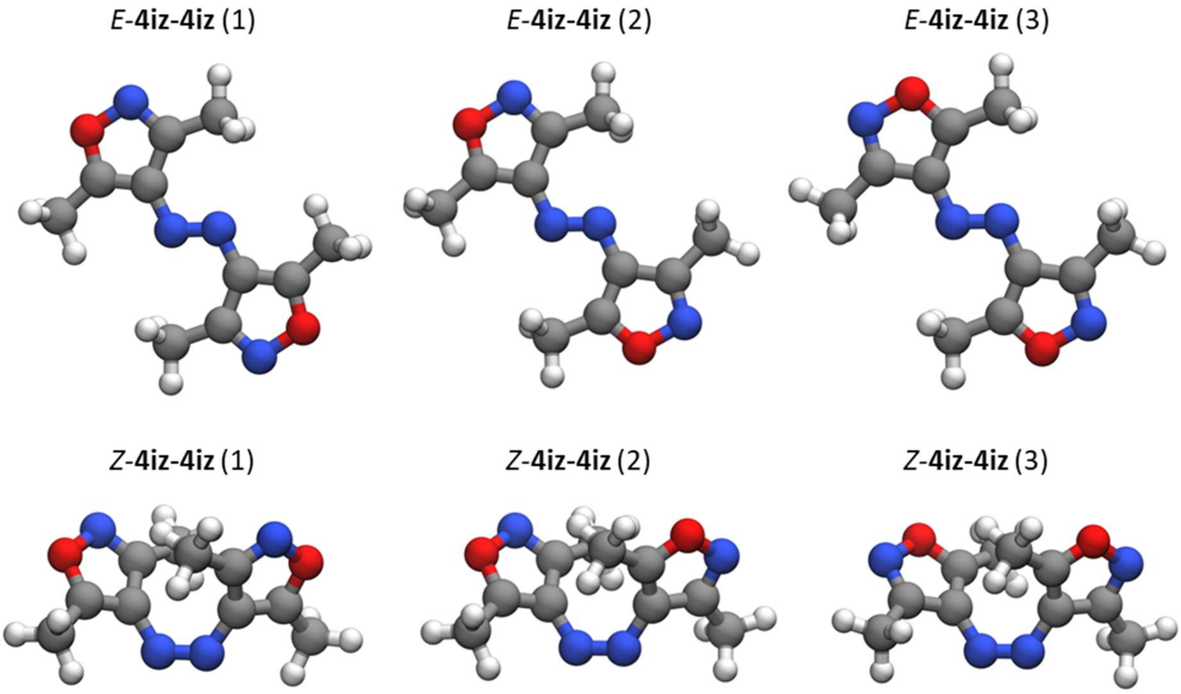

Figure S38: Optimized geometries of the conformers of **4iz-4iz**.

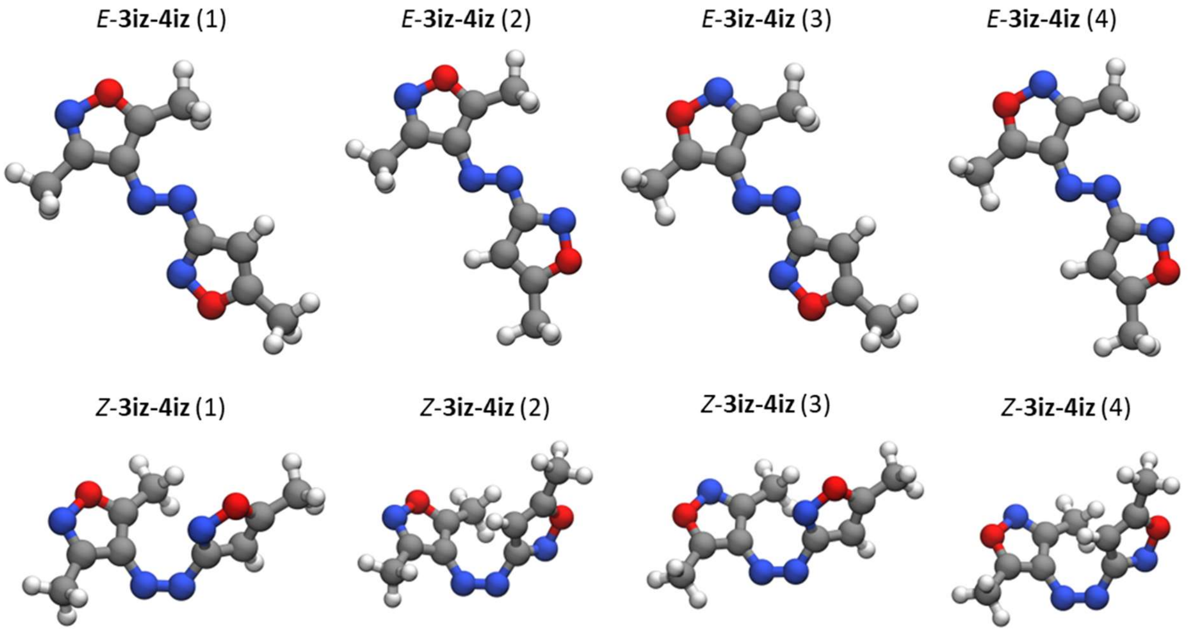

Figure S39: Optimized geometries of the conformers of **3iz-4iz**.

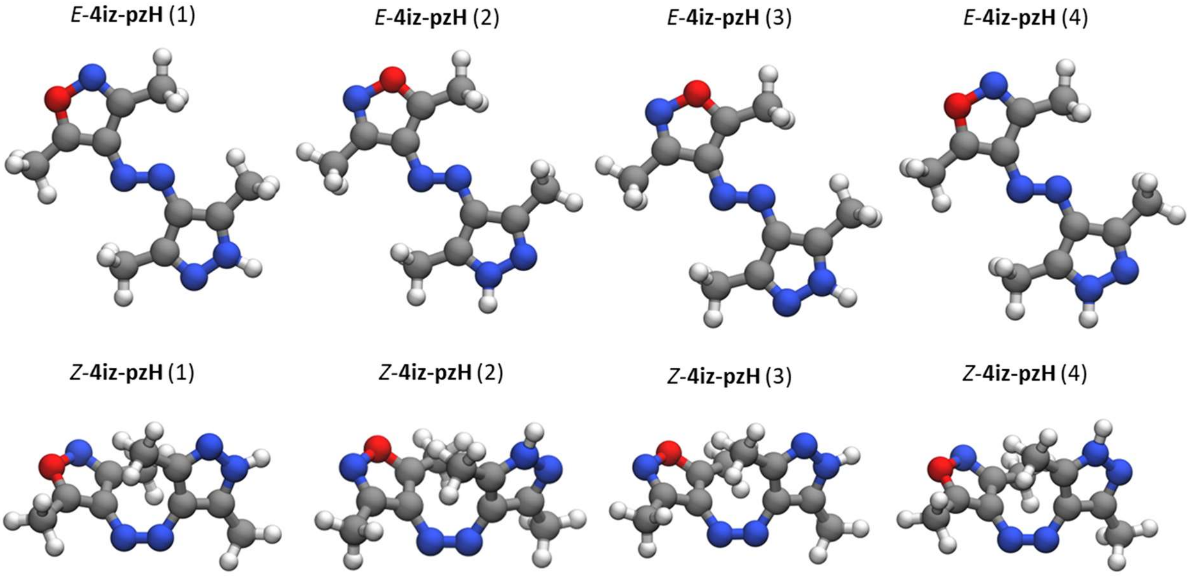

Figure S40: Optimized geometries of the conformers of **4iz-pzH**.

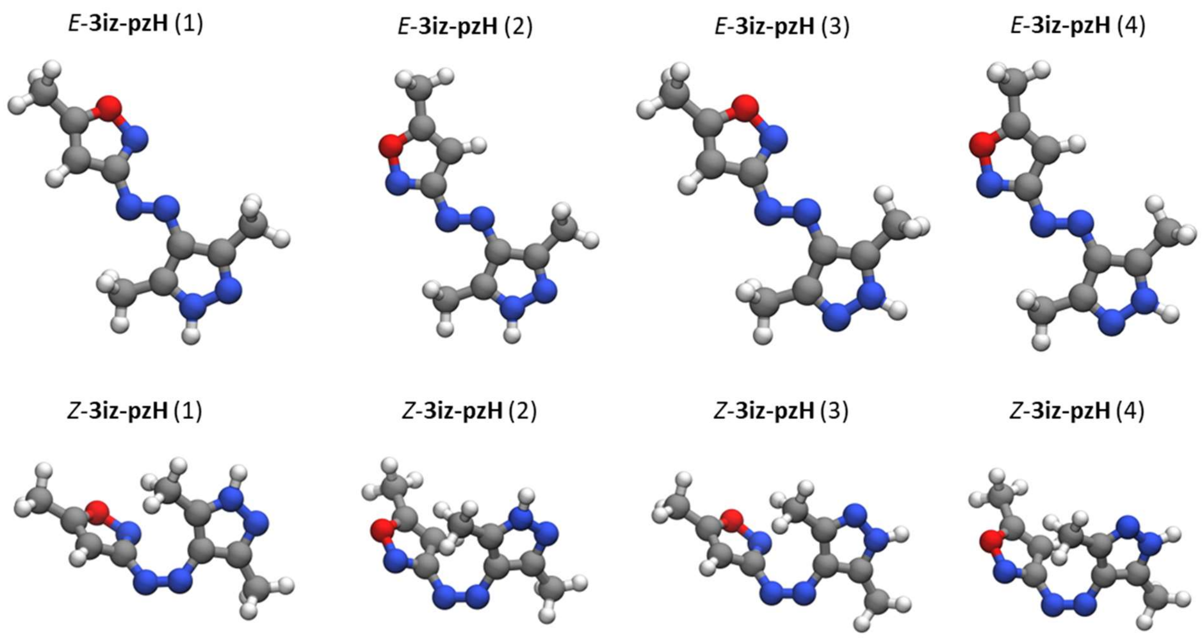

Figure S41: Optimized geometries of the conformers of **3iz-pzH**.

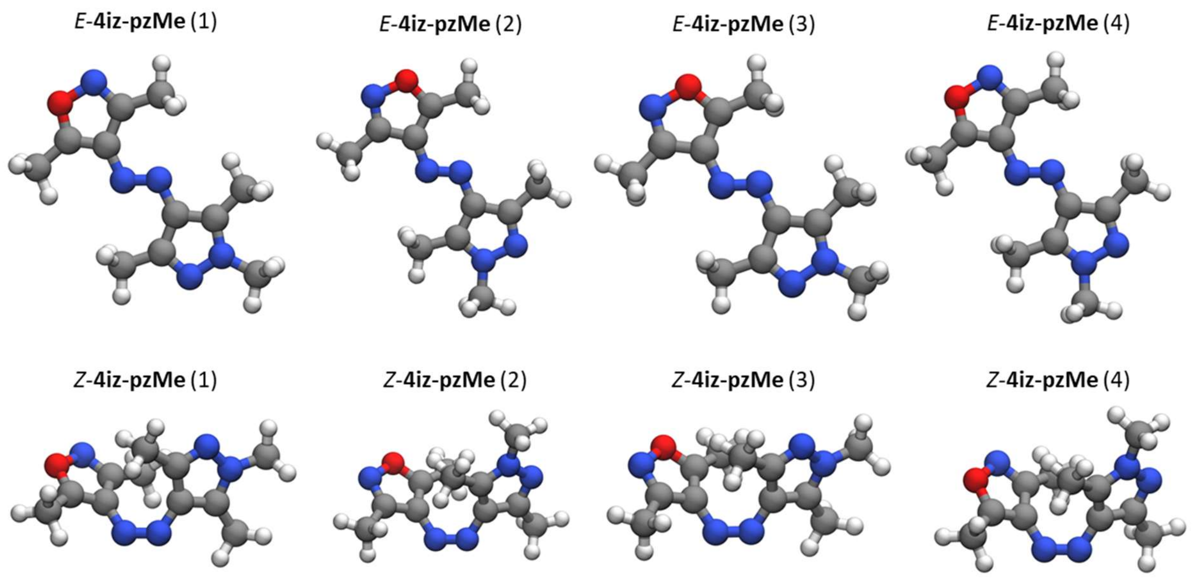

Figure S42: Optimized geometries of the conformers of **4iz-pzMe**.

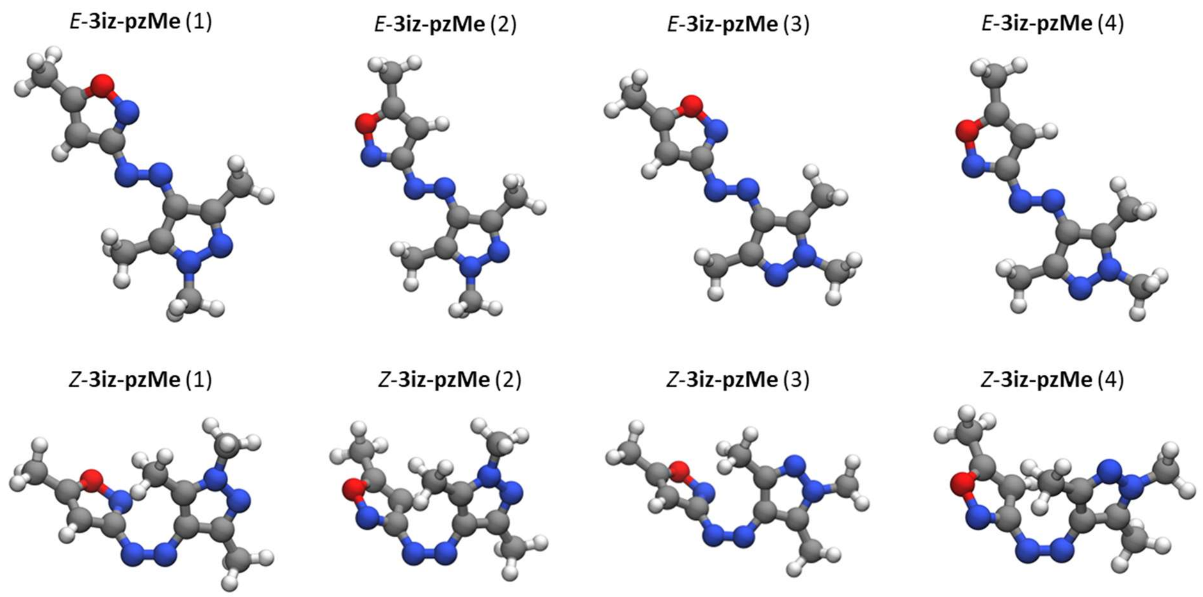

Figure S43: Optimized geometries of the conformers of **3iz-pzMe.**

Table S3: Relative energies of the optimized conformers.

Compound	Conformer	Rel. Energy (kJ/mol)	Compound	Conformer	Rel. Energy (kJ/mol)
**4iz-4iz**	***E* (1)**	**0.00**	**3iz-pzH**	*E* (1)	16.02
	E (2)	5.11		***E* (2)**	**0.00**
	E (3)	9.65		*E* (3)	11.87
	Z (1)	0.39		*E* (4)	0.10
	***Z* (2)**	**0.00**		*Z* (1)	2.89
	Z (3)	0.96		*Z* (2)	0.48
				***Z* (3)**	**0.00**
				Z (4)	0.68
**3iz-4iz**	***E* (1)**	**0.00**	**4iz-pzMe**	***E* (1)**	**0.00**
	E (2)	4.34		*E* (2)	8.78
	E (3)	12.54		*E* (3)	5.11
	***E* (4)**	**0.00**		*E* (4)	4.73
	Z (1)	3.18		***Z* (1)**	**0.00**
	Z (2)	1.64		*Z* (2)	2.70
	***Z* (3)**	**0.00**		*Z* (3)	0.29
	Z (4)	2.03		*Z* (4)	1.25
**4iz-pzH**	***E* (1)**	**0.00**	**3iz-pzMe**	*E* (1)	18.43
	E (2)	6.18		*E* (2)	2.51
	E (3)	5.21		*E* (3)	11.77
	E (4)	2.12		***E* (4)**	**0.00**
	***Z* (1)**	**0.00**		*Z* (1)	4.44
	Z (2)	1.74		*Z* (2)	1.35
	Z (3)	0.29		***Z* (3)**	**0.00**
	Z (4)	0.48		*Z* (4)	0.58

Table S4: Boltzmann-weighted (Gibbs Energy) electric dipole moments.

**Compound (*E*)**	**Dipole moment**	**Compound (*Z*)**	**Dipole moment**
E-**4iz-4iz**	0.45 D	*Z*-**4iz-4iz**	1.37 D
E-**3iz-4iz**	4.38 D	*Z*-**3iz-4iz**	3.69 D
E-**4iz-pzH**	3.93 D	*Z*-**4iz-pzH**	3.27 D
E-**3iz-pzH**	3.17 D	*Z*-**3iz-pzH**	4.48 D
E-**4iz-pzMe**	4.44 D	*Z*-**4iz-pzMe**	4.05 D
E-**3iz-pzMe**	4.99 D	*Z*-**3iz-pzMe**	4.65 D

Cartesian coordinates

***E* 4iz-4iz (1)**				***E* 4iz-4iz (2)**			
O	3.09326	-0.88025	-0.07425	C	1.0202	0.20265	0.08036
C	2.54012	0.33821	-0.04759	C	2.50215	0.26882	0.09122
C	3.53305	1.28967	-0.01769	C	3.41002	1.30908	0.06988
C	4.75922	0.52773	-0.02876	N	3.25724	2.6864	0.03
N	4.50779	-0.75718	-0.0622	N	2.04949	3.08668	0.00843
C	1.06146	0.45064	-0.05422	C	1.9012	4.4629	-0.03139
N	3.26045	2.64756	0.01422	C	0.67013	5.07604	-0.05911
N	4.28803	3.39523	0.03929	C	-0.7084	4.52944	-0.05561
C	4.01543	4.75312	0.07121	O	0.83593	6.40344	-0.09487
C	5.00836	5.70459	0.10111	N	2.22379	6.70649	-0.09147
O	4.45522	6.92304	0.12778	C	2.84597	5.55475	-0.05387
N	3.04068	6.79996	0.11572	C	4.33785	5.51282	-0.03991
C	2.78926	5.51506	0.08228	C	4.69093	0.66567	0.0989
C	6.48702	5.59216	0.10774	C	6.03128	1.32203	0.09202
C	1.37633	5.03401	0.06135	N	4.57264	-0.63865	0.13441
C	6.17215	1.00877	-0.00785	O	3.17631	-0.8885	0.12942
H	0.78512	1.50558	-0.02984	H	0.6107	0.74262	0.9392
H	0.62892	-0.05564	0.81472	H	0.68976	-0.83747	0.10663
H	0.64018	-0.01184	-0.9526	H	0.62575	0.69355	-0.81424
H	6.76336	4.53722	0.08335	H	-0.65862	3.44018	-0.02468
H	6.90829	6.05463	1.00613	H	-1.25261	4.84005	-0.9533
H	6.91955	6.09845	-0.76119	H	-1.26776	4.88955	0.81383
H	0.69565	5.8875	0.07816	H	4.73618	6.52905	-0.06497
H	1.18015	4.38933	0.92209	H	4.7117	4.94763	-0.89754
H	1.19133	4.4328	-0.83268	H	4.69668	4.9967	0.85429
H	6.35716	1.60996	0.8862	H	6.13634	1.9851	0.9558
H	6.36833	1.65347	-0.86858	H	6.15145	1.93578	-0.80558
H	6.85283	0.15528	-0.02467	H	6.82012	0.56872	0.11988

***E* 4iz-4iz (3)**			
O	3.01563	-0.89919	0.06306
C	2.41244	0.29691	0.04893
C	3.38199	1.28079	0.03866
C	4.6216	0.56094	0.04796
N	4.42469	-0.7343	0.06248
C	0.92966	0.32442	0.04726
N	3.30672	2.66429	0.02289
N	2.12004	3.12825	0.01653
C	2.04477	4.51176	7.64E-4
C	3.01432	5.49564	-0.00951
O	2.41113	6.69173	-0.02363
N	1.00207	6.52685	-0.02306
C	0.80516	5.23161	-0.00855
C	4.4971	5.46813	-0.00782
C	-0.57265	4.65807	-0.00331
C	5.99941	1.13447	0.04272
H	0.56049	0.87697	0.91632
H	0.53345	-0.69271	0.05846
H	0.5621	0.85707	-0.8348
H	4.86465	4.93545	0.87423
H	4.89331	6.48525	-0.01899
H	4.86627	4.9156	-0.8769
H	-1.31428	5.45833	-0.01309
H	-0.72497	4.03781	0.88499
H	-0.72334	4.0178	-0.87759
H	6.15011	1.77474	0.917
H	6.15172	1.75475	-0.84559
H	6.74104	0.33422	0.05247

Z 4iz-4iz (1)				*Z* 4iz-4iz (2)			
C	0.26557	4.2075	4.13077	C	1.48064	5.56673	-0.50892
C	1.51106	4.30866	3.3332	C	1.67302	6.87814	0.03928
O	2.34822	3.26136	3.29693	C	2.51986	7.50164	-0.84813
N	3.47587	3.58976	2.51636	O	2.78891	6.64913	-1.85617
C	3.26528	4.80217	2.05781	N	2.11099	5.42918	-1.64981
C	4.32822	5.46315	1.2389	N	1.23595	7.24516	1.32323
C	2.00255	5.31386	2.53692	N	0.80264	8.39864	1.58836
N	1.39413	6.58059	2.48132	C	0.50205	9.35564	0.59916
N	1.36717	7.28618	1.43757	C	0.6761	10.69941	0.82148
C	1.73731	6.79925	0.17119	O	0.19178	11.39149	-0.22119
C	2.3418	7.60186	-0.76502	N	-0.39056	10.49967	-1.14436
O	2.46551	6.92722	-1.91765	C	-0.19659	9.29303	-0.66414
N	1.86206	5.65949	-1.78561	C	1.30518	11.43635	1.94373
C	1.44022	5.58047	-0.54449	C	-0.76884	8.11978	-1.39374
C	0.6879	4.36291	-0.10983	C	3.20001	8.82114	-0.88004
C	2.87287	8.9832	-0.67871	C	0.65021	4.46855	0.06513
H	0.4634	3.7305	5.09423	H	0.71695	12.32141	2.19989
H	-0.4929	3.61203	3.60991	H	2.31697	11.76911	1.68511
H	-0.13312	5.2117	4.29028	H	1.36968	10.77293	2.80885
H	4.08996	5.42664	0.17286	H	-0.00664	7.59665	-1.97669
H	5.27866	4.9516	1.40102	H	-1.55288	8.4621	-2.0715
H	4.43319	6.51337	1.52324	H	-1.19504	7.40145	-0.68812
H	0.31789	3.83428	-0.99014	H	0.79047	3.54997	-0.5065
H	-0.1585	4.64141	0.5233	H	-0.41146	4.73604	0.0481
H	1.32541	3.68404	0.46222	H	0.92569	4.29817	1.10972
H	2.43711	9.47423	0.1941	H	3.41002	9.16208	0.13594
H	3.96329	8.98384	-0.5685	H	4.13747	8.74284	-1.43483
H	2.62652	9.5472	-1.58211	H	2.56964	9.57687	-1.36232

Z 4iz-4iz (3)			
C	1.64679	5.46821	-0.38099
C	1.79642	6.83763	0.02096
C	2.58675	7.39709	-0.95721
O	2.86704	6.45256	-1.87644
N	2.25142	5.23498	-1.52018
N	1.387	7.30373	1.28396
N	0.93628	8.46444	1.47762
C	0.58498	9.33827	0.43215
C	-0.15183	9.15434	-0.71576
O	-0.39506	10.35888	-1.26859
N	0.19193	11.37555	-0.48743
C	0.74224	10.7612	0.53113
C	-0.74205	7.97048	-1.3887
C	1.45741	11.51044	1.60477
C	3.19887	8.73466	-1.15453
C	0.88659	4.40287	0.33501
H	-0.0229	7.50384	-2.07162
H	-1.6252	8.26665	-1.95866
H	-1.02607	7.21744	-0.64993
H	1.09014	11.19795	2.58634
H	1.30783	12.58394	1.48062
H	2.5315	11.29873	1.57776
H	1.0511	3.43554	-0.14186
H	-0.18667	4.6204	0.32998
H	1.20302	4.35707	1.38086
H	3.44002	9.18679	-0.18968
H	4.11128	8.64393	-1.74766
H	2.50988	9.41308	-1.67062

E 3iz-4iz (1)				*E* 3iz-4iz (2)			
O	-2.82892	1.80482	-4.71359	C	-0.76178	1.10957	-3.963
C	-2.13136	0.71971	-4.29402	C	-2.13862	0.7098	-4.35623
C	-2.94985	-0.36713	-4.30541	C	-2.95142	-0.32875	-4.03044
C	-4.19151	0.14354	-4.78637	C	-4.13884	-0.07566	-4.78523
N	-4.13856	1.44291	-5.02585	N	-5.33795	-0.79143	-4.87215
C	-0.70702	0.94	-3.92929	N	-5.33228	-1.83288	-4.14536
N	-5.34681	-0.64562	-4.91946	C	-6.49747	-2.57543	-4.19712
N	-6.21287	-0.16075	-5.71024	C	-7.67734	-2.39484	-4.89776
C	-7.38076	-0.89336	-5.804	C	-8.14746	-1.35868	-5.84721
C	-7.81956	-2.03238	-5.15219	O	-8.5055	-3.40009	-4.6026
O	-9.04054	-2.33604	-5.59779	N	-7.88675	-4.29156	-3.68129
N	-9.45475	-1.38729	-6.57786	C	-6.70078	-3.78554	-3.45499
C	-8.45988	-0.54334	-6.68283	C	-5.74147	-4.45053	-2.52512
C	-7.21876	-2.90697	-4.11729	N	-4.04723	1.02737	-5.50741
C	-8.51798	0.61392	-7.62274	O	-2.77975	1.52631	-5.23641
H	-0.25862	0.00495	-3.58925	H	-0.34903	0.38564	-3.25844
H	-0.62052	1.68205	-3.12933	H	-0.76314	2.09728	-3.49113
H	-0.13732	1.30948	-4.78793	H	-0.10642	1.1606	-4.83828
H	-2.72227	-1.38467	-4.03159	H	-2.7645	-1.15452	-3.36524
H	-6.89214	-2.30558	-3.26439	H	-7.4709	-1.29965	-6.70476
H	-6.32161	-3.39548	-4.50992	H	-8.12005	-0.3735	-5.37259
H	-7.93863	-3.66133	-3.79462	H	-9.16076	-1.5853	-6.18344
H	-9.48046	0.6352	-8.13608	H	-6.18559	-5.35713	-2.11134
H	-7.71525	0.54372	-8.36237	H	-5.47301	-3.77525	-1.70752
H	-8.37393	1.55124	-7.07828	H	-4.81804	-4.70937	-3.05125

E **3iz-4iz (3)**				*E* **3iz-4iz (4)**			
O	-2.87212	1.87273	-4.89099	O	-2.62057	1.45777	-5.18658
C	-2.14777	0.85681	-4.35845	C	-2.04827	0.60119	-4.29756
C	-2.93382	-0.25008	-4.2681	C	-2.92374	-0.39548	-4.00466
C	-4.18501	0.17249	-4.8058	C	-4.07474	-0.0734	-4.78915
N	-4.16715	1.44213	-5.1753	N	-3.90452	1.02923	-5.49725
C	-0.73503	1.15597	-4.00496	C	-0.66374	0.92396	-3.86273
N	-5.31138	-0.66707	-4.87011	N	-5.30834	-0.72254	-4.91754
N	-6.21021	-0.25996	-5.66592	N	-5.38046	-1.76815	-4.2031
C	-7.34404	-1.04453	-5.71162	C	-6.57993	-2.44757	-4.29337
C	-8.4056	-0.7438	-6.53729	C	-6.83037	-3.60251	-3.58638
O	-9.36073	-1.66154	-6.37927	O	-8.06084	-4.03471	-3.8687
N	-8.94115	-2.62409	-5.41334	N	-8.67472	-3.14643	-4.80044
C	-7.74932	-2.24914	-5.02535	C	-7.79155	-2.21243	-5.04474
C	-8.62031	0.36642	-7.49534	C	-6.00048	-4.37674	-2.63244
C	-7.00826	-3.04333	-4.0026	C	-8.11051	-1.10466	-5.99186
H	-0.26248	0.27268	-3.57199	H	-0.30941	0.17323	-3.1544
H	-0.6801	1.97419	-3.27998	H	-0.62616	1.90629	-3.38124
H	-0.16699	1.45828	-4.89056	H	0.01752	0.9477	-4.7192
H	-2.68077	-1.22777	-3.89098	H	-2.7978	-1.23508	-3.34254
H	-8.76652	-0.02027	-8.50897	H	-6.49041	-4.43937	-1.65558
H	-7.74739	1.02047	-7.47846	H	-5.034	-3.8836	-2.52079
H	-9.51179	0.94149	-7.22589	H	-5.84648	-5.39835	-2.99408
H	-7.61139	-3.89679	-3.68698	H	-9.12052	-1.23342	-6.38551
H	-6.76808	-2.4188	-3.13826	H	-7.39019	-1.08722	-6.81359
H	-6.05587	-3.39245	-4.40971	H	-8.02756	-0.13801	-5.48875

Z 3iz-4iz (1)				*Z* 3iz-4iz (2)			
C	-3.62776	5.07747	-3.56365	O	-4.83026	4.23274	-5.31333
C	-4.33168	3.93495	-4.20357	C	-4.14451	3.99301	-4.16941
C	-4.61416	2.66196	-3.8141	C	-4.43202	2.74033	-3.72204
C	-5.31554	2.11038	-4.92698	C	-5.34402	2.23345	-4.70089
N	-5.95968	0.84875	-4.88843	N	-5.57724	3.10201	-5.6629
N	-6.03925	0.06901	-5.86946	C	-3.2658	5.08459	-3.67193
C	-5.36444	0.19736	-7.08713	N	-5.85573	0.91357	-4.83485
C	-4.11219	0.63775	-7.47029	N	-6.37732	0.31897	-3.85854
C	-2.98167	1.31305	-6.78827	C	-6.7011	0.95638	-2.65035
O	-3.91569	0.30504	-8.75337	C	-7.32802	2.15222	-2.36978
N	-5.05498	-0.3593	-9.26898	O	-7.59036	2.19657	-1.05308
C	-5.88856	-0.4413	-8.2648	N	-7.13009	1.01717	-0.42421
C	-7.21758	-1.10382	-8.40407	C	-6.63867	0.2777	-1.38652
N	-5.46433	2.98215	-5.90635	C	-7.81831	3.28735	-3.19132
O	-4.8376	4.14823	-5.44313	C	-6.07364	-1.07779	-1.12256
H	-3.2781	4.793	-2.56995	H	-2.79568	4.78898	-2.73264
H	-4.29609	5.93883	-3.46802	H	-3.84049	6.00076	-3.50441
H	-2.76672	5.39034	-4.16278	H	-2.48168	5.31297	-4.4007
H	-4.37893	2.17388	-2.88212	H	-4.05697	2.2445	-2.84206
H	-2.91142	0.99688	-5.74571	H	-8.04735	2.95762	-4.20552
H	-3.13739	2.39725	-6.79576	H	-7.05603	4.07036	-3.26694
H	-2.04683	1.0815	-7.30238	H	-8.71284	3.71524	-2.73369
H	-7.28553	-1.61426	-9.36591	H	-6.27251	-1.37477	-0.09183
H	-8.02338	-0.36662	-8.33501	H	-4.99236	-1.08889	-1.29223
H	-7.36289	-1.82139	-7.592	H	-6.51735	-1.80582	-1.80755

Z 3iz-4iz (3)				*Z* 3iz-4iz (4)			
O	-4.78958	4.31596	-5.38826	O	-4.52995	4.01908	-5.56059
C	-4.47479	4.04047	-4.09807	C	-3.9772	4.01153	-4.32304
C	-4.92736	2.79751	-3.7781	C	-4.25754	2.82949	-3.70985
C	-5.52136	2.32909	-4.98666	C	-5.02105	2.11514	-4.68661
N	-5.46018	3.22302	-5.95521	N	-5.17315	2.80076	-5.80112
C	-3.7517	5.09999	-3.34661	C	-3.22416	5.22743	-3.91668
N	-6.2799	1.14141	-5.09288	N	-5.44827	0.7634	-4.65045
N	-6.24535	0.38931	-6.0981	N	-6.02749	0.29507	-3.63861
C	-5.33616	0.45917	-7.15842	C	-6.50878	1.08063	-2.57757
C	-5.68899	-0.11618	-8.36113	C	-6.5507	0.58049	-1.29638
O	-4.6611	-0.05321	-9.20922	O	-7.15254	1.46458	-0.4933
N	-3.54457	0.51509	-8.54929	N	-7.60905	2.56162	-1.26039
C	-3.94912	0.8353	-7.34537	C	-7.21652	2.33924	-2.49176
C	-6.96186	-0.72065	-8.81963	C	-6.04129	-0.68714	-0.72053
C	-2.94876	1.41312	-6.39724	C	-7.62952	3.29241	-3.56765
H	-3.56978	4.7742	-2.32122	H	-2.84819	5.11263	-2.89879
H	-4.33587	6.02518	-3.32343	H	-3.86632	6.11279	-3.95584
H	-2.79042	5.32589	-3.819	H	-2.37683	5.40113	-4.5874
H	-4.85974	2.27772	-2.83612	H	-3.96991	2.50551	-2.72353
H	-7.57754	-0.94582	-7.9468	H	-5.84732	-1.38987	-1.53357
H	-6.76859	-1.63278	-9.39032	H	-6.77229	-1.11243	-0.02789
H	-7.5138	-0.02843	-9.46485	H	-5.10833	-0.52747	-0.16875
H	-1.94364	1.22001	-6.77626	H	-8.46655	3.89477	-3.21058
H	-3.04873	0.97006	-5.40353	H	-7.93082	2.75269	-4.46821
H	-3.08988	2.49169	-6.30073	H	-6.81112	3.9599	-3.84574

E 4iz-pzH (1)				*E* 4iz-pzH (2)			
C	5.06883	7.35954	-1.37365	C	0.44736	2.48114	-0.15631
C	4.00617	6.43712	-1.07879	C	1.81277	2.10161	-0.62478
C	2.92454	7.22509	-0.67784	N	2.14359	0.84638	-0.80827
N	3.36987	8.498	-0.74966	O	3.49029	0.87468	-1.24609
N	4.66566	8.60675	-1.16853	C	3.92562	2.14286	-1.31188
N	3.91164	5.05755	-1.12832	C	5.32303	2.37117	-1.75422
N	4.96647	4.45745	-1.5095	C	2.90095	2.98322	-0.9269
C	4.85893	3.07506	-1.55493	N	2.81516	4.36436	-0.81581
C	3.78684	2.15604	-1.25678	N	3.8867	4.98072	-1.12843
N	4.16558	0.91704	-1.4534	C	3.8228	6.35722	-1.02478
O	5.51264	0.98373	-1.89155	C	2.77937	7.21114	-0.63287
C	5.8999	2.26612	-1.94376	C	1.38263	6.94532	-0.19299
C	2.39957	2.44159	-0.78486	N	3.30415	8.45139	-0.72952
C	7.28307	2.57675	-2.37951	N	4.60211	8.48742	-1.15097
C	1.55372	6.82865	-0.25115	C	4.9246	7.21245	-1.33292
C	6.45636	7.07781	-1.84542	C	6.28278	6.806	-1.80064
H	1.85203	1.50507	-0.65987	H	-0.16349	1.58868	-0.01071
H	1.87705	3.08116	-1.50107	H	-0.03954	3.13461	-0.88647
H	2.42344	2.98395	0.16397	H	0.50633	3.03388	0.78614
H	7.46599	2.20527	-3.39307	H	5.81049	1.41849	-1.9707
H	8.01642	2.10732	-1.71574	H	5.88238	2.90794	-0.98232
H	7.42397	3.65841	-2.36258	H	5.34016	3.008	-2.64359
H	1.33813	7.1559	0.77207	H	1.37429	6.30375	0.69257
H	1.47636	5.74124	-0.28924	H	0.83245	6.40372	-0.96752
H	0.78681	7.25397	-0.90804	H	0.85504	7.87683	0.0353
H	2.86004	9.33972	-0.53319	H	2.83695	9.32139	-0.52473
H	6.99868	8.01787	-1.96851	H	6.90449	7.69057	-1.95036
H	6.44247	6.53686	-2.7962	H	6.22285	6.25064	-2.74208
H	6.98812	6.43978	-1.13342	H	6.76828	6.15016	-1.07091

E 4iz-pzH (3)				*E* 4iz-pzH (4)			
C	4.99878	7.38689	-1.30945	C	4.85569	7.30436	-1.25844
C	3.97866	6.40782	-1.04679	C	3.79284	6.38974	-0.98587
C	2.86106	7.13235	-0.62492	C	2.70889	7.18172	-0.57575
N	3.24761	8.42581	-0.65563	N	3.17604	8.44762	-0.62936
N	4.5381	8.60701	-1.06681	N	4.4733	8.55677	-1.03921
N	3.95231	5.02836	-1.14104	N	3.92642	5.02151	-1.13376
N	5.03927	4.49665	-1.53843	N	2.88875	4.33955	-0.85149
C	5.00264	3.11178	-1.6297	C	3.04722	2.97005	-1.0075
C	6.12516	2.32992	-2.05747	C	4.15904	2.15105	-1.42868
N	5.84742	1.04887	-2.06165	N	3.83259	0.88201	-1.42066
O	4.50254	0.9563	-1.62593	O	2.48217	0.82673	-0.98955
C	4.01607	2.18196	-1.37439	C	2.0407	2.07005	-0.75197
C	7.4706	2.83298	-2.46274	C	5.53319	2.56424	-1.84002
C	2.61174	2.27953	-0.90492	C	0.64389	2.25274	-0.28767
C	1.50926	6.66008	-0.21528	C	1.32219	6.84133	-0.15478
C	6.39794	7.18129	-1.78526	C	6.23428	6.97599	-1.72803
H	8.1185	1.99922	-2.73818	H	6.12267	1.68189	-2.09766
H	7.9317	3.39245	-1.64343	H	6.02381	3.11516	-1.03353
H	7.38254	3.51695	-3.31205	H	5.4879	3.24253	-2.6959
H	2.16908	1.28397	-0.8335	H	0.47426	1.72769	0.65804
H	2.02404	2.89628	-1.5913	H	-0.06677	1.85625	-1.02017
H	2.57113	2.77229	0.071	H	0.45519	3.31779	-0.14524
H	1.27671	6.94444	0.81701	H	1.33634	6.1752	0.71244
H	1.4827	5.57196	-0.28773	H	0.80021	6.30263	-0.95059
H	0.72447	7.06965	-0.86099	H	0.75303	7.74163	0.0977
H	2.6994	9.23621	-0.41475	H	2.66762	9.28815	-0.40106
H	6.89886	8.14742	-1.87802	H	6.81507	7.89273	-1.84503
H	6.40972	6.6675	-2.7509	H	6.20611	6.44834	-2.6866
H	6.95539	6.54379	-1.09285	H	6.74481	6.32031	-1.01563

Z 4iz-pzH (1)				*Z* 4iz-pzH (2)			
C	0.26798	4.28986	4.18306	C	-2.70074	-0.11702	-0.34101
C	1.53299	4.33251	3.39984	C	-1.36185	0.38014	-0.46579
N	2.44363	3.33503	3.34124	C	-1.15516	1.09787	0.6883
N	3.52875	3.62843	2.57589	O	-2.26437	1.01733	1.45272
C	3.28962	4.83979	2.07909	N	-3.23675	0.22892	0.80523
C	4.33066	5.51555	1.24496	N	-0.61924	0.30314	-1.66118
C	2.02427	5.32904	2.55606	N	0.6292	0.12233	-1.67541
N	1.38543	6.57576	2.47913	C	1.37064	-0.25381	-0.54278
N	1.35104	7.27826	1.43189	C	1.13221	-1.22872	0.43307
C	1.73751	6.79036	0.16853	N	2.28351	-1.28206	1.14946
C	2.33181	7.59202	-0.77313	N	3.25103	-0.42813	0.72185
O	2.45442	6.91443	-1.92665	C	2.71101	0.18836	-0.32404
N	1.86547	5.6421	-1.78471	C	-0.02014	-2.13433	0.70171
C	1.45112	5.56633	-0.54055	C	3.4447	1.2321	-1.09792
C	0.72412	4.34044	-0.08901	C	-0.03805	1.93313	1.1951
C	2.85375	8.97742	-0.69435	C	-3.44007	-0.95489	-1.32951
H	0.40368	3.77485	5.13908	H	0.31011	-3.05135	1.19926
H	-0.53379	3.78015	3.6358	H	-0.5078	-2.41458	-0.23527
H	-0.05929	5.31445	4.37448	H	-0.77869	-1.65374	1.32893
H	4.10493	5.44572	0.17666	H	3.2973	1.07983	-2.17102
H	5.29747	5.04119	1.4248	H	4.51021	1.19359	-0.86435
H	4.4034	6.57711	1.49716	H	3.0773	2.23749	-0.86136
H	0.36789	3.78816	-0.9607	H	-4.47892	-1.07862	-1.01976
H	-0.12882	4.61043	0.53933	H	-2.97989	-1.94439	-1.42271
H	1.37816	3.69093	0.49833	H	-3.40325	-0.48686	-2.3173
H	2.41589	9.46851	0.1775	H	0.46875	2.42897	0.36377
H	3.94437	8.98663	-0.58535	H	-0.41787	2.68757	1.88744
H	2.60182	9.53722	-1.59898	H	0.71012	1.32141	1.7116
H	2.4135	2.44893	3.82079	H	2.49806	-1.92449	1.89655

Z 4iz-pzH (3)				*Z* 4iz-pzH (4)			
C	1.46245	5.51193	-0.46585	C	1.32967	5.63318	-0.66325
C	1.67309	6.84756	0.01201	C	1.60707	6.81716	0.11444
C	2.4827	7.42553	-0.93684	C	2.15213	7.69294	-0.78863
O	2.71767	6.52439	-1.91287	O	2.25821	7.09296	-1.98782
N	2.05205	5.3162	-1.6204	N	1.70704	5.79838	-1.91067
N	1.29492	7.26876	1.29954	N	1.2508	7.20224	1.42448
N	0.88448	8.43641	1.54347	N	1.36718	6.42868	2.4145
C	0.53224	9.36911	0.55359	C	2.06558	5.21364	2.35562
C	0.6905	10.73449	0.79559	C	3.31762	4.90074	1.81301
N	0.12931	11.35397	-0.26683	N	3.54937	3.62391	2.20894
N	-0.44138	10.5011	-1.15857	N	2.55911	3.08246	2.96783
C	-0.19941	9.28204	-0.68256	C	1.65989	4.05362	3.083
C	1.35499	11.42614	1.93402	C	4.31934	5.69051	1.04152
C	-0.76628	8.08982	-1.38393	C	0.38634	3.87331	3.83915
C	3.14145	8.74925	-1.06681	C	2.63902	9.08545	-0.63716
C	0.65518	4.44419	0.1925	C	0.63315	4.37039	-0.2692
H	0.83722	12.35322	2.19871	H	5.33174	5.31505	1.21983
H	2.39861	11.67566	1.70843	H	4.28868	6.73949	1.34444
H	1.35189	10.75769	2.79807	H	4.1217	5.65314	-0.03516
H	-0.01808	7.57932	-1.9977	H	0.18157	4.75668	4.45085
H	-1.58518	8.40928	-2.03153	H	0.45045	2.99091	4.47839
H	-1.14735	7.35892	-0.66491	H	-0.46497	3.74572	3.16067
H	0.77705	3.49697	-0.33513	H	0.26708	3.86232	-1.1633
H	-0.40694	4.71067	0.19871	H	-0.21212	4.59156	0.3884
H	0.96676	4.32899	1.23453	H	1.30018	3.69628	0.27375
H	3.40239	9.13942	-0.08067	H	2.2224	9.50594	0.28075
H	4.04669	8.65898	-1.67129	H	3.73263	9.12273	-0.57063
H	2.4686	9.47261	-1.54153	H	2.33503	9.69555	-1.49194
H	0.06561	12.34581	-0.4346	H	4.38991	3.08882	2.05398

E 3iz-pzH (1)				*E* 3iz-pzH (2)			
O	-2.60334	0.87745	-7.63328	C	-3.52322	2.44759	-6.2201
C	-2.91914	1.11913	-6.33725	C	-2.85164	1.12626	-6.33979
C	-2.5853	0.0351	-5.58657	C	-2.86097	-0.00878	-5.59411
C	-2.02052	-0.87635	-6.5284	C	-1.9806	-0.88564	-6.30275
N	-2.03332	-0.39242	-7.75885	N	-1.58554	-2.19928	-6.02719
C	-3.53052	2.44008	-6.03278	N	-2.12015	-2.64835	-4.9637
N	-1.57485	-2.16198	-6.17925	C	-1.77097	-3.94055	-4.63554
N	-0.74253	-2.6582	-7.00247	C	-0.91874	-4.85701	-5.27792
C	-0.3367	-3.94365	-6.71997	C	-0.10476	-4.75929	-6.51915
C	-0.71965	-4.83697	-5.70298	N	-0.96488	-5.95671	-4.50174
N	0.00455	-5.94671	-5.94159	N	-1.77282	-5.84708	-3.403
N	0.83203	-5.86581	-7.02904	C	-2.26749	-4.61908	-3.47916
C	0.62845	-4.64631	-7.50814	C	-3.21276	-4.0904	-2.45196
C	-1.68553	-4.71216	-4.57857	N	-1.48187	-0.32132	-7.38865
C	1.34979	-4.14326	-8.71377	O	-2.03411	0.95645	-7.41356
H	-3.7587	2.51103	-4.96784	H	-4.14987	2.46914	-5.32687
H	-4.45616	2.58098	-6.59989	H	-4.15162	2.64604	-7.09426
H	-2.85079	3.25568	-6.29983	H	-2.78653	3.25443	-6.15124
H	-2.7004	-0.10979	-4.52447	H	-3.39577	-0.21998	-4.68378
H	-2.67527	-4.44214	-4.95659	H	0.60589	-3.93164	-6.44603
H	-1.38655	-3.90514	-3.90381	H	-0.74265	-4.5326	-7.37769
H	-1.75789	-5.64564	-4.01171	H	0.44091	-5.68869	-6.70947
H	-0.01497	-6.8051	-5.41208	H	-0.47237	-6.82354	-4.65541
H	1.97113	-4.93649	-9.13329	H	-3.39839	-4.85167	-1.69219
H	1.98606	-3.28987	-8.45935	H	-4.16496	-3.80325	-2.90862
H	0.64186	-3.79775	-9.4725	H	-2.80486	-3.19708	-1.96897

***E* 3iz-pzH (3)**				***E* 3iz-pzH (4)**			
O	-2.36972	0.92528	-7.69631	C	2.57952	-1.05758	0.00000
C	-2.80344	1.18782	-6.43863	C	1.76124	0.12682	0.00000
C	-2.59214	0.09531	-5.65654	C	2.65314	1.20446	0.00000
C	-1.97811	-0.84565	-6.53593	N	3.87836	0.64373	0.00000
N	-1.84573	-0.36838	-7.76228	N	3.86239	-0.7258	0.00000
C	-3.38682	2.53463	-6.19972	N	0.39676	0.32428	0.00000
N	-1.62191	-2.14438	-6.13304	N	-0.30315	-0.73628	0.00000
N	-0.78914	-2.70928	-6.90783	C	-1.67608	-0.46323	0.00000
C	-0.4534	-4.00132	-6.57145	C	-2.3819	0.78125	0.00000
C	0.46618	-4.7262	-7.33802	C	-3.68822	0.40972	0.00000
N	0.55239	-5.92768	-6.73555	O	-3.77271	-0.94748	0.00000
N	-0.24295	-6.05203	-5.6263	N	-2.49517	-1.50012	-0.00001
C	-0.86274	-4.88669	-5.51307	C	-4.97544	1.15433	0.00000
C	1.21571	-4.32182	-8.55912	C	2.38679	2.66953	0.00000
C	-1.83005	-4.6364	-4.40592	C	2.15563	-2.48739	0.00000
H	-3.71369	2.62243	-5.16212	H	-4.78626	2.22917	0.00001
H	-4.24704	2.70842	-6.85403	H	-5.57095	0.90308	0.88355
H	-2.65186	3.32029	-6.40255	H	-5.57096	0.90308	-0.88354
H	-2.81966	-0.04043	-4.61154	H	-1.95872	1.77121	0.00001
H	0.91797	-3.30707	-8.82753	H	2.80989	3.15613	0.88566
H	0.99796	-4.98597	-9.40255	H	1.30743	2.8274	0.00000
H	2.298	-4.336	-8.38965	H	2.80989	3.15612	-0.88567
H	1.11935	-6.71373	-7.012	H	4.7692	1.11501	0.00000
H	-1.91348	-5.52847	-3.78147	H	3.03741	-3.13161	0.00001
H	-2.814	-4.37174	-4.80376	H	1.53616	-2.70898	-0.87359
H	-1.50905	-3.78798	-3.79469	H	1.53615	-2.70898	0.8736

Z 3iz-pzH (1)				*Z* 3iz-pzH (2)			
C	-3.63707	5.04755	-3.38554	O	-4.84235	4.30283	-5.15615
C	-4.34551	3.9384	-4.07801	C	-4.13523	3.98247	-4.04519
C	-4.60789	2.64265	-3.75824	C	-4.43667	2.71217	-3.66429
C	-5.3317	2.14779	-4.88516	C	-5.38162	2.27741	-4.64833
N	-5.97343	0.88695	-4.90071	N	-5.62156	3.20531	-5.55166
N	-6.04901	0.14307	-5.9124	C	-3.22127	5.02592	-3.50828
C	-5.37138	0.29161	-7.11923	N	-5.90125	0.97023	-4.84512
C	-4.12083	0.8098	-7.50438	N	-6.39551	0.31902	-3.88793
C	-3.02413	1.52087	-6.79084	C	-6.70583	0.87889	-2.64377
N	-4.00127	0.4659	-8.80418	C	-7.3165	2.09177	-2.29023
N	-5.05817	-0.22744	-9.31472	N	-7.5194	1.98843	-0.95631
C	-5.88698	-0.3574	-8.28979	N	-7.10087	0.81389	-0.4076
C	-7.19401	-1.06572	-8.41302	C	-6.62694	0.11979	-1.43377
N	-5.51095	3.07431	-5.80792	C	-7.79994	3.26079	-3.07874
O	-4.87996	4.22089	-5.29088	C	-6.06111	-1.25181	-1.27354
H	-3.2595	4.70652	-2.42016	H	-2.7363	4.66793	-2.59857
H	-4.3113	5.89366	-3.21975	H	-3.77039	5.94392	-3.27638
H	-2.79448	5.40776	-3.98451	H	-2.44838	5.27861	-4.24115
H	-4.34754	2.1036	-2.86159	H	-4.05346	2.15753	-2.8238
H	-2.90845	1.14089	-5.77388	H	-8.1078	2.94969	-4.07824
H	-3.2428	2.59057	-6.71752	H	-7.0152	4.01323	-3.20694
H	-2.07244	1.38922	-7.31474	H	-8.65482	3.7328	-2.58494
H	-7.21038	-1.66456	-9.3255	H	-6.35431	-1.66726	-0.30776
H	-8.02581	-0.35413	-8.44951	H	-4.96688	-1.24211	-1.32739
H	-7.35799	-1.70783	-7.5435	H	-6.41745	-1.90174	-2.07785
H	-3.20234	0.63621	-9.39582	H	-8.00322	2.65134	-0.37013

Z 3iz-pzH (3)				*Z* 3iz-pzH (4)			
O	-4.93633	4.34683	-5.12352	O	-4.61979	4.03479	-5.51419
C	-4.51639	3.96992	-3.89111	C	-3.99287	3.95907	-4.31523
C	-4.85103	2.669	-3.6767	C	-4.28928	2.77161	-3.72227
C	-5.48854	2.27373	-4.89055	C	-5.14691	2.12427	-4.66833
N	-5.55962	3.2616	-5.76233	N	-5.33653	2.85418	-5.74849
C	-3.8244	5.00374	-3.0765	C	-3.15941	5.1256	-3.91987
N	-6.16907	1.04716	-5.06333	N	-5.61996	0.7879	-4.65085
N	-6.14527	0.38345	-6.13152	N	-6.13963	0.2963	-3.61537
C	-5.30505	0.58905	-7.22544	C	-6.53166	1.04295	-2.49701
C	-5.71009	0.05944	-8.45754	C	-6.52236	0.44502	-1.23327
N	-4.68251	0.27937	-9.29832	N	-7.07079	1.35765	-0.40527
N	-3.60125	0.85902	-8.70295	N	-7.50725	2.48226	-1.03828
C	-3.9618	1.07002	-7.44397	C	-7.18038	2.31657	-2.31534
C	-6.99609	-0.58635	-8.83567	C	-6.00934	-0.88889	-0.81674
C	-2.96285	1.64551	-6.49444	C	-7.60764	3.33275	-3.3253
H	-3.5539	4.59398	-2.10191	H	-2.72045	4.95632	-2.93519
H	-4.47018	5.87433	-2.92484	H	-3.76171	6.03878	-3.8828
H	-2.91379	5.34911	-3.57649	H	-2.35235	5.28902	-4.64108
H	-4.68044	2.06517	-2.80008	H	-3.96036	2.40296	-2.76507
H	-7.68851	0.13086	-9.29078	H	-4.98788	-0.82999	-0.42367
H	-7.46802	-0.97628	-7.93144	H	-5.9926	-1.54375	-1.69091
H	-6.83997	-1.405	-9.54503	H	-6.64253	-1.33784	-0.04535
H	-1.96466	1.54779	-6.92566	H	-8.4234	3.92732	-2.90962
H	-2.98596	1.13152	-5.53003	H	-7.94594	2.85442	-4.24734
H	-3.16438	2.70398	-6.30931	H	-6.78977	4.00678	-3.59444
H	-4.63079	0.04835	-10.27797	H	-7.21739	1.27306	0.58851

E 4iz-pzMe (1)				*E* 4iz-pzMe (2)			
C	5.10692	7.28209	-1.38485	C	-2.88899	-2.62158	0.00001
C	4.0348	6.37998	-1.07679	C	-3.08044	-1.14128	0.00000
C	2.97941	7.18953	-0.64431	N	-4.27966	-0.61124	0.00000
N	3.43855	8.45899	-0.709	O	-4.06951	0.78906	-0.00001
N	4.73052	8.53416	-1.15727	C	-2.75335	1.05613	0.00000
N	3.91087	5.00453	-1.13796	C	-2.33835	2.48042	-0.00001
N	4.94419	4.38507	-1.54822	C	-2.05932	-0.13641	0.00000
C	4.80418	3.00578	-1.60402	N	-0.70227	-0.43025	0.00000
C	3.71812	2.10847	-1.29073	N	0.05286	0.59818	0.00000
N	4.06367	0.86291	-1.50697	C	1.40805	0.33609	0.00000
O	5.40169	0.90278	-1.97408	C	2.12499	-0.87346	0.00000
C	5.81718	2.17691	-2.02307	C	1.6514	-2.28572	-0.00001
C	2.3484	2.42092	-0.78566	N	3.42863	-0.51114	0.00000
C	7.19727	2.46023	-2.48642	N	3.61522	0.84698	0.00000
C	1.61151	6.81504	-0.19215	C	2.39403	1.36514	0.00001
C	2.73289	9.67612	-0.36839	C	2.16819	2.84098	0.00001
C	6.47868	6.98099	-1.8898	H	-3.85591	-3.12751	0.00001
H	1.78234	1.4959	-0.65722	H	-2.32012	-2.93362	0.88108
H	1.82518	3.07927	-1.4841	H	-2.32011	-2.93363	-0.88105
H	2.40541	2.9535	0.16728	H	-3.21669	3.12906	-0.00003
H	7.93434	1.96819	-1.84342	H	-1.71794	2.695	-0.87515
H	7.36273	3.53831	-2.46249	H	-1.71796	2.69501	0.87513
H	7.34973	2.09484	-3.50724	H	1.0206	-2.47569	-0.87317
H	1.42225	7.13731	0.83832	H	1.0206	-2.4757	0.87315
H	1.51135	5.72991	-0.23955	H	2.48344	-2.9938	-0.00001
H	0.83761	7.26395	-0.82548	C	4.59851	-1.36831	-0.00001
H	7.03569	7.9123	-2.01463	H	3.12638	3.36393	0.00001
H	6.43487	6.45095	-2.84589	H	1.59548	3.15003	0.88031
H	7.01434	6.3254	-1.1968	H	1.59548	3.15004	-0.88028
H	1.8424	9.80042	-0.99258	H	4.62222	-2.00184	0.89148
H	2.43135	9.67274	0.68379	H	4.62223	-2.00181	-0.89151
H	3.41849	10.50297	-0.54628	H	5.46669	-0.71171	0.00001

E 4iz-pzMe (3)				*E* 4iz-pzMe (4)			
C	5.05584	7.3193	-1.39372	C	4.8878	7.23731	-1.39207
C	4.02596	6.36773	-1.08845	C	3.82951	6.34258	-1.05951
C	2.93341	7.1266	-0.65586	C	2.76013	7.1586	-0.65158
N	3.33323	8.41593	-0.71781	N	3.22144	8.42628	-0.75986
N	4.62123	8.55187	-1.1644	N	4.51442	8.49707	-1.2089
N	3.97067	4.98883	-1.15371	N	3.95488	4.97084	-1.16067
N	5.03723	4.42564	-1.56508	N	2.9245	4.29892	-0.82854
C	4.9681	3.04016	-1.62586	C	3.08195	2.92474	-0.94103
C	6.06286	2.22476	-2.06268	C	4.18463	2.09303	-1.36127
N	5.75762	0.95017	-2.03325	N	3.86154	0.82437	-1.30135
O	4.42206	0.89622	-1.56485	O	2.52275	0.78186	-0.8351
C	3.96819	2.13783	-1.32819	C	2.08449	2.03255	-0.62961
C	7.40891	2.68959	-2.50952	C	5.54726	2.49344	-1.8209
C	2.57765	2.27576	-0.82857	C	0.69927	2.22943	-0.13713
C	1.58394	6.68797	-0.20617	C	1.38844	6.80923	-0.18667
C	2.57166	9.59854	-0.37606	C	2.52503	9.66364	-0.4647
C	6.43994	7.08028	-1.89732	C	6.25348	6.88774	-1.88408
H	8.03214	1.83629	-2.78212	H	6.13202	1.6034	-2.06253
H	7.90108	3.25576	-1.7132	H	6.05663	3.07273	-1.04656
H	7.31561	3.35771	-3.37081	H	5.47923	3.14177	-2.69821
H	2.11564	1.29173	-0.72594	H	-0.02893	1.80722	-0.83737
H	1.98707	2.8909	-1.51392	H	0.5124	3.29866	-0.0271
H	2.57032	2.78928	0.13742	H	0.55405	1.73751	0.83024
H	1.37837	6.9988	0.82471	H	1.43811	6.16552	0.6963
H	1.53503	5.59939	-0.25583	H	0.86134	6.23444	-0.95364
H	0.79048	7.10114	-0.83963	H	0.80245	7.69901	0.05535
H	6.95549	8.03533	-2.0207	H	6.83349	7.79774	-2.04878
H	6.42089	6.54769	-2.85269	H	6.20043	6.32598	-2.82204
H	7.00265	6.44641	-1.2057	H	6.7808	6.25656	-1.1618
H	1.67707	9.68262	-1.00111	H	1.63814	9.77652	-1.09512
H	2.26953	9.57957	0.6758	H	2.22609	9.70623	0.58676
H	3.21854	10.45643	-0.55189	H	3.22286	10.47218	-0.67486

Z **4iz-pzMe (1)**				*Z* **4iz-pzMe (2)**			
C	0.33611	4.37606	4.21343	C	2.4259	7.22271	-1.03699
C	1.57846	4.39764	3.39423	C	1.69735	6.74417	0.02601
N	2.47194	3.3836	3.3222	C	1.51461	5.35261	-0.26618
N	3.5345	3.67415	2.51939	N	2.04429	5.0301	-1.4222
C	3.29893	4.88785	2.02563	O	2.64095	6.21177	-1.90507
C	4.32894	5.54763	1.16511	N	1.38012	7.3163	1.27538
C	2.05481	5.39022	2.53443	N	0.93323	8.49122	1.39151
N	1.42395	6.64005	2.46655	C	0.48759	9.25603	0.30133
N	1.37283	7.33991	1.41745	C	0.66947	10.66642	0.22202
C	1.72856	6.84335	0.14802	N	0.04078	11.16358	-0.83812
C	2.31093	7.63362	-0.81021	N	-0.57711	10.09703	-1.42114
O	2.39864	6.95015	-1.96405	C	-0.34519	8.92367	-0.77462
N	1.79837	5.68564	-1.80299	C	1.49147	11.51849	1.13065
C	1.41183	5.62008	-0.54926	C	-0.97973	7.63622	-1.176
C	0.68299	4.40404	-0.07465	C	-1.45248	10.32592	-2.55369
C	2.85167	9.01272	-0.7491	C	0.79615	4.34922	0.57221
H	0.52616	4.00633	5.22631	C	3.02959	8.53874	-1.36346
H	-0.43899	3.74024	3.76835	H	-0.37034	7.08946	-1.90517
H	-0.05573	5.39327	4.27695	H	-1.97103	7.79729	-1.60889
H	4.07996	5.47524	0.10207	H	-1.09307	6.99006	-0.30305
H	5.29436	5.06361	1.32638	H	1.31039	11.243	2.17359
H	4.41931	6.6096	1.40979	H	1.24717	12.57197	0.98187
H	0.29863	3.85224	-0.93466	H	2.56367	11.38622	0.94342
H	-0.15015	4.68562	0.57467	H	0.9268	3.34749	0.15993
H	1.34553	3.75046	0.49863	H	-0.27484	4.57426	0.6174
H	2.44044	9.51214	0.13096	H	1.1761	4.37853	1.59734
H	3.94463	9.00906	-0.66573	H	2.30101	9.19973	-1.8459
H	2.58538	9.57273	-1.64952	H	3.36173	9.03798	-0.45004
C	2.41443	2.09893	3.98908	H	3.88334	8.40397	-2.03108
H	1.5501	1.51951	3.64985	H	-1.22693	9.62689	-3.36308
H	3.33031	1.56732	3.73655	H	-2.50513	10.21945	-2.27066
H	2.35417	2.22582	5.07401	H	-1.27409	11.34526	-2.89189

Z **4iz-pzMe (3)**				*Z* **4iz-pzMe (4)**			
C	1.46552	5.52595	-0.48224	C	1.29588	5.70557	-0.72458
C	1.66834	6.87062	-0.0271	C	1.57012	6.88589	0.06021
C	2.47308	7.43709	-0.98677	C	2.09766	7.7734	-0.84166
O	2.71274	6.52081	-1.94802	O	2.19596	7.18479	-2.04731
N	2.0553	5.31381	-1.63391	N	1.65788	5.88468	-1.97465
N	1.29137	7.31052	1.2548	N	1.22739	7.26013	1.37795
N	0.87443	8.48005	1.48113	N	1.35746	6.47944	2.36176
C	0.51212	9.39455	0.47998	C	2.05658	5.26702	2.27944
C	0.6528	10.76717	0.70236	C	3.3023	4.96609	1.71387
N	0.08914	11.37817	-0.36559	N	3.54183	3.67139	2.05146
N	-0.46154	10.49587	-1.246	N	2.5464	3.11805	2.80238
C	-0.2118	9.28385	-0.75529	C	1.65323	4.08781	2.9684
C	1.30242	11.47604	1.83854	C	4.29022	5.80546	0.97716
C	0.02311	12.79764	-0.64619	C	4.73602	2.89369	1.78642
C	-0.76876	8.08045	-1.44584	C	0.38381	3.87868	3.72461
C	3.12054	8.76362	-1.1416	C	2.574	9.16889	-0.68372
C	0.66564	4.46472	0.1954	C	0.61421	4.43284	-0.33628
H	0.69074	12.3106	2.19654	H	4.16765	5.71815	-0.10881
H	2.28509	11.8795	1.56513	H	5.31654	5.52421	1.2299
H	1.44251	10.76553	2.65567	H	4.15292	6.85613	1.23849
H	-0.01628	7.56838	-2.0532	H	0.17315	4.74543	4.35765
H	-1.5886	8.38757	-2.09836	H	0.45762	2.98227	4.34322
H	-1.14657	7.35386	-0.72073	H	-0.46941	3.75826	3.04696
H	0.79207	3.50938	-0.31632	H	0.2475	3.92894	-1.23254
H	-0.39801	4.72518	0.19914	H	-0.22933	4.64046	0.32791
H	0.9796	4.36906	1.23871	H	1.2903	3.76049	0.19735
H	3.38243	9.17249	-0.16336	H	2.16231	9.5784	0.24138
H	4.02368	8.67099	-1.74891	H	3.66787	9.21526	-0.62688
H	2.43873	9.4732	-1.62453	H	2.2569	9.78321	-1.5307
H	-0.4392	13.33607	0.18601	H	4.99857	2.94102	0.7266
H	1.02087	13.21047	-0.82664	H	5.58305	3.24932	2.38284
H	-0.58675	12.91842	-1.53995	H	4.51207	1.86428	2.06101

***E* 3iz-pzMe (1)**				***E* 3iz-pzMe (2)**			
C	-2.45554	1.35821	0.11843	C	-3.53108	2.50337	-6.22157
C	-1.45416	0.34377	0.04243	C	-2.84983	1.18623	-6.33373
C	-2.15545	-0.875	-0.04373	C	-2.87783	0.04349	-5.60059
N	-3.46004	-0.53243	-0.01231	C	-1.97122	-0.82271	-6.28954
N	-3.66702	0.82332	0.0858	N	-1.58013	-2.13726	-6.01404
N	-0.10772	0.62493	0.04336	N	-2.14831	-2.59756	-4.97166
N	0.66085	-0.38939	0.03635	C	-1.80978	-3.88963	-4.63932
C	2.02521	-0.06495	-0.03685	C	-0.93529	-4.80735	-5.2538
C	3.06133	-0.95597	0.37558	C	-0.08796	-4.67533	-6.47079
C	4.20788	-0.28028	0.09524	N	-1.00046	-5.91821	-4.4902
O	3.89557	0.91501	-0.46272	N	-1.85363	-5.79664	-3.41972
N	2.50695	1.05426	-0.55061	C	-2.34618	-4.56972	-3.50581
C	5.65417	-0.5802	0.26828	C	-3.32663	-4.04758	-2.50841
C	-1.6611	-2.27468	-0.15785	N	-1.44065	-0.2448	-7.35307
C	-4.61915	-1.40163	-0.08681	O	-1.99786	1.03156	-7.38249
C	-2.24754	2.83208	0.22782	H	-4.18602	2.51267	-5.34864
H	5.78169	-1.5721	0.70548	H	-4.13226	2.70859	-7.11318
H	6.12956	0.15522	0.92535	H	-2.80083	3.31272	-6.12067
H	6.176	-0.55131	-0.69358	H	-3.44038	-0.17972	-4.71007
H	2.94602	-1.9313	0.82031	H	0.60614	-3.83755	-6.36027
H	-1.14073	-2.57371	0.75751	H	-0.70701	-4.43343	-7.33919
H	-0.92194	-2.34468	-0.95973	H	0.47748	-5.58764	-6.67394
H	-2.47364	-2.97938	-0.34987	C	-0.2964	-7.17415	-4.67018
H	-3.20648	3.34952	0.16315	H	-3.53751	-4.81317	-1.75951
H	-1.58711	3.18939	-0.56718	H	-4.26317	-3.75854	-2.99532
H	-1.77002	3.08949	1.17863	H	-2.93671	-3.15664	-2.0065
H	-4.65592	-1.92765	-1.04539	H	-0.55713	-7.63613	-5.62656
H	-4.61173	-2.13376	0.72537	H	0.7863	-7.02653	-4.62511
H	-5.49522	-0.76271	0.00826	H	-0.60753	-7.82606	-3.85598

***E* 3iz-pzMe (3)**				***E* 3iz-pzMe (4)**			
O	-2.3125	1.01944	-7.6743	O	-2.20466	1.10266	-7.33018
C	-2.80357	1.25269	-6.43218	C	-3.01362	1.16749	-6.23931
C	-2.63185	0.1409	-5.66801	C	-2.94634	-0.00415	-5.55598
C	-1.98003	-0.78148	-6.54034	C	-2.02793	-0.79063	-6.32125
N	-1.78943	-0.27502	-7.74731	N	-1.57957	-0.14083	-7.381
C	-3.39386	2.59555	-6.18756	C	-3.7579	2.43953	-6.0403
N	-1.64689	-2.09012	-6.15177	N	-1.55313	-2.09204	-6.12279
N	-0.78195	-2.64133	-6.90262	N	-2.04442	-2.62724	-5.07901
C	-0.4657	-3.94101	-6.58441	C	-1.62041	-3.91186	-4.82161
C	0.486	-4.65444	-7.32545	C	-2.07777	-4.62488	-3.70614
N	0.55811	-5.87161	-6.75099	N	-1.46282	-5.82357	-3.76159
N	-0.29403	-6.00976	-5.68169	N	-0.63215	-5.9479	-4.84822
C	-0.91919	-4.84738	-5.56646	C	-0.71453	-4.79688	-5.49983
C	1.27898	-4.22221	-8.5082	C	-3.03646	-4.21814	-2.64295
C	1.35162	-7.00835	-7.16907	C	-1.61711	-6.94486	-2.85854
C	-1.92798	-4.62123	-4.49158	C	0.06886	-4.56159	-6.74687
H	-3.76793	2.65947	-5.16424	H	-4.37113	2.37762	-5.13961
H	-4.22263	2.78846	-6.87624	H	-4.40991	2.64745	-6.89477
H	-2.64836	3.38323	-6.33719	H	-3.06766	3.28299	-5.9372
H	-2.90807	-0.01919	-4.63828	H	-3.4551	-0.29188	-4.65184
H	1.00575	-3.1941	-8.75042	H	-3.37202	-3.20039	-2.8465
H	1.07924	-4.85388	-9.38097	H	-2.57303	-4.24097	-1.64997
H	2.35603	-4.26179	-8.30891	H	-3.91244	-4.87611	-2.61451
H	-2.03676	-5.52729	-3.89149	H	0.66031	-5.44893	-6.98259
H	-2.89561	-4.34496	-4.92047	H	0.72912	-3.69703	-6.63385
H	-1.63009	-3.78777	-3.84873	H	-0.59457	-4.32679	-7.58404
H	0.91002	-7.50104	-8.04192	H	-1.47316	-6.62641	-1.82239
H	2.3674	-6.6899	-7.41523	H	-2.60858	-7.39908	-2.95764
H	1.37935	-7.70995	-6.33661	H	-0.85667	-7.67746	-3.124

Z **3iz-pzMe (1)**				*Z* **3iz-pzMe (2)**			
C	-3.65544	5.04956	-3.36308	O	-4.89473	4.2654	-5.23131
C	-4.35017	3.92591	-4.04609	C	-4.14672	3.99675	-4.13366
C	-4.61157	2.63472	-3.70846	C	-4.41772	2.73874	-3.69431
C	-5.31991	2.11777	-4.83571	C	-5.38955	2.25412	-4.6281
N	-5.95154	0.85099	-4.83064	N	-5.67163	3.14396	-5.5575
N	-6.05422	0.10544	-5.84043	C	-3.22929	5.0712	-3.66842
C	-5.41239	0.26207	-7.06408	N	-5.90134	0.93555	-4.76112
C	-4.16332	0.76728	-7.47091	N	-6.33441	0.30398	-3.76072
C	-3.04192	1.40855	-6.72958	C	-6.58239	0.89607	-2.51861
N	-4.08029	0.48079	-8.7904	C	-7.18914	2.11254	-2.16737
N	-5.17808	-0.17377	-9.27984	N	-7.29132	2.07654	-0.81587
C	-5.9768	-0.33265	-8.23578	N	-6.80394	0.92518	-0.2641
C	-7.3036	-1.00571	-8.34771	C	-6.39835	0.19119	-1.29185
N	-5.48994	3.02858	-5.77606	C	-7.75974	3.21953	-2.987
O	-4.87053	4.18603	-5.27001	C	-7.93278	3.04809	0.04802
H	-3.28758	4.72579	-2.38803	C	-5.78867	-1.15959	-1.11371
H	-4.33557	5.89489	-3.21848	H	-2.70907	4.75442	-2.76285
H	-2.80754	5.40461	-3.95766	H	-3.78245	5.99084	-3.45299
H	-4.36097	2.11126	-2.79988	H	-2.48487	5.30507	-4.43621
H	-3.02499	1.06927	-5.69287	H	-3.99933	2.22255	-2.8462
H	-3.16101	2.49753	-6.71244	H	-7.97918	2.87169	-3.99648
H	-2.07654	1.16773	-7.18242	H	-7.05618	4.05447	-3.08309
H	-7.35293	-1.58607	-9.27104	H	-8.6846	3.60079	-2.54475
H	-8.11848	-0.27376	-8.3551	H	-6.01515	-1.54462	-0.11769
H	-7.46703	-1.66083	-7.48778	H	-4.69967	-1.12705	-1.23049
C	-2.9679	0.73375	-9.68508	H	-6.17342	-1.84862	-1.87095
H	-2.67935	1.78758	-9.65591	H	-7.57213	4.05568	-0.17287
H	-2.10168	0.11592	-9.42601	H	-9.02166	3.02377	-0.06667
H	-3.3059	0.476	-10.68717	H	-7.6744	2.78333	1.07193

Z **3iz-pzMe (3)**				*Z* **3iz-pzMe (4)**			
O	-4.86999	4.41369	-5.06664	O	-4.66682	4.05431	-5.57921
C	-4.48761	4.01297	-3.82973	C	-4.01285	4.06161	-4.39251
C	-4.89146	2.73065	-3.62348	C	-4.22698	2.88324	-3.74854
C	-5.5317	2.3704	-4.84712	C	-5.064	2.15021	-4.64991
N	-5.53965	3.36242	-5.71723	N	-5.31821	2.82523	-5.75223
C	-3.75512	5.00814	-3.00234	C	-3.24126	5.28889	-4.0602
N	-6.2744	1.18265	-5.0309	N	-5.45143	0.78912	-4.57027
N	-6.26616	0.51335	-6.09676	N	-5.92267	0.30858	-3.50583
C	-5.39702	0.66338	-7.17451	C	-6.34631	1.07194	-2.41281
C	-5.79939	0.13172	-8.40943	C	-6.29403	0.52362	-1.12542
N	-4.75294	0.28784	-9.24351	N	-6.88506	1.42678	-0.31423
N	-3.66477	0.8393	-8.62587	N	-7.37975	2.50338	-0.99383
C	-4.03389	1.08407	-7.37557	C	-7.06012	2.31187	-2.26923
C	-7.10606	-0.46703	-8.79339	C	-5.70521	-0.76662	-0.67402
C	-4.68316	-0.01403	-10.65817	C	-7.02925	1.37152	1.12616
C	-3.02203	1.6281	-6.4209	C	-7.55976	3.26708	-3.30549
H	-3.51874	4.58248	-2.02568	H	-2.77288	5.1836	-3.08013
H	-4.35797	5.90991	-2.85604	H	-3.89508	6.16659	-4.04365
H	-2.8216	5.30854	-3.48897	H	-2.46012	5.47075	-4.80518
H	-4.76443	2.11727	-2.74607	H	-3.85819	2.57174	-2.78556
H	-7.67147	-0.67254	-7.88284	H	-5.51973	-1.38848	-1.55198
H	-6.97302	-1.39958	-9.35171	H	-6.37984	-1.30073	0.00304
H	-7.69758	0.21356	-9.41689	H	-4.75314	-0.62393	-0.14914
H	-2.02418	1.4831	-6.8396	H	-8.40329	3.82696	-2.89691
H	-3.07706	1.12723	-5.45092	H	-7.88208	2.73966	-4.20619
H	-3.1804	2.69623	-6.2497	H	-6.78689	3.97845	-3.6096
H	-5.07547	-1.01481	-10.85547	H	-7.46221	0.41571	1.43298
H	-5.25093	0.71475	-11.24592	H	-6.06348	1.50199	1.62517
H	-3.63363	0.0298	-10.94509	H	-7.69743	2.18219	1.4121

Crystallographic Data

X-Ray crystal structure analysis of *E*-**4iz-4iz**:

A yellow, needle-shaped crystal was mounted on a loop with perfluoroether oil. The Crystals were crystallized from cyclohexane / DCM. Data for rav10738 were collected from a single crystal in 22.65 hours at 100(2) K on a Bruker D8 VENTURE KAPPA diffractometer with a microfocus sealed tube using a multilayer mirror as monochromator and a Bruker PHOTON III CPAD detector. The diffractometer was equipped with an Oxford Cryostream 1000 low temperature device and used Cu K_α radiation (λ = 1.54178 Å). All data were integrated with SAINT V8.41 (Bruker, *SAINT*, *V8.41*, Bruker AXS Inc.,

Madison, Wisconsin, USA), yielding 14141 reflections of which 973 where independent and 91.4% were greater than 2σ(F^2). A Multi-Scan absorption correction using SADABS 2016/2 was applied.[19] The structure was solved by Intrinsic Phasing methods with SHELXT 2018/2 and refined by full-matrix least-squares methods against F^2 using SHELXL-2019/2.[20,21] All non-hydrogen atoms were refined with anisotropic displacement parameters. All hydrogen atoms were refined isotropic on calculated positions using a riding model with their U_{iso} values constrained to 1.5 times the U_{eq} of their pivot atoms for terminal sp^3 carbon atoms and 1.2 times for all other carbon atoms. Crystallographic data for the structures reported in this paper have been deposited with the Cambridge Crystallographic Data Centre.[22] CCDC 2463597 contains the supplementary crystallographic data for this paper. This data can be obtained free of charge from The Cambridge Crystallographic Data Centre via www.ccdc.cam.ac.uk/structures. This report and the CIF file were generated using FinalCif.[23]

Table S5: Crystal data and structure refinement for rav10738.

CCDC number	2463597
Empirical formula	$C_{10}H_{12}N_4O_2$
Formula weight	220.24
Temperature [K]	100(2)
Crystal system	monoclinic
Space group (number)	$P2_1/n$ (14)
a [Å]	4.1319(2)
b [Å]	15.3888(7)
c [Å]	8.5819(4)
α [°]	90
β [°]	101.967(2)
γ [°]	90
Volume [Å^3]	533.82(4)
Z	2
ρ_{calc} [gcm^{-3}]	1.37
μ [mm^{-1}]	0.827
F(000)	232
Crystal size [mm^3]	0.043×0.054×0.129
Crystal colour	yellow
Crystal shape	needle
Radiation	Cu K_α (λ=1.54178 Å)
2θ range [°]	11.50 to 137.01
Index ranges	−4 ≤ h ≤ 4
Reflections collected	14141
Independent	973
Completeness to	100.0
Data / Restraints /	973 / 0 / 75
Goodness-of-fit on F^2	1.095
Final R indexes	R_1 = 0.0317
Final R indexes	R_1 = 0.0342
Largest peak/hole	0.21/−0.19

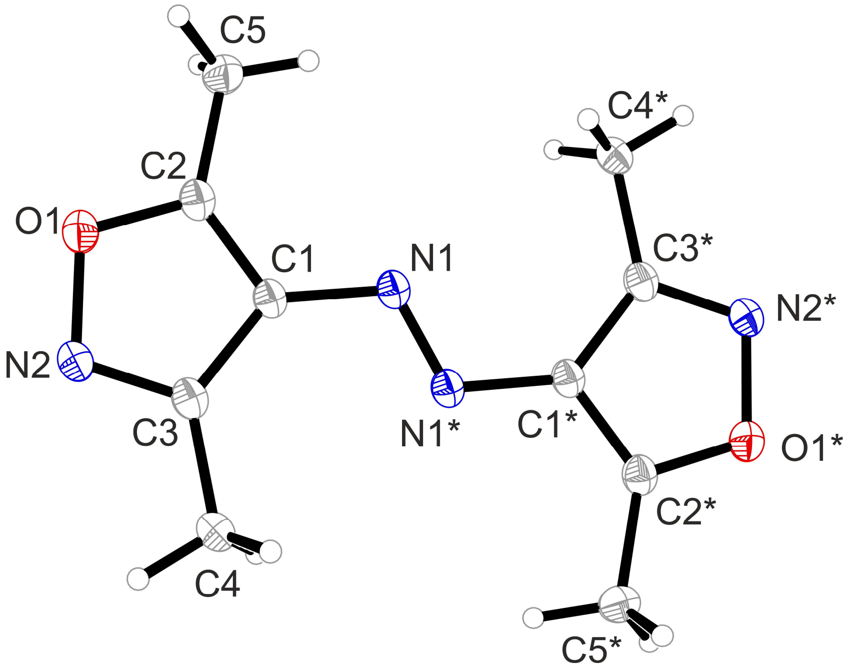

a)

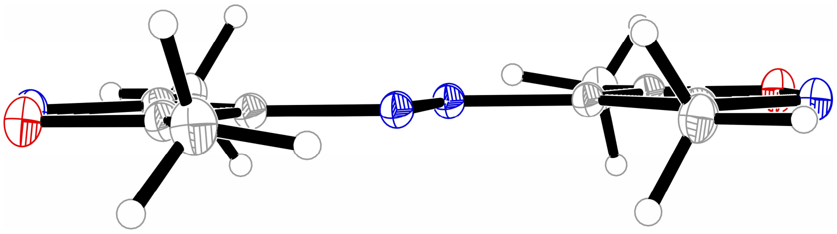

b)

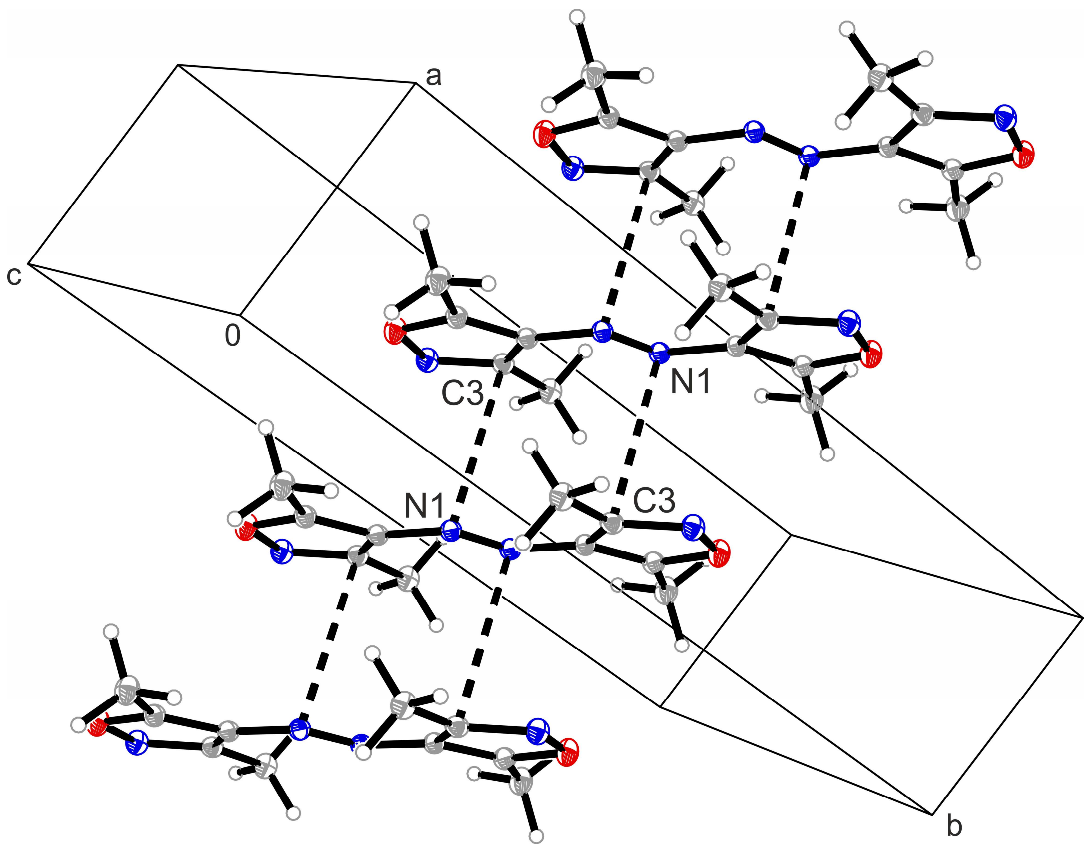

c)

Figure S44: XP structure of compound *E*-**4iz-4iz**, thermal ellipsoids drawn at the 50% probability. Compound *E*-**4iz-4iz** crystallized with one half-molecule in the asymmetric unit, the whole molecule is generated by inversion symmetry. a) View perpendicular on the plane of the molecule *E*-**4iz-4iz**. b) Side view along the molecular plane of compound *E*-**4iz-4iz.** c) Excerpt of the packing diagram of compound *E*-**4iz-4iz** representing the formation of stairs-like chain along the *a*-axis via $\pi\cdots\pi$ interactions (short distance between the aromatic units: N1···C3 3.331 Å).

X-Ray crystal structure analysis of *E*-**3iz-4iz**:

A yellow, needle-shaped crystal was mounted on a MiTeGen micromount with perfluoroether oil. The crystals were crystallised from acetonitrile. Data for rav10796 were collected from a single crystal in 4.72 hours at 100(2) K on a Bruker D8 VENTURE KAPPA diffractometer with a microfocus sealed tube using a multilayer mirror as monochromator and a Bruker PHOTON III CPAD detector. The diffractometer was equipped with an Oxford Cryostream 1000 low temperature device and used Mo K_α radiation (λ = 0.71073 Å). All data were integrated with SAINT V8.41 (Bruker, *SAINT*, *V8.41*, Bruker AXS Inc., Madison, Wisconsin, USA), yielding 13266 reflections of which 2232 where independent and 94.6% were greater than 2σ(F^2). A Multi-Scan absorption correction using SADABS 2016/2 was applied.[19] The structure was solved by Intrinsic Phasing methods with SHELXT 2018/2 and refined by full-matrix least-squares methods against F^2 using SHELXL-2019/2.[20,21] All non-hydrogen atoms were refined with anisotropic displacement parameters. All hydrogen atoms were refined isotropic on calculated positions using a riding model with their U_{iso} values constrained to 1.5 times the U_{eq} of their pivot atoms for terminal sp^3 carbon atoms and 1.2 times for all other carbon atoms. Crystallographic data for the structures reported in this paper have been deposited with the Cambridge Crystallographic

Data Centre.[22] CCDC 2463597 contains the supplementary crystallographic data for this paper. This data can be obtained free of charge from The Cambridge Crystallographic Data Centre via www.ccdc.cam.ac.uk/structures. This report and the CIF file were generated using FinalCif.[23]

Table S6: Crystal data and structure refinement for rav10796.

CCDC number	2463597
Empirical formula	$C_9H_{10}N_4O_2$
Formula weight	206.21
Temperature [K]	100(2)
Crystal system	orthorhombic
Space group (number)	$Pna2_1$ (33)
a [Å]	20.9217(13)
b [Å]	4.4554(2)
c [Å]	10.4846(6)
α [°]	90
β [°]	90
γ [°]	90
Volume [Å^3]	977.32(9)
Z	4
ρ_{calc} [gcm^{-3}]	1.401
μ [mm^{-1}]	0.104
F(000)	432
Crystal size [mm^3]	0.041×0.068×0.3
Crystal colour	yellow
Crystal shape	needle
Radiation	Mo K_α (λ=0.71073 Å)
2θ range [°]	3.89 to 55.02 (0.77 Å)
Index ranges	−27 ≤ h ≤ 27
Reflections collected	13266
Independent	2232
Completeness to	99.9
Data / Restraints /	2232 / 1 / 139
Goodness-of-fit on F^2	1.035
Final R indexes	R_1 = 0.0306
Final R indexes	R_1 = 0.0332
Largest peak/hole	0.20/−0.20
Flack X parameter	-0.2(4)

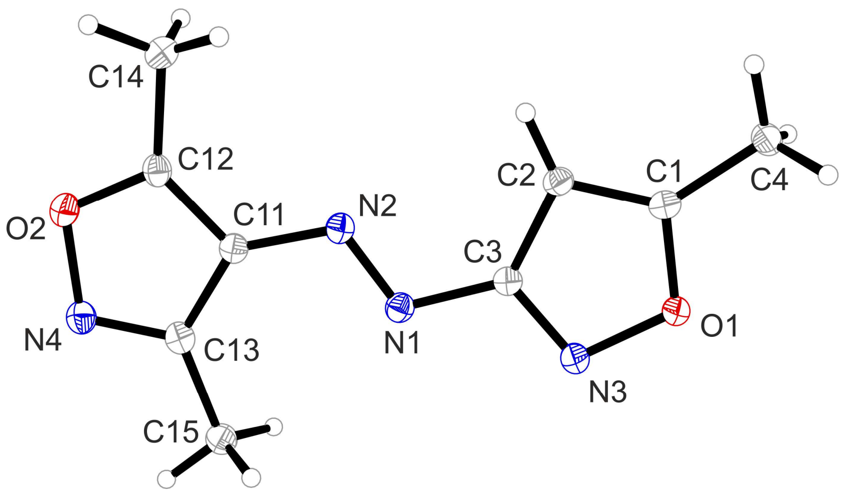

a)

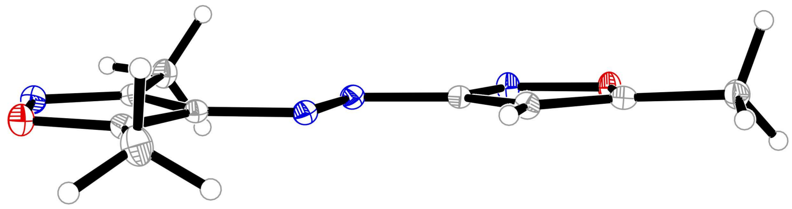

b)

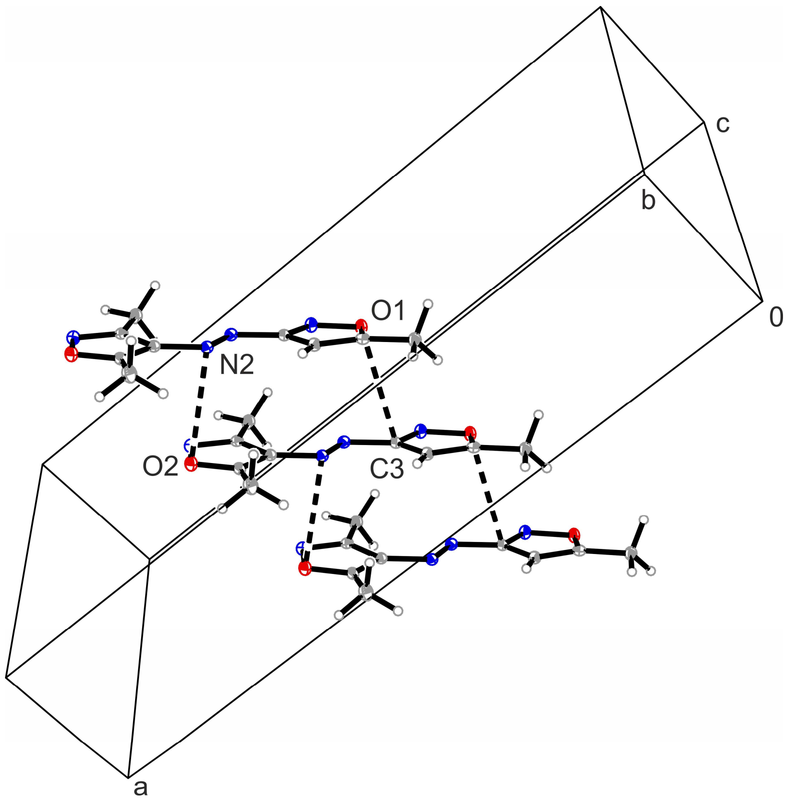

c)

Figure S45: XP structure of compound *E*-**3iz-4iz**, thermal ellipsoids drawn at the 50% probability. a) View perpendicular on the plane of the molecule *E*-**3iz-4iz**. b) Side view along the molecular plane of compound *E*-**3iz-4iz.** c) Excerpt of the packing diagram of compound *E*-**3iz-4iz** representing the formation of stairs-like chain along the *b*-axis via $\pi\cdots\pi$ interactions (short distance between the aromatic units: O1···C3 3.389 Å; N2···O2 3.235 Å).

References

[1] M. Canton, A. B. Grommet, L. Pesce, J. Gemen, S. Li, Y. Diskin-Posner, A. Credi, G. M. Pavan, J. Andréasson, R. Klajn, *J. Am. Chem. Soc.* **2020**, *142*, 14557–14565.

[2] A. B. Nepomnyashchii, M. Bröring, J. Ahrens, A. J. Bard, *J. Am. Chem. Soc.* **2011**, *133*, 8633–8645.

[3] R. S. L. Gibson, J. Calbo, M. J. Fuchter, *ChemPhotoChem* **2019**, *3*, 372–377.

[4] S. Ludwanowski, M. Ari, K. Parison, S. Kalthoum, P. Straub, N. Pompe, S. Weber, M. Walter, A. Walther, *Chem. Eur. J.* **2020**, *26*, 13203–13212.

[5] J. Gemen, J. R. Church, T.-P. Ruoko, N. Durandin, M. J. Białek, M. Weißenfels, M. Feller, M. Kazes, M. Odaybat, V. A. Borin, R. Kalepu, Y. Diskin-Posner, D. Oron, M. J. Fuchter, A. Priimagi, I. Schapiro, R. Klajn, *Science* **2023**, *381*, 1357–1363.

[6] M. J. Frisch, G. W. Trucks, H. B. Schlegel, G. E. Scuseria, M. A. Robb, J. R. Cheeseman, G. Scalmani, V. Barone, G. A. Petersson, H. Nakatsuji, X. Li, M. Caricato, A. V. Marenich, J. Bloino, B. G. Janesko, R. Gomperts, B. Mennucci, H. P. Hratchian, J. V. Ortiz, A. F. Izmaylov, J. L. Sonnenberg, D. Williams-Young, F. Ding, F. Lipparini, F. Egidi, J. Goings, B. Peng, A. Petrone, T. Henderson, D. Ranasinghe, V. G. Zakrzewski, J. Gao, N. Rega, G. Zheng, W. Liang, M. Hada, M. Ehara, K. Toyota, R. Fukuda, J. Hasegawa, M. Ishida, T. Nakajima, Y. Honda, O. Kitao, H. Nakai, T. Vreven, K. Throssell, J. A. Montgomery Jr., J. E. Peralta, F. Ogliaro, M. J. Bearpark, J. J. Heyd, E. N. Brothers, K. N. Kudin, V. N. Staroverov, T. A. Keith, R. Kobayashi, J. Normand, K. Raghavachari, A. P. Rendell, J. C. Burant, S. S. Iyengar, J. Tomasi, M. Cossi, J. M. Millam, M. Klene, C. Adamo, R. Cammi, J. W. Ochterski, R. L. Martin, K. Morokuma, O. Farkas, J. B. Foresman, D. J. Fox, *Gaussian 16 Revision B.01*, **2016**.

[7] C. Lee, W. Yang, R. G. Parr, *Phys. Rev. B* **1988**, *37*, 785–789.

[8] A. D. Becke, *J. Chem. Phys.* **1993**, *98*, 5648–5652.

[9] A. D. Becke, *Phys. Rev. A* **1988**, *38*, 3098–3100.

[10] W. J. Hehre, R. Ditchfield, J. A. Pople, *J. Chem. Phys.* **1972**, *56*, 2257–2261.

[11] P. C. Hariharan, J. A. Pople, *Theor. Chim. Acta* **1973**, *28*, 213–222.

[12] M. M. Francl, W. J. Pietro, W. J. Hehre, J. S. Binkley, M. S. Gordon, D. J. DeFrees, J. A. Pople, *J. Chem. Phys.* **1982**, *77*, 3654–3665.

[13] R. Ditchfield, W. J. Hehre, J. A. Pople, *J. Chem. Phys.* **1971**, *54*, 724–728.

[14] S. Grimme, S. Ehrlich, L. Goerigk, *J. Comput. Chem.* **2011**, *32*, 1456–1465.

[15] S. Grimme, J. Antony, S. Ehrlich, H. Krieg, *J. Chem. Phys.* **2010**, *132*, 154104.

[16] M. N. Vo, M. Call, C. Kowall, J. K. Johnson, *Ind. Eng. Chem. Res.* **2022**, 61, 2359–2365.

[17] W. Humphrey, A. Dalke, K. Schulten, *J. Mol. Graph.* **1996**, *14*, 33–38.

[18] VMD **http://www.ks.uiuc.edu/Research/vmd/**

[19] L. Krause, R. Herbst-Irmer, G. M. Sheldrick, D. Stalke, *J. Appl. Crystallogr.* **2015**, *48*, 3–10.

[20] G. M. Sheldrick, *Acta Crystallogr A Found Adv* **2015**, *71*, 3–8.

[21] G. M. Sheldrick, *Acta Crystallogr C Struct Chem* **2015**, *71*, 3–8.

[22] C. R. Groom, I. J. Bruno, M. P. Lightfoot, S. C. Ward, *Acta Cryst. B: Struct. Sci., Cryst. Eng. Mater.* **2016**, *72*, 171–179.

[23] D. Kratzert, *FinalCif*, *(Bruker Edition)*, https://dkratzert.de/finalcif.html.
